# Pooled Prevalence of Adverse Pregnancy and Neonatal Outcomes in Malawi, South Africa, Uganda, and Zimbabwe: Results From a Systematic Review and Meta-Analyses to Inform Trials of Novel HIV Prevention Interventions During Pregnancy

**DOI:** 10.3389/frph.2021.672446

**Published:** 2021-11-26

**Authors:** Erica M. Lokken, Anya Mathur, Katherine E. Bunge, Lee Fairlie, Bonus Makanani, Richard Beigi, Lisa Noguchi, Jennifer E. Balkus

**Affiliations:** ^1^Department of Global Health, University of Washington, Seattle, WA, United States; ^2^Department of Obstetrics, Gynecology and Reproductive Sciences, University of Pittsburgh, Pittsburgh, PA, United States; ^3^Wits Reproductive Health and HIV Institute, Faculty of Health Sciences, University of the Witwatersrand, Johannesburg, South Africa; ^4^Department of Obstetrics and Gynecology, College of Medicine, University of Malawi, Blantyre, Malawi; ^5^Department of Epidemiology, Johns Hopkins Bloomberg School of Public Health, Baltimore, MD, United States; ^6^Department of Epidemiology, University of Washington, Seattle, WA, United States; ^7^Vaccine and Infectious Diseases Division, Fred Hutchinson Cancer Research Center, Seattle, WA, United States

**Keywords:** Malawi, Zimbabwe, Uganda, South Africa, pregnancy complications, pregnancy outcomes, neonatal outcomes

## Abstract

**Background:** Robust data summarizing the prevalence of pregnancy and neonatal outcomes in low- and middle-income countries are critically important for studies evaluating investigational products for HIV prevention and treatment in pregnant and breastfeeding women. In preparation for studies evaluating the safety of the dapivirine vaginal ring for HIV prevention in pregnancy, we conducted a systematic literature review and meta-analyses to summarize the prevalence of pregnancy and neonatal outcomes in Malawi, South Africa, Uganda, and Zimbabwe.

**Methods:** Ten individual systematic literature reviews were conducted to identify manuscripts presenting prevalence data for 12 pregnancy and neonatal outcomes [pregnancy loss, stillbirth, preterm birth, low birthweight (LBW), neonatal mortality, congenital anomaly, chorioamnionitis, postpartum endometritis, postpartum hemorrhage, gestational hypertension, preeclampsia/eclampsia, and preterm premature rupture of membranes (PPROM)]. Studies included in the meta-analyses were published between January 1, 1998, and July 11, 2018, provided numerator and denominator data to support prevalence estimation, and included women of any HIV serostatus. Random-effects meta-analyses were conducted to estimate the pooled prevalence and 95% confidence interval (CI) for each outcome overall, by country, and by HIV status.

**Results:** A total of 152 manuscripts were included across the 12 outcomes. Overall, the frequency of stillbirth (*n* = 75 estimates), LBW (*n* = 68), and preterm birth (*n* = 67) were the most often reported. However, fewer than 10 total manuscripts reported prevalence estimates for chorioamnionitis, endometritis, or PPROM. The outcomes with the highest pooled prevalence were preterm birth (12.7%, 95%CI 11.2–14.3), LBW (11.7%, 95%CI 10.6–12.9), and gestational hypertension (11.4%, 95%CI 7.8–15.7). Among the outcomes with the lowest pooled prevalence estimates were neonatal mortality (1.7%, 95%CI 1.4–2.1), pregnancy loss [1.9%, 95%CI 1.1–2.8, predominately studies (23/29) assessing losses occurring after the first trimester], PPROM (2.2%, 95%CI 1.5–3.2), and stillbirth (2.5%, 95%CI 2.2–2.7).

**Conclusions:** Although this review identified numerous prevalence estimates for some outcomes, data were lacking for other important pregnancy-related conditions. Additional research in pregnant populations is needed for a thorough evaluation of investigational products, including for HIV prevention and treatment, and to inform better estimates of the burden of adverse pregnancy outcomes globally.

## Introduction

In Sub-Saharan Africa, cisgender women of reproductive age represent the largest proportion of those with new HIV infections, making them a key focus for HIV testing, treatment, and prevention efforts (1). Pregnant and postpartum women, in particular, have higher rates of HIV acquisition compared with non-pregnant women (2–4). Yet, despite the potential increased susceptibility of HIV faced by women during these clinically complex periods of their lives, pregnant and postpartum women are frequently excluded from clinical trials evaluating investigational products for HIV treatment or prevention. This exclusion is not unique to the development of HIV-related interventions, but rather is due to paternalistic regulatory restrictions in place in many countries that aim to protect pregnant women and the fetus (5, 6). As a result of such restrictions, data on the safety of medications used in pregnancy are grossly limited, with the majority of the safety data collected through postmarketing surveillance (7).

There is a scientific and ethical imperative to responsibly include pregnant women in research evaluating the safety and efficacy of investigational products. In line with this imperative, the Microbicide Trials Network (MTN) is conducting the DELIVER study, a phase 3b, randomized, open-label safety trial of the dapivirine vaginal ring (25 mg), and oral preexposure prophylaxis (PrEP) (Truvada: 200 mg emtricitabine [FTC]/300 mg tenofovir disoproxil fumarate [TDF]) for HIV prevention in pregnant cisgender women (MTN-042; ClinicalTrials.gov Number: NCT03965923). The primary objectives of this study are to describe maternal and infant safety and pregnancy outcomes among women randomized to receive the dapivirine vaginal ring or oral Truvada. As all enrolled women will be using an HIV prevention product during pregnancy, the frequency of pregnancy complications, pregnancy outcomes, and neonatal outcomes will be compared with the rates in the general population in Malawi, South Africa, Uganda, and Zimbabwe, where the DELIVER study is being conducted.

Maternal and neonatal outcomes, such as stillbirth, preterm birth, low birthweight (LBW), neonatal mortality, and maternal mortality represent internationally recognized and monitored priority health indicators (8–11). In comparison, a lack of sufficient data has been noted for other important outcomes, such as hypertensive disorders of pregnancy, premature preterm rupture of membranes (PPROM), postpartum hemorrhage (PPH), and congenital anomalies. This presents a challenge not only for allocating resources to improve these outcomes but also for evaluating investigational therapeutics for pregnant women. Robust data summarizing the expected prevalence of these outcomes among women in low and middle income countries (LMICs) are critically important for these studies. To that end, the objective of this systematic literature review and meta-analyses was to estimate the prevalence of 12 pregnancy and neonatal outcomes in Malawi, South Africa, Uganda, and Zimbabwe.

## Methods

### Literature Search, Inclusion and Exclusion Criteria, and Data Abstraction

We conducted 10 individual systematic literature reviews to identify manuscripts presenting prevalence data for 12 pregnancy and neonatal outcomes of interest including pregnancy loss (<20 0/7 weeks), stillbirth (≥20 0/7 weeks), preterm birth, LBW, neonatal mortality, congenital anomaly, chorioamnionitis, postpartum endometritis, PPH, gestational hypertension, preeclampsia/eclampsia, and PPROM (Outcome definitions and outcome-specific exclusion critiera are described in Table 1). Gestational hypertension, preeclampsia, and eclampsia were combined into one search strategy, hypertensive disorders of pregnancy. We also conducted one search for all pregnancy losses, including spontaneous abortion and stillbirth/fetal demise. Maternal mortality, a key pregnancy outcome, was excluded from this review as these estimates are routinely tracked by government agencies and surveillance systems.

**Table 1:** Definitions of pregnancy outcomes, infant outcomes, and pregnancy complications for the DELIVER Study and this systematic review and meta-analysis.

**Category**	**Outcome**	**DELIVER study definition**	**Outcome specific exclusion criteria for systematic review & analysis**
Pregnancy Outcome	Pregnancy loss	Pregnancy loss ≥12 weeks and <20 0/7 weeks	•Includes induced abortions only or unable to exclude induced abortions from prevalence estimate
	Stillbirth	Pregnancy loss ≥20 0/7 weeks, including stillbirth and fetal demise	•Reports term stillbirths only •Includes deaths that occurred shortly after birth
	Preterm live birth	Birth before 37 0/7 weeks, live birth only	•Studies enrolling mothers/infants into prospective follow-up weeks after birth (survival bias)
Infant Outcome	Congenital anomaly	Major anomalies that would be detectable at birth or within the first 28 days, including but not limited to polydactyly, craniofacial defects, neural tube defects/hydrocephalus, anencephaly, heart defects, inguinal/umbilical hernia, micrognathia, and cleft lip/palate	n/a
	Low birthweight	<2,500g	•Only includes full-term infants •Different definition (ex: <2,000 g) or ascertainment (self-report, measurement of chest and head circumference) •Studies enrolling mothers/infants after birth (survival bias)
	Neonatal Mortality	Deaths in the first 28 days of life (Days 0–27)	•Studies that only reported perinatal (stillbirth + early neonatal deaths) or early neonatal deaths (ex: <7 days, <14 days) •Only enrolled and reported mortality among healthy newborns
Pregnancy Complication	Chorioamnionitis	Clinical diagnosis following the following grading criteria: Grade 1: Fever of 100.4°F−100.9°F with more than one of the following: FHR > 160 BPM, maternal HR > 120, uterine tenderness between contractions, purulent AF, or preterm labor Grade 2: Grade 1 plus fever of 101°F−104°F Grade 3: Grade 2 plus fetal distress or fever > 104°F Grade 4: Grade 3 plus fetal demise or maternal symptoms of shock	n/a
	Endometritis	Puerperal sepsis and endometritis following the following grading criteria: Grade 0: None Grade 1: Low grade fever and uterine tenderness, resolved with oral antibiotics Grade 2: Moderate symptoms, treated by ≤ 3 days of parenteral antibiotics Grade 3: Severe symptoms treated with > 3 days of IV antibiotics or addition of heparin Grade 4: Severe infection or infection for which operative intervention is indicated	•Studies using the term “puerperal sepsis” unless further defined or included endometritis/clinical features in definition
	Postpartum Hemorrhage	Grade 1: EBL 500–1,000 mL for vaginal delivery or 1,000–1,500 mL for Cesarean section (CS) or reported as slightly increased Grade 2: EBL > 1,000 mL or vaginal delivery or > 1,500 mL for CS, with or without mild dizziness, no transfusion required Grade 3: Hemorrhage at a level for which transfusion of 1–2 units of packed cells, but no other blood products indicated Grade 4: Hemorrhage with shock or coagulopathy, for which transfusion of > 2 units of packed cells or any amount of other blood components is indicated	•Studies with self-report of hemorrhage (applied at first round of inclusion/exclusion)
	Gestational hypertension	Gestational hypertension	•Studies reporting women with chronic hypertension or unspecified hypertension
	Preeclampsia/ Eclampsia	Preeclampsia or Eclampsia	n/a
	PPROM	PPROM	n/a

Detailed inclusion and exclusion criteria for the systematic review and meta analyses are provided in Table 2 and summarized below. Due to concerns regarding the expected paucity of data for some outcomes, the searches included studies that occurred in the DELIVER study countries (Malawi, South Africa, Uganda, and Zimbabwe) and nine additional countries in eastern and southern Africa (Ethiopia, Kenya, Tanzania, Mozambique, Zambia, Botswana, Lesotho, eSwatini, Namibia). All studies reporting study outcome prevalence data were included in the initial data abstraction phase, regardless of how the pregnancy or neonatal outcome was defined. Studies of individuals with any HIV serostatus were included. Exclusion criteria included studies of pregnancy outcomes among high-risk individuals only (e.g., those with preeclampsia/eclampsia), studies including adolescent pregnancies only, and studies where it was not possible to abstract or calculate the numerator and denominator for prevalence estimates. This included studies where prevalence data were inconsistently presented in the tables and the text. For these cases, two reviewers discussed the data and if a consensus could not be made on the best estimate, the manuscript was excluded. We also excluded study designs that are not conducive to estimates of prevalence, including most case-control studies, case reports/series, commentaries, and qualitative studies. If a case-control study first reported the total population at risk and the total number affected with an outcome prior to identifying their case and control population, the study was included and overall prevalence estimate data were abstracted.

**Table 2:** Inclusion and exclusion criteria.

	**Inclusion criteria**	**Exclusion criteria round 1**	**Additional exclusions prior to meta-analyses**
Population	•Pregnant individuals and their neonates •Any HIV serostatus, including not reported	•Studies including high-risk pregnant individuals only (ex: population of women with pre-eclampsia/eclampsia) •Studies of adolescent pregnancies only	•In cases of multiple manuscripts reporting on the same study population, citation reporting the most complete data for each outcome was selected.
Outcome	•Studies reporting prevalence of 12 pregnancy complications and outcomes (see Table 1)	•Unable to abstract or calculate the numerator and denominator data for prevalence estimates	•Outcome specific exclusion criteria (see Table 1)
Setting	•Malawi, South Africa, Uganda, and Zimbabwe (DELIVER Study countries) •Ethiopia, Kenya, Tanzania, Mozambique, Zambia, Botswana, Lesotho, eSwatini, Namibia (non-DELIVER Study countries)		•Studies from non-DELIVER study countries for outcomes with <10 total manuscripts eligible •If studies included data for a DELIVER study country and a country not included in the DELIVER study but was unable to be disaggregated by country, the study was excluded.
Study Design	•Cross-sectional (including surveillance) •Cohort (prospective or retrospective) •Randomized trial •Pre/post studies •Case-control (only if the overall prevalence of entire cohort outcomes were reported prior to selection of case-control population)	•Study designs not conducive to estimating population estimate of outcome prevalence including most case-control studies, case reports/series, commentaries, qualitative studies	•Studies utilizing data from Demographic and Health Surveys (DHS) or Multiple Indicator Cluster Survey (MICS) data •For randomized trials and pre/post studies, only the placebo or before/pre time period were included in analysis.
Years & Language	•January 1, 1998–July 11, 2018 •English		

MEDLINE (PubMed) was searched for eligible manuscripts published in English between January 1, 1998, and July 11, 2018 (Search Strategies: Supplementary Table A1). Each of the 10 outcome searches was conducted and reviewed separately. One reviewer conducted the title and abstract review for each outcome. Two reviewers assessed all full-text manuscripts to determine inclusion. The references of published systematic reviews and meta-analyses identified in the searches were also reviewed for inclusion. All data were abstracted into a single spreadsheet. The primary reviewer conducted the initial data abstraction for each manuscript. The number of pregnancies or infants with the outcome and total sample size at risk were abstracted for each outcome. The sample at risk was defined as the number of pregnancies or the number of infants depending on the outcome under study. Where appropriate, the sample size at risk was adjusted to account for competing pregnancy outcomes. For example, spontaneous abortions were subtracted from the at-risk denominator for stillbirth and delivery-related outcomes since pregnant individuals who experience pregnancy loss are no longer at risk for these future outcomes. If a study did not report either the numerator or denominator but reported a prevalence estimate, the missing value (numerator or denominator) was calculated for inclusion in the meta-analyses. For studies reporting results for multiple countries, prevalence estimates were disaggregated by the country when possible. The main outcome for each independent search, as well as all other outcomes of interest (Table 1), were abstracted from each manuscript to capture all outcome prevalence data within and across the 10 reviews. Prevalence estimates for subgroups, such as HIV status or study arm for randomized trials, were also abstracted. Additional study characteristics including study design, inclusion and exclusion criteria, population characteristics, location, and methods for ascertaining each outcome were abstracted.

Prior to estimating outcome-specific pooled prevalence estimates, the second round of data review was conducted and additional exclusion criteria were applied. First, manuscripts reporting on duplicate study populations were assessed. For each outcome, only the citation reporting the most detailed outcome and prevalence data was included to minimize overrepresentation from the same study population. Second, studies utilizing demographic and health survey or multiple indicator cluster survey data were excluded as these data were aggregated through another project supporting the DELIVER study. Finally, outcome-specific exclusions were also made (exclusions outlined in Table 1), such as non-standard definitions of the outcome or its ascertainment (e.g., LBW defined as <2,000 g, LBW only among live-born infants). During this phase, a second reviewer reviewed the abstracted data against the original manuscript for all included manuscripts to identify errors. Disagreements were discussed between reviewers one and two, and in cases of non-agreement, JEB was consulted to make the final determination.

After completing the 10 literature reviews and data abstraction, sufficient data were available from studies conducted in the DELIVER study countries (Malawi, Uganda, South Africa, and Zimbabwe) for most outcomes. Therefore, outcome-specific meta-analyses only included studies in these countries. For outcomes with fewer than 10 manuscripts occurring in DELIVER study countries, manuscripts from all countries considered in the preliminary searches were included in the meta-analyses. If studies included data for a DELIVER study country and a country not included in the DELIVER study that was unable to be disaggregated by the country, the study was excluded. Some manuscripts reported prevalence estimates for multiple DELIVER study countries, and such prevalence estimates were disaggregated by country in the outcome-specific meta-analyses when possible.

### Analytic Methods for Meta-Analyses

Meta-analyses were conducted using metaprop_one in Stata 15.1 to estimate the pooled prevalence for each outcome using random-effects weighting and exact methods for 95% confidence interval (CI) estimation (12). The Freeman–Tukey double arcsine transformation was utilized to stabilize variances and to include the studies with 0% prevalence estimates (12, 13). For randomized trials and pre-post studies, the prevalence for the control arm or pre-study period, respectively, were included where possible. If not possible, the overall prevalence estimate was included. Study-specific decisions are described in the Supplementary Material.

Forest plots were generated to summarize pooled prevalence estimates overall, by country, and by HIV status. We also conducted sensitivity analyses, which involved 1) excluding manuscripts with an unspecified definition of the outcome, 2) excluding studies utilizing a study definition that was not consistent with the DELIVER study protocol definitions and 3) excluding outliers. Outliers were defined as studies with a prevalence estimate that was >1.5 times the interquartile range of all included studies (14). Several additional outcome-specific sensitivity and subgroup analyses were conducted, which included restricting to studies of LBW and preterm birth when these outcomes were ascertained for live-births only, assessing antepartum vs. intrapartum stillbirth prevalence estimates, restricting to studies reporting congenital anomalies from randomized trials with rigorous assessment for anomalies, and restricting to studies reporting PPH defined as ≥500 ml of blood loss. Results of sensitivity analyses are provided in the Supplementary Material.

## Results

### Overview of Search Results

Search and review results for the 10 literature searches are presented in Table 3. Across all outcomes, a total of 152 manuscripts were included in the meta-analyses (Supplementary Table A2). There were <10 manuscripts reporting prevalence estimates for chorioamnionitis, endometritis, and PPROM; therefore, studies from all queried countries (not just DELIVER study countries) were included in those meta-analyses. The fewest studies were identified for chorioamnionitis (*n* = 6) and the most for stillbirth (*n* = 71). Although pregnancies occur among cisgender women as well as gender minorities with reproductive potential, the studies included in this review were presumed to evaluate pregnancy outcomes and complications among cisgender women only. Therefore, we use the term “woman/women” when reporting the results. The number of pregnant women/infants included in the meta-analyses ranged from 2,086 for chorioamnionitis to 1,498,361 for stillbirth (Table 4). Results for all meta-analyses overall and by country are presented in Table 4 and results by HIV status are presented in Table 5. Outcome-specific forest plots, results of sensitivity analyses, and citations for all included manuscripts are in the Supplementary Material.

**Table 3:** Results for 10 systematic reviews of pregnancy outcomes, pregnancy complications, and neonatal outcomes in 13 Eastern and Southern African countries to support the DELIVER Study (MTN-042), 1998–2018.

**Review step**	**Chorio-amnionitis**	**Endometritis**	**PPROM**	**Postpartum hemorrhage**	Hypertensive disorders of pregnancy^*^	**Neonatal mortality**	**Low birth weight**	**Congenital anomaly**	**Preterm birth**	**Pregnancy loss & stillbirth**
Titles reviewed	6	43	50	217	549	834	791	620	590	874
Abstracts reviewed	1	12	23	68	124	379	213	75	265	237
Manuscripts reviewed^†^	1	4	9	32	54	211	140	40	189	174
Manuscripts Included From Main Search^‡^	1	4	5	16	19	100	115	15	109	124
Manuscripts Added From Other Searches^§^	5	4	2	17	33	41	60	25	48	73
Total Manuscripts—Abstracted	6	8	7	28	52	141	175	40	157	197
Total Manuscripts Included-Analysis^**^	6	7	7	17	18	26	63	19	64	78

* *This search included gestational hypertension and preeclampsia/eclampsia*.

† *Does not include the number of references from systematic reviews that were reviewed*.

‡ *Including systematic review reference reviews*.

§ *Two recent MTN manuscripts were added to this review by study investigators (15, 16). These manuscripts were published after the searches were conducted*.

** *There were fewer than 10 total manuscripts reporting chorioamnionitis, endometritis, or PPROM. Therefore, studies from all queried countries (not just DELIVER study countries) were included in the meta-analyses. Some manuscripts reported prevalence estimates for multiple DELIVER study countries; such prevalence estimates were disaggregated by country in the outcome specific meta-analyses*.

**TABLE 4A:** The pooled prevalence of pregnancy outcomes, pregnancy complications, and neonatal outcomes.

	**Overall**				
**Outcome**	**N**		**%**	**(95% CI)**	**I^**2**^**
	Estimates^*^	**At Risk^†^**			
Pregnancy Loss	33	49,095	1.9	(1.1, 2.8)	92.2%
Stillbirth	75	1,498,361	2.5	(2.2, 2.7)	98.0%
Preterm Birth	67	134,763	12.7	(11.2, 14.3)	98.4%
Congenital Anomaly	22	402,215	0.4	(0.2, 0.7)	97.9%
Low Birthweight	68	117,578	11.7	(10.6, 12.9)	97.1%
Neonatal Mortality	26	342,853	1.7	(1.4, 2.1)	97.2%
Chorioamnionitis^‡^	6	2,086	16.2	(8.0, 26.7)	96.9%
Endometritis^‡^	7	12,653	3.3	(1.1, 6.6)	98.4%
Postpartum Hemorrhage	17	71,308	4.4	(3.0, 6.0)	98.7%
Gestational Hypertension	14	32,024	11.4	(7.8, 15.7)	99.1%
Preeclampsia/Eclampsia	9	50,234	4.0	(1.9, 6.8)	99.4%
PPROM^‡^	7	26,220	2.2	(1.5, 3.2)	93.3%

* *Estimates refers to the number of prevalence estimates. Some included manuscripts reported prevalence estimates by study country and therefore contributed more than one prevalence estimate to outcome specific meta-analyses*.

† *Women/pregnancies or infants depending on the outcome and the study*.

‡ *There were fewer than 10 total manuscripts reporting chorioamnionitis, endometritis, or PPROM. Therefore, studies from all queried countries (not just DELIVER Study countries) were included in the meta-analyses*.

**TABLE 4B:** DELIVER study country.

Outcome^*^	**Malawi**				**South Africa**				**Uganda**				**Zimbabwe**			
	***N***		**%**	**(95% CI)**	***N***		**%**	**(95% CI)**	***N***		**%**	**(95% CI)**	***N***		**%**	**(95% CI)**
	**Estimates^†^**	**At Risk**			**Estimates**	**At Risk**			**Estimates**	**At Risk**			**Estimates**	**At Risk**		
Pregnancy loss	7	3,956	0.6	(0.2, 1,2)	14	30,080	2.5	(1.1, 4.3)	8	7,382	1.4	(0.7, 2.1)	3	7,520	1.0	(0.0, 3.8)
Stillbirth	18	536,079	2.3	(1.8, 2.9)	26	871,383	2.0	(1.7, 2.4)	23	66,533	3.0	(2.1, 4.1)	6	24,003	2.0	(0.4, 4.7)
Preterm birth	13	9,850	13.5	(9.1, 18.5)	28	85,559	12.6	(10.0, 15.5)	15	11,066	11.4	(9.0, 14.1)	10	28,063	14.6	(12.4, 16.9)
Congenital anomaly	4	27,951	0.2	(0.0, 0.6)	8	312,903	0.2	(0.1, 0.5)	7	60,997	0.6	(0.0, 1.5)	1	31	0.0	(0.0, 11.2)
Low Birthweight	17	14,827	10.4	(8.5, 12.5)	20	54,144	12.7	(10.9, 14.5)	20	19,760	11.9	(9.8, 14.2)	11	28,847	11.8	(8.0, 16.2)
Neonatal Mortality	8	22,030	2.4	(1.4, 3.4)	10	276,251	0.9	(0.6, 1.2)	7	40,616	2.6	(2.3, 3.0)	1	3,956	1.3	(0.9, 1.7)
Chorioamnionitis^‡^	1	676	30.6	(27.2, 34.2)	0	–	–	–	2	423	21.5	(17.7, 25.6)	0	–	–	–
Endometritis^‡^	1	2,791	0.8	(0.5, 1.2)	2	4,197	0.3	(0.2, 0.6)	2	4,428	1.6	(0.4, 3.4)	0	–	–	–
Postpartum Hemorrhage	2	5,875	2.0	(1.7, 2.4)	10	57,046	3.6	(2.0, 5.6)	3	3,564	8.8	(2.5, 18.3)	2	4,823	1.9	(1.5, 2.3)
Gestational hypertension	0	–	–	–	10	23,225	10.0	(5.8, 15.3)	1	418	11.5	(8.6, 14.9)	3	8,381	16.5	(6.5, 29.9)
Preeclampsia/Eclampsia	1	2,791	0.7	(0.4, 1.1)	5	37,650	6.2	(3.0, 10.3)	1	418	4.5	(2.8, 7.0)	2	9,375	1.3	(1.1, 1.5)
PPROM^‡^	0	–	–	–	1	421	0.7	(0.1, 2.1)	2	6,528	2.7	(2.3, 3.2)	0	–	–	–

* *Country specific I^2^ are in the Supplementary Material*.

† *Estimates refers to the number of prevalence estimates. Some included manuscripts reported prevalence estimates by study country and therefore contributed more than one prevalence estimate*.

‡ *There were fewer than 10 total manuscripts reporting chorioamnionitis, endometritis, or PPROM. Therefore, studies from all queried countries (not just DELIVER Study countries) were included in the meta-analyses*.

**TABLE 5:** Pooled prevalence of pregnancy outcomes, pregnancy complications, and neonatal outcomes—By HIV status.

**Outcome**	**Women living with HIV**				**HIV-negative**			
	***N***		**%**	**(95% CI)**	***N***		**%**	**(95% CI)**
	**Estimates**	**At Risk**			**Estimates**	**At Risk**		
Pregnancy loss	11	5,255	0.8	(0.3, 1.5)	4	2,161	3.6	(0.5, 9.1)
Stillbirth	17	13,377	2.9	(2.0, 3.8)	9	9,510	1.9	(1.3, 2.5)
Preterm birth	21	18,592	14.1	(11.0, 17.6)	13	11,108	10.0	(5.7, 15.4)
Congenital anomaly	8	1,846	1.8	(0.5, 3.6)	2	739	0.7	(0.1, 1.6)
Low birthweight	18	17,181	13.7	(11.2, 16.3)	10	20,529	10.0	(7.7, 12.5)
Neonatal mortality	5	6,713	1.0	(0.5, 1.8)	1	11,053	0.5	(0.4, 0.6)
Chorioamnionitis^*^	4	1,171	18.7	(5.6, 36.9)	1	68	5.9	(1.3, 13.0)
Endometritis^*^	3	4,022	2.9	(0.3, 7.6)	1	2,916	0.2	(0.1, 0.4)
Postpartum hemorrhage	5	7,541	4.5	(1.3, 9.4)	4	11,650	5.2	(0.4, 14.0)
Gestational hypertension	3	3,201	9.6	(1.3, 24.3)	4	2,399	5.8	(0.9, 14.3)
Preeclampsia/Eclampsia	3	7,701	2.3	(0.6, 5.2)	3	4,910	5.2	(2.6, 8.4)
PPROM^*^	1	68	10.3	(4.2, 20.1)	2	489	0.9	(0.2, 2.1)

* *There were fewer than 10 total manuscripts reporting chorioamnionitis, endometritis, or PPROM. Therefore, studies from all queried countries (not just DELIVER study countries) were included in the meta-analyses*.

### Pregnancy Outcomes

#### Pregnancy Loss

Twenty-nine manuscripts, contributing 33 total prevalence estimates and 49,095 pregnancies, were included in the pregnancy loss meta-analysis (Supplemental Material Section B). In these studies, pregnancy loss was defined as miscarriage, spontaneous abortion, or pregnancy loss by a specific week of gestation (e.g., <20 or <28 weeks of gestation). A gestational age threshold was not defined for 31% (9/29) of the included studies (Supplementary Table B15). Among the included manuscripts, the majority (23/29) enrolled women predominately after the first trimester (Supplementary Table B15); subsequently, the pooled prevalence estimates reported here primarily reflect those occurring after the first trimester.

The overall pooled prevalence of pregnancy loss including all studies independent of pregnancy loss definition was 1.9% (95%CI 1.1–2.8, I^2^ = 92.2%) (Table 4). The pooled prevalence ranged from 1.0% (95%CI 0.0–3.8) in Zimbabwe to 2.5% (95%CI 1.1–4.3) in South Africa. When restricting to studies defining pregnancy loss as losses occurring at <20 or ≤ 20 weeks of gestation, the overall pooled prevalence was 0.5% (95%CI: 0.0–1.6). The pooled prevalence of pregnancy loss was lower among women living with HIV (0.8%, 95%CI 0.3–1.5; *n* = 8 estimates) than HIV-negative women (3.6%, 95%CI 0.5–9.1; *n* = 4 estimates), although the confidence intervals overlap (Table 5).

#### Stillbirth or Fetal Demise

A total of 1,498,361 pregnancies/infants from 71 manuscripts (75 prevalence estimates) were included in the stillbirth meta-analysis (Supplemental Material Section B). The overall pooled prevalence of stillbirth was 2.5% (95% CI 2.2–2.7, I^2^ = 98.0%) (Table 4). The prevalence was similar in all four DELIVER study countries and was 2.3% (95%CI 1.8, 2.9) in Malawi, 2.0% (95% CI 1.7–2.4) in South Africa, 2.0% (95%CI 0.4–4.7) in Zimbabwe, and 3.0% (95%CI 2.1–4.1) in Uganda. When restricting to studies defining stillbirth as those occurring at >20 or ≥20 weeks of gestation, the pooled prevalence was higher at 3.7% (95%CI 1.4, 4.3; Supplementary Table B11). There was no difference in the prevalence of macerated (antepartum) stillbirth (1.7%, 95%CI 1.1–2.4) vs. fresh (intrapartum) stillbirth (1.8%, 95%CI 1.3–2.4) (Supplementary Tables B9, 10). The overall pooled prevalence of stillbirth was 2.9% (95%CI 2.0–3.8) in women living with HIV and 1.9% (95%CI 1.3–2.5) in HIV-negative women (Table 5).

#### Preterm Birth

Sixty-three manuscripts contributing 67 prevalence estimates and 134,763 pregnancies/infants were included in the preterm birth meta-analysis (Supplemental Material Section C). The overall pooled prevalence of preterm birth was 12.7% (95%CI 11.2–14.3, I^2^ = 98.4%) and ranged from 11.4% (95%CI 9.0–14.1) in Uganda to 14.6% (9%CI 12.4–16.9) in Zimbabwe (Table 4). The overall pooled prevalence of preterm birth was 14.1% (95%CI 11.0–17.6) in women living with HIV and 10.0% (95%CI 5.7–15.4) in HIV-negative women (Table 5).

### Neonatal Outcomes

#### Congenital Anomalies

Nineteen manuscripts (22 prevalence estimates) and 402,215 infants were included in the congenital anomalies meta-analysis (Supplemental Material Section D). Forty-two percent (8/19) of manuscripts reported results of randomized controlled trials. In most studies (84.2%, 16/19), assessment for congenital anomalies was conducted at or near the time of birth only, which may contribute to an underestimate of the true congenital anomaly rate (Supplementary Table D6).

The overall pooled prevalence of congenital anomalies was 0.4% (95%CI 0.2–0.7, I^2^ = 97.9%) and was similar in all the countries (Table 4). However, when restricting to eight estimates from randomized trials, the prevalence increased to 1.5% (95%CI 0.2–3.6; Supplementary Table D4). The overall pooled prevalence of congenital anomalies among women living with HIV was 1.8% (95%CI 0.5–3.6; *n* = 8 estimates). This is higher than the pooled prevalence in HIV-negative women (0.7%, 95%CI 0.1–1.6), but there were few included manuscripts (*n* = 2) (Table 5). The frequencies of specific or system-specific anomalies are summarized in Table 6 and Supplementary Table D5. The most common anomalies were umbilical and inguinal hernias (1.7%, 95%CI 0.7–3.8) and polydactyly and syndactyl (0.7%, 95%CI 0.3–1.2).

**TABLE 6:** Overall pooled prevalence of specific and system-specific congenital anomalies.

Anomaly Type^*^	***N***			**%**	**(95%CI)**
	**Manuscripts**	**Cases**	**At Risk**		
Not defined	8	49	13,372	0.34%	(0.04, 0.82)
Cleft lip and/or palate	8	111	205,537	0.03%	(0.01, 0.05)
Neural tube defects and/or Hydrocephalus	3	182	153,535	0.11%	(0.08, 0.14)
Cardiovascular	3	8	3,502	0.23%	(0.01, 0.65)
Polydactyly and Syndactyly	6	43	4,668	0.70%	(0.30, 1.22)
Musculoskeletal^†^	5	14	5,855	0.20%	(0.07, 0.38)
Umbilical and Inguinal Hernia	4	79	3,609	1.73%	(0.40, 3.81)
Esophageal, gastrointestinal, or anorectal	3	47	182,745	0.02%	(0.0, 0.07)
Genitourinary	2	8	2,662	0.23%	(0.06, 0.48)
Trisomy	3	3	4,850	0.05%	(0.05, 0.15)
Multiple systems	1	10	2,365	0.42%	(0.20, 0.78)
Other^§^	6	123	130,916	0.40%	(0.06, 0.98)

* *Congenital anomalies were grouped into subtypes by common types (ex: neural tube defects) and by system (ex: musculoskeletal). A subgroup was created when there was more than one reported case or study reporting the type/system of the anomaly. When multiple anomalies were listed per infant, the infant was included as one overall infant but was included as a case in each of the anomalies sub-types. If specific anomalies were not specified, such infants were included in the “multiple systems” sub-group. Naevus/birthmarks were excluded when possible for the overall and type specific analyses*.

† *Including talipes equinovarus*.

*Systems not defined*.

§ *Includes singular, or infrequent, reports of rare or non-specific anomalies that did not fit well into other defined sub-groups. Examples include natal tooth, anophthalmia, facial asymmetry, arachnoid cyst, hypopigmented skin, macrocephaly with brain defect, subtle dysmorphism, and plagiocephaly*.

#### Low Birthweight

Sixty-four manuscripts contributing 68 prevalence estimates and 117,583 infants were included in the LBW meta-analysis (Supplemental Material Section E). The overall pooled prevalence was 11.7% (95%CI 10.6–12.9, I^2^ = 97.1%), ranging from 10.4% (95%CI 8.5–12.5) in Malawi to 12.7% (95%CI 10.9–14.5) in South Africa (Table 4). The prevalence of LBW among women living with HIV was 13.7% (95%CI 11.2–16.3) and 10.0% (95%CI 7.7–12.5) among HIV-negative women (Table 5).

#### Neonatal Mortality

A total of 342,853 pregnancies from 26 manuscripts were included in the neonatal mortality meta-analysis (Supplemental Material Section F). The overall pooled prevalence of neonatal mortality was 1.7% (95% CI 1.4–2.1, I^2^ = 97.2%). The country-specific pooled prevalence of neonatal mortality was 2.4% (95%CI 1.4–3.4) in Malawi, 0.9% (95%CI 0.6–1.2) in South Africa, 2.6% (95%CI 2.3–3.0) in Uganda, and 1.3% (95%CI 0.9–1.7) in Zimbabwe (Table 4). Few studies reported neonatal mortality by HIV status of the mothers (Table 5). The prevalence of neonatal mortality among women living with HIV and HIV-negative women was 1.0% (95%CI 0.5–1.8; *n* = 5 estimates) and 0.5% (95%CI 0.4–0.6; *n* = 1 estimate), respectively.

### Pregnancy Complications

#### Gestational Hypertension

Fourteen manuscripts including a total of 32,024 pregnancies were included in the gestational hypertension meta-analysis (Supplemental Material Section J). The overall pooled prevalence of gestational hypertension was 11.4% (95%CI 7.8–15.7, I^2^ = 99.1%). The pooled prevalence was 10.0% (95%CI 5.8–15.3) in South Africa, 11.5% (95%CI 8.6–14.9) in Uganda, and 16.5% (95%CI 6.5–29.9) in Zimbabwe (Table 4). No published data were identified for Malawi. Pregnant women living with HIV had a higher prevalence of gestational hypertension than HIV-negative women (9.6%, 95%CI 1.3–24.3, *n* = 3 vs. 5.8%, 95%CI 0.9–14.3, *n* = 4; Table 5).

#### Preeclampsia/Eclampsia

Nine manuscripts reported data on preeclampsia/eclampsia diagnoses and included 50,234 pregnancies (Supplemental Material Section J). The overall pooled prevalence was 4.0% (95%CI 1.9–6.8; I^2^ = 99.4%). Country-specific pooled prevalence was 0.7% (95%CI 0.4–1.1) in Malawi, 6.2% (95%CI 3.0–10.3) in South Africa, 4.5% (95%CI 2.8–7.0) in Uganda, and 1.3% (95%CI 1.1–1.5) in Zimbabwe (Table 4). Pregnant women living with HIV (*n* = 3 estimates) had a lower prevalence of preeclampsia/eclampsia compared with HIV-negative pregnant women (*n* = 3 estimates) (2.3%, 95%CI 0.6–5.2 vs. 5.2%, 95%CI 2.6–8.4; Table 5).

#### Postpartum Hemorrhage

Seventeen manuscripts including 71,308 pregnancies reported prevalence data on PPH and were included in the meta-analysis (Supplemental Material Section I). The overall pooled prevalence was 4.4% (95%CI 3.0–6.0, I^2^ = 98.7%). Country-specific pooled prevalence estimates were 2.0% (95%CI 1.7–2.4) in Malawi, 3.6% (95%CI 2.0–5.6) in South Africa, 8.8% (95%CI 2.5–18.3) in Uganda, and 1.9% (95%CI 6.5–29.9) in Zimbabwe (Table 4). The pooled prevalence of PPH was similar between women living with HIV and HIV-negative women (4.5%, 95%CI 1.3–9.4 vs. 5.2%, 95%CI 0.4–14.0) (Table 5). When restricting to studies defining PPH as ≥500 mL blood loss (*n* = 7), the pooled prevalence was 7.5% (95%CI 4.6–11.1, Supplementary Figure 14).

#### Chorioamnionitis

A total of 2,086 pregnancies from six manuscripts were included in the chorioamnionitis meta-analysis, including studies from Malawi (*n* = 1), Uganda (*n* = 2), Zambia (*n* = 1), and Kenya (*n* = 2) (Supplemental Material Section G). The pooled prevalence for all studies was 16.2% (95%CI 8.0–26.7, I^2^ = 96.9%) (Table 4). However, most of the studies diagnosed chorioamnionitis using histologic criteria, and 66.7% (4/6) of the studies included women living with HIV who had low CD4 cell count or advanced AIDS (Supplemental Material Notes G1); these study characteristics may result in a biased estimate for general population women. The pooled prevalence of chorioamnionitis in women living with HIV was 18.7% (95%CI 5.6–36.9), whereas it was 5.9% (95%CI 1.3–13.0) in the one study reporting the prevalence among HIV-negative women (Table 5). There was no study assessing chorioamnionitis by clinical criteria among HIV-negative women (Supplemental Material Notes G1).

#### Postpartum Endometritis

Few studies reported data on postpartum endometritis prevalence. This meta-analysis includes seven manuscripts including a total of 12,653 pregnancies from Malawi (*n* = 1), South Africa (*n* = 2), Uganda (*n* = 2), Kenya (*n* = 1), and Ethiopia (*n* = 1) (Supplemental Material Section H). The overall pooled prevalence was 3.3% (95%CI 1.1–6.6, I^2^ = 96.9%) (Table 4). The prevalence was 2.9% (95%CI 0.3–7.6, *n* = 3 estimates) in women living with HIV and 0.2% (95%CI 0.1–0.4, *n* = 1 estimate) in HIV-negative women (Table 5).

#### Premature Preterm Rupture of Membranes

A total of 26,220 pregnancies from seven manuscripts were included in the PPROM meta-analysis (Supplemental Material Section K). The included studies represent data from South Africa (*n* = 1), Uganda (*n* = 2), Ethiopia (*n* = 2), and Kenya (*n* = 2). The overall pooled prevalence was 2.2% (95%CI 1.5–3.2, I^2^ = 93.3%; Table 4). The prevalence was 10.3% (95%CI 4.2–20.1) in the one study reporting PPROM among women living with HIV and 0.9% (95%CI 0.2–2.1) in HIV-negative women (*n* = 2 estimates).

#### Review of Potential Study Bias

A qualitative assessment of these literature reviews highlighted the lack of standard outcome ascertainment and quality control procedures, which affect prevalence estimates. Given the expected paucity of data for numerous outcomes of interest, the inclusion criteria for the reviews and meta-analyses included few restrictions on outcome ascertainment methods (outlined in Table 1). Notably, there are data quality issues in studies relying on routinely collected health data and chart abstraction in LMICs, including missing data and underreporting of pregnancy complications and outcomes occurring outside of health facilities (17, 18). There are also known challenges with measuring many of the included outcomes in LMICs, which contribute to underestimates and misclassification. For example, there are numerous methods for estimating gestational age with variable sensitivity and specificity (e.g., ultrasound, last day of menstrual period, fundal height, Ballard score) (19, 20). LBW estimates are complicated by accuracy and precision errors such as poorly calibrated scales, missing data, and overreporting of infants weighing 2,500 g at delivery (21). Additionally, while we excluded self-reported PPH, accurately estimating blood loss quantity is challenging in most settings (22). There were also varying definitions for many outcomes, which reduces the ability to compare between studies. For example, there was significant variability in pregnancy loss and stillbirth definitions, both in terminology and in the gestational age cutoff for pregnancy loss vs. stillbirth (Supplementary Tables B15, 16). Specifically, while the WHO definition of stillbirth for international comparability is a “baby born with no signs of life at or after 28 weeks' gestation” (23), other organizations and numerous studies, including the DELIVER study, use ≥20 weeks of (or ≥24 weeks) gestation to classify stillbirth/fetal demise (Supplementary Tables B15, 16). In addition, in many cases, outcome definitions were not stated in the included papers.

## Discussion

These systematic literature reviews and meta-analyses assessed the prevalence of 12 pregnancy and neonatal outcomes and pregnancy complications in Malawi, South Africa, Uganda, and Zimbabwe. The preponderance of published manuscripts reported data on the frequency of stillbirth, LBW, and preterm birth, which was expected given their status as priority health indicators (8). However, few studies reported on the prevalence of important pregnancy complications such as chorioamnionitis, postpartum endometritis, and PPROM. In many settings, including in LMICs, diagnoses of pregnancy complications can be challenging to ascertain, resulting in a paucity of data to inform reproductive and perinatal health initiatives, clinical care, and in the evaluation of investigational therapeutics, including those for HIV prevention and treatment, and for pregnant and breastfeeding individuals.

Collectively, the variability in outcome definition and ascertainment across studies reduces the ability to precisely estimate the prevalence of these pregnancy and neonatal outcomes. To address this challenge in the context of evaluating vaccine safety among pregnant people, the Global Alignment of Immunization Safety Assessment in pregnancy project (GAIA) was established in 2015 to “improve the quality of outcome data from clinical vaccine trials in pregnant women with a specific focus on the needs and requirements for safety monitoring in LMICs” (24). As part of the GAIA project, standardized case definitions for common obstetric and neonatal outcomes were established to improve the comparability of adverse outcomes across studies (25). Although some data may not be available from participant medical records to appropriately categorize certain outcomes, it is critical that studies of other biomedical interventions in pregnancy begin to collect data in support of the GAIA definitions to facilitate comparability.

The frequent exclusion of pregnant people from clinical trials of investigational products has created an environment where evidence-based guidelines for medication use during pregnancy are lacking, leading to suboptimal treatment. Data on the effect of pregnancy on drug pharmacokinetics, pharmacodynamics, and safety profiles often do not exist or are collected postmarketing, leaving patients and providers wary of medication use during pregnancy and while breastfeeding (5, 26, 27). Clearly, there is an ethical and public health imperative to include pregnant and breastfeeding women in clinical research (28). This need certainly extends to purposefully establishing the safety and efficacy of new HIV treatment and prevention methods in pregnant individuals with or at risk for HIV (5, 29). This can be illustrated by the 2019 finding from the Tsepamo study in Botswana that periconceptual dolutegravir use by women living with HIV may increase neural tube defects (30, 31). Sequential product development strategies that include safety studies among pregnant people are critical for ensuring that initial safety data on use in pregnancy are available for patients and providers at or near the time of product licensure (32). Since many pregnancy and neonatal outcomes are rare, the collection of additional postmarketing safety data must continue to be an important part of monitoring use in pregnant people.

This project had several strengths. Since the original literature searches included studies from the 13 countries in Sub-Saharan Africa, we were able to generate prevalence estimates for less frequently reported pregnancy complications (e.g., PPROM). In addition, while the pregnancy and neonatal outcomes are explicitly defined for the DELIVER study, the inclusion criteria included studies with varying definitions of each outcome given the paucity of data on some pregnancy outcomes and the known challenges of measuring these outcomes in low-resource settings. The inclusion of a range of definitions allowed us to generate overall prevalence estimates and conduct sensitivity analyses that were restricted to studies that defined outcomes most similarly to the DELIVER study protocol. Finally, two independent reviewers assessed each potential full-text manuscript for inclusion, and all the manuscripts and abstracted data included in the meta-analyses were read and reviewed by a second reviewer to guarantee quality.

There were several limitations to these meta-analyses. First, there was heterogeneity in the study design, study objectives, study inclusion/exclusion criteria affecting the risk-level of the included pregnant population, methods of ascertaining outcomes, the underlying health status of included pregnant individuals, and prevalence estimates across the included manuscripts. Our analysis also excluded studies restricted to adolescents and was unable to include age-standardization due to limitations in the available data. Collectively, our findings should be interpreted with this context in mind. Second, the pooled prevalence estimates for rare outcomes and subgroup analyses were limited by the paucity of data. The confidence intervals are wide and country-specific estimates may rely on data from only a few studies. For example, these meta-analyses suggest that women living with HIV had a higher pooled prevalence for many of the included outcomes such as stillbirth, preterm birth, congenital anomalies, and LBW. However, a minority of included studies reported prevalence by HIV status, and so these results, especially for the rare outcomes, should be interpreted cautiously. Third, despite the robust search, the review was limited by how manuscripts are indexed in MEDLINE. Manuscripts were frequently identified for inclusion in the review during the search for a single outcome while also including data on multiple additional outcomes of interest. Often, these manuscripts were not subsequently identified through specific searches for those other outcomes despite providing relevant estimates; thus, their inclusion for certain outcomes in this project occurred as a result of chance findings in manuscripts identified for another outcome. In addition, only one database was searched. Therefore, it is certain that additional prevalence estimates for these outcomes from Malawi, South Africa, Uganda, and Zimbabwe were not included in these meta-analyses. Fourth, the search strategy only included English language manuscripts identified through one database, which may have contributed to missing some prevalence estimates. Fifth, having one reviewer conduct initial data abstraction with a second reviewing for accuracy (instead of independent data abstraction by two reviewers) could have introduced bias. However, a rigorous process for identifying and adjudicating any errors and disagreements in data abstraction that were identified was followed limiting our concern for bias from this approach. Finally, while these literature reviews were conducted systematically, this review intentionally varied from certain aspects of the PRISMA guidelines for systematic reviews to best address our research question about the prevalence of pregnancy and neonatal outcomes and in response to the available data (33). For example, eligible countries were modified after the initial development of the searches. In addition, because of the size and scope of the literature reviews and the goal of estimating general population prevalence (versus intervention effect), we elected to present a qualitative summary of potential bias as this better represented the range of important considerations for interpretation of these data.

The prevalence estimates generated by this literature review and meta-analyses will be compared with the results of a records review of pregnancy outcomes at primary care and referral facilities affiliated with the DELIVER study (34). Together, these results will be utilized to assess whether the frequency of pregnancy and neonatal outcomes among pregnant women randomized to the dapivirine vaginal ring or oral TDF are similar to those observed in the areas where the study will be conducted. Importantly, these prevalence estimates will be a valuable resource for future trials of investigational products in pregnancy, including several HIV prevention methods (e.g., long-acting injectable cabotegravir) and maternal immunizations that are in the process of development or newly approved (35–37), including COVID-19 vaccines. In addition, these estimates may inform the allocation of resources and policies to prevent adverse pregnancy outcomes and complications. While this review identified a robust volume of data for some outcomes, data were severely lacking for other important pregnancy-related conditions, the quality of outcome ascertainment was variable, and stratification by HIV status was not ubiquitous. Fundamentally, there is an urgent need for pregnant people to be included in clinical research to understand the safety and efficacy of investigational products. There is also an urgent need to routinely collect quality and standardized data as the current, unreliable estimates make it challenging to distribute resources and understand whether quality improvement efforts are effective. Understanding the true burden of adverse pregnancy outcomes and complications in LMICs is essential to better serve women and other individuals of reproductive potential globally.

## Data Availability Statement

The original contributions presented in the study are included in the article/Supplementary Material. Further inquiries can be directed to the corresponding author/s.

## Author Contributions

JB, KB, LF, and BM conceived of this project. EL and AM conducted the systematic review and meta analysis under supervision by JB. EL, AM, and JB drafted the first version of the manuscript. EL, KB, LF, BM, RB, LN, and JB interpreted the results. All authors reviewed, commented on, and approved the final manuscript.

## Funding

The Microbicide Trials Network is funded by the National Institute of Allergy and Infectious Diseases (UM1AI068633, UM1AI068615, and UM1AI106707), with cofunding from the *Eunice Kennedy Shriver* National Institute of Child Health and Human Development and the National Institute of Mental Health, all components of the U.S. National Institutes of Health. EML was additionally funded by a National Institute of Child Health and Human Development fellowship (F32 HD100202). The content is solely the responsibility of the authors and does not necessarily represent the official views of the NIH. The funders had no role in study design, data collection and analysis, decision to publish, or preparation of the manuscript.

## Conflict of Interest

The authors declare that the research was conducted in the absence of any commercial or financial relationships that could be construed as a potential conflict of interest.

## Publisher's Note

All claims expressed in this article are solely those of the authors and do not necessarily represent those of their affiliated organizations, or those of the publisher, the editors and the reviewers. Any product that may be evaluated in this article, or claim that may be made by its manufacturer, is not guaranteed or endorsed by the publisher.
