# Supplementary material for: Pooled Prevalence of Adverse Pregnancy and Neonatal Outcomes in Malawi, South Africa, Uganda, and Zimbabwe: Results From a Systematic Review and Meta-Analyses to Inform Trials of Novel HIV Prevention Interventions During Pregnancy
Source: Front Reprod Health. Author manuscript; Available in PMC 2022 Feb 18. (PMC8856667; doi:10.3389/frph.2021.672446)
Supplement: Supplementary file 1 [file NIHMS1769644-supplement-Supplemetal_Materials_Lokken_et_al.pdf]

Supplementary File

Title: Pooled prevalence of adverse pregnancy and neonatal outcomes in Malawi, South Africa, Uganda, and Zimbabwe – Results from a systematic review and meta-analysis to inform trials of novel HIV prevention interventions during pregnancy

Authors: Erica M Lokken, Anya Mathur, Katherine E Bunge, Lee Fairlie, Bonus Makanani, Richard Beigi, Lisa Noguchi, Jennifer E Balkus

Table of Contents

SECTION A: SEARCH TERMS, RESULTS, AND INCLUDED STUDIES 7

Table A.1. Search Terms, Results, and Date for 10 literature reviews..... 7

Table A.2. Included References by Outcome 10

SECTION B: PREGNANCY LOSS & STILLBIRTH 17

Table B.1. Search Result Flow 17

Table B.2. Pooled Prevalence of Pregnancy Loss – Overall, by MTN-042 Country, and by HIV status 18

Figure B.1. Forest Plot Summarizing the Pooled Prevalence of Pregnancy Loss – Malawi 19

Figure B.2. Forest Plot Summarizing the Pooled Prevalence of Pregnancy Loss - South Africa 20

Figure B.3. Forest Plot Summarizing the Pooled Prevalence of Pregnancy Loss – Uganda 21

Figure B.4. Forest Plot Summarizing the Pooled Prevalence of Pregnancy Loss - Zimbabwe 22

Figure B.5. Forest Plot Summarizing the Pooled Prevalence of Pregnancy Loss Among Women Living with HIV 23

Figure B.6. Forest Plot Summarizing the Pooled Prevalence of Pregnancy Loss Among HIV Negative Women 24

Table B.3. Sensitivity Analysis Excluding Studies with Pregnancy Loss Definition Undefined: Pooled Prevalence of Pregnancy Loss – Overall, by MTN-042 Country, and by HIV status 25

Table B.4. Sensitivity Analysis Including Studies Defining Pregnancy Loss ≤20 weeks: Pooled Prevalence of Pregnancy Loss – Overall, by MTN-042 Country, and by HIV status 26

Table B.5. Sensitivity Analysis Including Studies Defining Pregnancy Loss ≤28 weeks: Pooled Prevalence of Pregnancy Loss – Overall, by MTN-042 Country, and by HIV status 27

Table B.6. Sensitivity Analysis Excluding Outliers: Pooled Prevalence of Pregnancy Loss – Overall, by MTN-042 Country, and by HIV status 28

Table B.7. Pooled Prevalence of Stillbirth– Overall, by MTN-042 Country, and by HIV status 29

Figure B.7. Forest Plot Summarizing Pooled Prevalence of Stillbirth - Malawi 30

Figure B.8. Forest Plot Summarizing Pooled Prevalence of Stillbirth - South Africa 31

Figure B.9. Forest Plot Summarizing the Pooled Prevalence of Stillbirth - Uganda 32

Figure B.10. Forest Plot Summarizing the Pooled Prevalence of Stillbirth - Zimbabwe 33

Figure B.11. Forest Plot Summarizing the Pooled Prevalence of Stillbirth among Women Living with HIV 34

Figure B.12. Forest Plot Summarizing the Pooled Prevalence of Stillbirth among HIV Negative Women 35

Table B.8. Sensitivity Analysis Excluding Studies with Stillbirth Definition Undefined: Pooled Prevalence of Stillbirth – Overall, by MTN-042 Country, and by HIV status....... 36

Table B.9. Pooled Prevalence of Antepartum Stillbirth – Overall and by MTN-042 Country 38

Table B.10. Pooled Prevalence of Intrapartum Stillbirth – Overall and by MTN-042 Country 39

Table B.11. Sensitivity Analysis Including Studies Defining Stillbirth ≥20 Weeks Gestation: Pooled Prevalence of Stillbirth – Overall and by MTN-042 Country............. 40

Table B.12. Sensitivity Analysis Including Studies Defining Stillbirth ≥28 weeks Gestation: Pooled Prevalence of Stillbirth – Overall and by MTN-042 Country............. 41

Table B.13. Sensitivity Analysis Excluding Outliers: Pooled Prevalence of Stillbirth – Overall, by MTN-042 Country, and by HIV status........ 42

Notes B.1. Pregnancy Loss and Stillbirth Review and Meta-Analyses: Study Specific Notes 43

Table B.14. Pregnancy Loss and Stillbirth Meta-Analyses Outliers 44

Table B.15. Pregnancy Loss Study Definition – Included Studies........ 46

Table B.16. Stillbirth Study Definition – Included Studies 49

SECTION C: PRETERM BIRTH 54

Table C.1. Search Result Flow........ 54

Table C.2. Pooled Prevalence of Preterm Birth – Overall, by MTN-042 Country, and by HIV status 55

Figure C.1. Forest Plot Summarizing the Pooled Prevalence of Preterm Birth – Malawi 56

Figure C.2. Forest Plot Summarizing the Pooled Prevalence of Preterm Birth – South Africa........ 57

Figure C.3. Forest Plot Summarizing the Pooled Prevalence of Preterm Birth – Uganda 58

Figure C.4. Forest Plot Summarizing the Pooled Prevalence of Preterm Birth – Zimbabwe 59

Figure C.5. Forest Plot Summarizing the Pooled Prevalence of Preterm Birth Among Women Living with HIV 60

Figure C.6. Forest Plot Summarizing the Pooled Prevalence of Preterm Birth Among HIV Negative Women........ 61

Table C.3. Sensitivity Analysis Live Birth Only: Pooled Prevalence of Preterm Birth – Overall, by MTN-042 Country, and by HIV Status 62

Table C.4. Sensitivity Analysis Excluding Studies with Preterm Birth Definition Undefined: Pooled Prevalence of Preterm Birth – Overall, by MTN-042 Country, and by HIV Status........ 63

SECTION D: CONGENITAL ANOMALIES ... *64*

Table D.1. Search Result Flow ... 64

Table D.2. Pooled Prevalence of Congenital Anomalies – Overall, by MTN-042 Country, and by HIV status ... 65

Figure D.1. Forest Plot Summarizing the Pooled Prevalence of Congenital Anomalies 66

Figure D.2. Forest Plot Summarizing the Pooled Prevalence of Congenital Anomalies Among Women Living with HIV ... 67

Table D.3. Sensitivity Analysis Excluding Outliers: Pooled Prevalence of Congenital Anomalies – Overall, by MTN-042 Country, and by HIV status ... 68

Table D.4. Sensitivity Analysis Excluding Non-Randomized Trial Data: Pooled Prevalence of Congenital Anomalies – Overall, by MTN-042 Country, and by HIV status ... 69

Figure D.3. Forest Plot Summarizing the Pooled Prevalence of Congenital Anomalies Among Women Living with HIV – Restricted to Studies Using Randomized Trial Data 70

Table D.5. Overall pooled prevalence of specific and system-specific congenital anomalies ... 71

Table D.6. Congenital Anomaly: Study Definition ... 73

Table D.7. Congenital Anomaly Meta-Analyses Outliers ... 78

SECTION E: LOW BIRTH WEIGHT ... *79*

Table E.1. Search Result Flow ... 79

Table E.2. Pooled Prevalence of Low Birth Weight – Overall, by MTN-042 Country, and by HIV status ... 80

Figure E.1. Forest Plot Summarizing the Pooled Prevalence of Low Birth Weight– Malawi ... 81

Figure E.2. Forest Plot Summarizing the Pooled Prevalence of Low Birth Weight - South Africa ... 82

Figure E.3. Forest Plot Summarizing the Pooled Prevalence of Low Birth Weight – Uganda ... 83

Figure E.4. Forest Plot Summarizing the Pooled Prevalence of Low Birth Weight – Zimbabwe ... 84

Figure E.5. Forest Plot Summarizing the Pooled Prevalence of Low Birth Weight - Among Women Living with HIV ... 85

Figure E.6. Forest Plot Summarizing the Pooled Prevalence of Low Birth Weight Among HIV Negative Women ... 86

Table E.3. Sensitivity Analysis Including Studies with Live Birth Only: Pooled Prevalence of Low Birth Weight – Overall, by MTN 042 Country, and by HIV Status 87

Table E.4. Sensitivity Analysis Excluding Outliers: Pooled Prevalence of Low Birthweight – Overall, by MTN-042 Country, and by HIV status ... 88

Table E.5. Low Birthweight Meta-Analysis Outliers ... 89

SECTION F: NEONATAL MORTALITY ... *90*

Table F.1. Search Result Flow 90

Table F.2. Pooled Prevalence of Neonatal Mortality – Overall, by MTN-042 Country, and by HIV status 91

Figure F.1. Forest Plot Summarizing the Pooled Prevalence of Neonatal Mortality – Overall and by Country 92

Figure F.2. Forest Plot Summarizing the Pooled Prevalence of Neonatal Mortality Among Women Living with HIV 93

Table F.3. Neonatal Mortality (<28 days) Summary - Sensitivity analysis excluding studies that did not define "neonatal mortality" 94

Notes F.1. Neonatal Mortality Study Specific Notes: 95

SECTION G: CHORIOAMNIONITIS 96

Table G.1. Search Result Flow 96

Table G.2. Pooled Prevalence of Chorioamnionitis – Overall, by MTN-042 Country, and by HIV status 97

Figure G.1. Forest Plot Summarizing the Pooled Prevalence of Chorioamnionitis 98

Notes G.1. Chorioamnionitis Study Specific Notes 99

SECTION H: ENDOMETRITIS 100

Table H.1. Search Result Flow 100

Table H.2. Pooled Prevalence of Endometritis – Overall, by MTN-042 Country, and by HIV status 101

Figure H.1. Forest Plot Summarizing the Pooled Prevalence of Endometritis 102

SECTION I: POSTPARTUM HEMORRHAGE 103

Table I.1. Search Result Flow 103

Table I.2. Pooled Prevalence of Postpartum Hemorrhage – Overall, by MTN-042 country, and by HIV status 104

Figure I.2. Forest Plot Summarizing the Pooled Prevalence of Postpartum Hemorrhage Among Women Living with HIV 106

Figure I.3. Forest Plot Summarizing the Pooled Prevalence of Postpartum Hemorrhage Among HIV Negative Women 107

Figure I.4. Forest Plot Summarizing the Pooled Prevalence of Postpartum Hemorrhage - ≥500mL Blood Loss 108

SECTION J: HYPERTENSIVE DISORDERS OF PREGNANCY 109

Table J.1. Search Result Flow 109

Table J.2. Pooled Prevalence of Gestational Hypertension – Overall, by MTN-042 Country and by HIV status 110

Figure J.1. Forest Plot Summarizing the Pooled Prevalence of Gestational Hypertension 111

Figure J.2. Forest Plot Summarizing the Pooled prevalence of Gestational Hypertension Among Women Living with HIV 112

Figure J.3. Forest Plot Summarizing the Pooled prevalence of Gestational Hypertension Among HIV Negative Women 113

PRE-ECLAMPSIA/ECLAMPSIA 114

Table J.3. Pooled Prevalence of Preeclampsia/Eclampsia – Overall, by MTN-042 Country, and by HIV status 114

Figure J.4. Forest Plot Summarizing the Pooled Prevalence of Pre-eclampsia/Eclampsia 115

Figure J.5. Forest Plot Summarizing the Pooled Prevalence of Pre-eclampsia/Eclampsia Among Women Living with HIV 116

Figure J.6. Forest Plot Summarizing the Pooled Prevalence of Pre-eclampsia/Eclampsia Among HIV Negative Women 117

SECTION K: PPROM 118

Table K.1. Search Result Flow 118

Table K.2. Pooled Prevalence of PPROM– Overall, by MTN-042 Country, and by HIV status 119

Figure K.1. Forest Plot Summarizing the Pooled Prevalence of PPROM 120

REFERENCES 121

SECTION A: SEARCH TERMS, RESULTS, AND INCLUDED STUDIES

Table A.1. Search Terms, Results, and Date for 10 literature reviews

Type of Outcome	Outcome	Terms*	Results†
Pregnancy outcome	Pregnancy Loss & Stillbirth	("pregnancy loss" OR stillbirth* OR "stillborn" OR "still birth" OR "still births" OR "still born" OR stillbirth[mesh] OR "fetal demise" OR "fetal death" OR fetal death [Mesh] OR "spontaneous abortion" OR abortion,spontaneous [mesh] OR "miscarriage") AND (Ethiopia OR Ethiopian OR Kenya OR Kenyan OR Tanzania OR tanzanian Or Uganda OR Ugandan OR Mozambique OR Mozambican OR Malawi OR Malawian OR Zimbabwe OR Zimbabwean OR Zambia OR Zambian OR Botswana OR Botswanan OR "South Africa" OR "South African" OR South Africa [Mesh] OR Lesotho OR Basotho OR Swaziland OR Swazi OR Namibia OR Namibian) AND (("1998/01/01"[PDat] : "2018/12/31"[PDat]) AND English[lang])	874
	Preterm birth	("premature labor"[All Fields] OR "preterm labour"[All Fields] OR "Preterm birth"[All Fields] OR "premature delivery"[All Fields] OR "preterm delivery"[All Fields] OR preterm[All Fields] OR "premature birth"[MESH]) AND (Ethiopia OR Ethiopian OR Kenya OR Kenyan OR Tanzania OR tanzanian Or Uganda OR Ugandan OR Mozambique OR Mozambican OR Malawi OR Malawian OR Zimbabwe OR Zimbabwean OR Zambia OR Zambian OR Botswana OR Botswanan OR "South Africa" OR "South African" OR South Africa [Mesh] OR Lesotho OR Basotho OR Swaziland OR Swazi OR Namibia OR Namibian) AND (("1998/01/01"[PDat] : "2018/12/31"[PDat]) AND English[lang])	590
Infant outcome	Congenital anomaly	("Congenital anomaly" OR "congenital abnormalities" OR "congenital abnormality" OR "congenital abnormalities" OR "birth defect" OR "Polydactyly" OR "cranio-facial" OR "cranio facial" OR "craniofacial" OR "neural tube defect" OR "neural tube" OR "spina bifida" OR "anencephaly" OR "heart defect" OR "inguinal hernia" OR "umbilical hernia" OR "micrognathia" OR "epicanthic folds" OR "microcephaly" OR "hydrocephaly" OR "cleft palate" OR "cleft lip") AND (Ethiopia OR Ethiopian OR Kenya OR Kenyan OR Tanzania OR tanzanian Or Uganda OR Ugandan OR Mozambique OR Mozambican OR Malawi OR Malawian OR Zimbabwe OR Zimbabwean OR Zambia OR Zambian OR Botswana OR Botswanan OR "South Africa" OR "South African" OR South Africa [Mesh] OR Lesotho OR Basotho OR Swaziland OR Swazi OR Namibia OR Namibian) AND (("1998/01/01"[PDat] : "2018/12/31"[PDat]) AND English[lang])	620

	**Low birth weight**	("low birth weight" OR "lbw" OR "low birthweight" OR infant, low birth weight[mesh]) AND (Ethiopia OR Ethiopian OR Kenya OR Kenyan OR Tanzania OR tanzanian Or Uganda OR Ugandan OR Mozambique OR Mozambican OR Malawi OR Malawian OR Zimbabwe OR Zimbabwean OR Zambia OR Zambian OR Botswana OR Botswanan OR "South Africa" OR "South African" OR South Africa [Mesh] OR Lesotho OR Basotho OR Swaziland OR Swazi OR Namibia OR Namibian) AND (("1998/01/01"[PDat] : "2018/12/31"[PDat]) AND English[lang])	**791**
	**Neonatal Mortality**	("neonatal death" OR perinatal death[mesh] OR perinatal mortality[mesh] OR "neonatal mortality" OR "perinatal mortality") AND (Ethiopia OR Ethiopian OR Kenya OR Kenyan OR Tanzania OR tanzanian Or Uganda OR Ugandan OR Mozambique OR Mozambican OR Malawi OR Malawian OR Zimbabwe OR Zimbabwean OR Zambia OR Zambian OR Botswana OR Botswanan OR "South Africa" OR "South African" OR South Africa [Mesh] OR Lesotho OR Basotho OR Swaziland OR Swazi OR Namibia OR Namibian) AND (("1998/01/01"[PDAT] : "2018/12/31"[PDAT]) AND English[lang])	**834**
**Pregnancy complication**	**Chorioamnionitis**	(choriomanionitis[mesh] OR "choriomanionitis" OR "intraamniotic infection" OR "intra-amniotic infection" OR "amnionitis" OR "funisitis") AND (Ethiopia OR Ethiopian OR Kenya OR Kenyan OR Tanzania OR tanzanian Or Uganda OR Ugandan OR Mozambique OR Mozambican OR Malawi OR Malawian OR Zimbabwe OR Zimbabwean OR Zambia OR Zambian OR Botswana OR Botswanan OR "South Africa" OR "South African" OR South Africa [Mesh] OR Lesotho OR Basotho OR Swaziland OR Swazi OR Namibia OR Namibian) AND (("1998/01/01"[PDat] : "2018/12/31"[PDat]) AND English[lang])	**6**
	**Endometritis**	("endometritis"[MeSH Terms] OR "peripartum endometritis" OR "Endometritis"[All Fields] OR "postpartum endometritis"[All Fields] AND (Ethiopia OR Ethiopian OR Kenya OR Kenyan OR Tanzania OR tanzanian Or Uganda OR Ugandan OR Mozambique OR Mozambican OR Malawi OR Malawian OR Zimbabwe OR Zimbabwean OR Zambia OR Zambian OR Botswana OR Botswanan OR "South Africa" OR "South African" OR South Africa [Mesh] OR Lesotho OR Basotho OR Swaziland OR Swazi OR Namibia OR Namibian) AND (("1998/01/01"[PDAT] : "2018/12/31"[PDAT]) AND English[lang])	**43**
	**Postpartum Hemorrhage**	("peripartum hemorrhage" OR "peripartum haemorrhage" OR postpartum hemorrhage [mesh] OR "postpartum haemorrhage" OR "postpartum hemorrhage") AND (Ethiopia OR Ethiopian OR Kenya OR Kenyan OR Tanzania OR tanzanian Or Uganda OR Ugandan OR Mozambique OR Mozambican OR Malawi OR Malawian OR Zimbabwe OR Zimbabwean OR Zambia OR Zambian OR Botswana OR Botswanan OR "South Africa" OR "South African" OR South Africa	**217**

		[Mesh] OR Lesotho OR Basotho OR Swaziland OR Swazi OR Namibia OR Namibian) AND (("1998/01/01"[PDAT] : "2018/12/31"[PDAT]) AND English[lang])	
	**Hypertensive disorders of pregnancy**	("preeclampsia" OR preeclampsia [mesh] OR "eclampsia" OR "gestational hypertension" OR "pregnancy induced hypertension" OR "HELLP" OR "preeclampsia-eclampsia" OR "pre-eclampsia") AND (Ethiopia OR Ethiopian OR Kenya OR Kenyan OR Tanzania OR tanzanian Or Uganda OR Ugandan OR Mozambique OR Mozambican OR Malawi OR Malawian OR Zimbabwe OR Zimbabwean OR Zambia OR Zambian OR Botswana OR Botswanan OR "South Africa" OR "South African" OR South Africa [Mesh] OR Lesotho OR Basotho OR Swaziland OR Swazi OR Namibia OR Namibian) AND (("1998/01/01"[PDAT] : "2018/12/31"[PDAT]) AND English[lang])	**549**
	**PPROM**	("preterm premature rupture membranes" OR "PPROM" OR Fetal Membranes, Premature Rupture [mesh]) AND (Ethiopia OR Ethiopian OR Kenya OR Kenyan OR Tanzania OR tanzanian Or Uganda OR Ugandan OR Mozambique OR Mozambican OR Malawi OR Malawian OR Zimbabwe OR Zimbabwean OR Zambia OR Zambian OR Botswana OR Botswanan OR "South Africa" OR "South African" OR South Africa [Mesh] OR Lesotho OR Basotho OR Swaziland OR Swazi OR Namibia OR Namibian) AND (("1998/01/01"[PDAT] : "2018/12/31"[PDAT]) AND English[lang])	**50**

[*] Swaziland was renamed eSwatini in 2018. Because these literature review searches were conducted in July 2018 and aimed to include data published between 1998 and the time of the search, the search strategy utilized “Swaziland”.

[†] All searches were conducted on 11 July 2018, except the Pregnancy Loss & Stillbirth search which was conducted on 2 July 2018.

Table A.2. Included References by Outcome

Author, Year (Reference)	Country	HIV status*	Outcome											
			Pregnancy Loss	Stillbirth	Congenital Anomaly	LBW	Preterm Birth	Neonatal Mortality	Chorioamnionitis	Endometritis	Postpartum Hemorrhage	Gestational Hypertension	Preeclampsia/ Eclampsia	PPROM
Abdel-Aleem, 2010 (1)	South Africa	NR									X			
Ades, 2013 (2)	Uganda	Positive			X			X						
Ali, 2017 (3)	Malawi	NR	X	X	X			X						
Allanson, 2015 (4)	South Africa	Both		X										
Anderson, 2018 (5)	South Africa	Both				X	X							
Arinaitwe, 2013 (6)	Uganda	Negative		X		X								
Ashorn, 2015 (7)	Malawi	Both	X	X		X	X	X						
Asiki, 2015 (8)	Uganda	Both		X										
Atukunda, 2014 (9)	Uganda	NR									X			
Bailey, 2017 (10)	Malawi	Both		X										
Bamigboye, 1998 (11)	South Africa	NR									X			
Basu, 2010 (12)	South Africa	NR		X			X					X		
Bebell, 2017 (13)	Uganda	Both								X				
Bello, 2010 (14)	South Africa	NR	X	X	X									
Beltman, 2011 (15)	Malawi	NR									X			
Bera, 2010 (16)	South Africa	Positive	X	X	X									
Bloch, 2015 (17)	South Africa	Both					X				X			
Bodkin, 2006 (18)	South Africa	Both					X					X		
Brahmbhatt, 2008 (19)	Uganda	Positive				X	X							
Braun, 2015 (20)	Uganda	Both		X		X	X							
Brittain, 2015 (21)	South Africa	NR	X				X							
Byaruhanga, 2000 (22)	Uganda	NR		X										
Chagomerana, 2017 (23)	Malawi	Positive					X							

Author, Year (Reference)	Country	HIV status*	Outcome											
			Pregnancy Loss	Stillbirth	Congenital Anomaly	LBW	Preterm Birth	Neonatal Mortality	Chorioamnionitis	Endometritis	Postpartum Hemorrhage	Gestational Hypertension	Preeclampsia/ Eclampsia	PPROM
Chetty, 2018 (24)	South Africa	Positive				X	X							
Chevalier, 2017 (25)	Malawi	Positive		X				X		X	X		X	
Chihana, 2015 (26)	Malawi	Both		X				X						
Colbourn, 2013 (27)	Malawi	NR		X				X						
Coutsoudis, 1999 (28)	South Africa	Positive	X	X		X	X	X						
Cutland, 2009 (29)	South Africa	Both		X			X			X				
Dow, 2013 (30)	Malawi	Positive				X	X							
Dreise, 2011 (31)	Uganda	NR			X									
Ebrahim, 2000 (32)	South Africa	NR		X										
Ellison, 2000 (33)	South Africa	NR										X		
Fall, 2015 (34)	South Africa	NR				X	X							
Fatti, 2016 (35)	South Africa	Positive		X										
Feng, 2010 (36)	Malawi	NR				X								
Feresu, 2003 (37)	Zimbabwe	NR				X	X							
Feresu, 2004 (38)	Zimbabwe	NR				X	X							
Feresu, 2005 (39)	Zimbabwe	NR		X										
Feresu, 2015 (40)	Zimbabwe	NR				X	X					X		
Filler, 2006 (41)	Malawi	Both						X						
Frank, 2004 (42)	South Africa	Both										X	X	
Friis, 2004 (43)	Zimbabwe	Both		X		X	X							
Friis, 2009 (44)	Zimbabwe	Both				X	X							
Gebhardt, 2009 (45)	South Africa	Negative	X	X			X					X	X	X
Gessessew, 2007 (46)	Ethiopia	NR												X
Gibb, 2012 (47)	Uganda	Positive		X	X		X							
Govender, 2017 (48)	South Africa	NR		X										
Gray, 2001(49)	Uganda	Both	X	X			X		X		X			

Author, Year (Reference)	Country	HIV status*	Outcome											
			Pregnancy Loss	Stillbirth	Congenital Anomaly	LBW	Preterm Birth	Neonatal Mortality	Chorioamnionitis	Endometritis	Postpartum Hemorrhage	Gestational Hypertension	Preeclampsia/ Eclampsia	PPROM
Gülmezoglu, 2001 (50)	South Africa	NR									X			
Gumede, 2017 (51)	South Africa	Both					X							
Gutman, 2013 (52)	Malawi	Negative		X	X	X	X							
Hall, 2014 (53)	South Africa	Both					X					X		
Harrison, 2007 (54)	South Africa	Both	X		X			X				X		
Hjertholm, 2018 (55)	Malawi	NR				X								
Hofmeyr, 1998 (56)	South Africa	NR									X			
Hussain, 2011 (57)	South Africa	Both		X		X	X							
Immink, 2008 (58)	South Africa	NR											X	
Kalanda, 2006 (59)	Malawi	Both				X	X							
Kalilani, 2010 (60)	Malawi	NR				X								
Kalumbi, 2001 (61)	Malawi	NR		X										
Kananura, 2016 (62)	Uganda	NR		X				X						
Kananura, 2017 (63)	Uganda	NR		X				X						
Kapisi, 2017 (64)	Uganda	Negative				X	X							
Kasumba, 2000 (65)	Uganda	NR		X		X								
Kaye, 2001 (66)	Uganda	NR												X
Kaye, 2006 (67)	Uganda	Both		X		X	X							
Kesande, 2014 (68)	Uganda	NR			X									
Kimani, 2016 (69)	Malawi, Uganda	NR	X	X		X	X							
Kiondo, 2014 (70)	Uganda	Both	X	X		X	X					X	X	
Kujala, 2017 (71)	Uganda	NR		X				X						
Kulmala, 2000 (72)	Malawi	Both	X	X		X		X						
Kumwenda, 2002 (73)	Malawi	Positive	X	X										
Kundodyiwa, 2001 (74)	Zimbabwe	NR									X			

Author, Year (Reference)	Country	HIV status*	Outcome											
			Pregnancy Loss	Stillbirth	Congenital Anomaly	LBW	Preterm Birth	Neonatal Mortality	Chorioamnionitis	Endometritis	Postpartum Hemorrhage	Gestational Hypertension	Preeclampsia/ Eclampsia	PPROM
Lavin, 2016 (75)	South Africa	NR		X										
Lewycka, 2013 (76)	Malawi	NR		X				X						
Liu, 2014 (77)	South Africa	Positive	X	X	X	X	X	X						
Luntamo, 2010 (78)	Malawi	Both				X	X							
Madhi, 2016 (79)	South Africa	Negative		X		X	X							
Majoko, 2002 (80)	Zimbabwe	NR										X		
Majoko, 2007 (81)	Zimbabwe	Both	X	X		X	X	X						
Makanani, 2018 (82)	Malawi, South Africa, Zimbabwe, Uganda	Negative	X	X	X									
Malaba, 2017 (83)	South Africa	Both	X	X		X	X							
McDonald, 2018 (84)	Uganda	Positive		X										
Metaferia, 2009 (85)	Malawi	NR		X										
Moodley, 2016a (86)	South Africa	Both		X		X	X							
Moodley, 2016b (87)	South Africa	Both										X	X	
Moodley, 2017 (88)	South Africa	Both	X	X			X	X						
Moyer, 2016 (89)	Uganda	Both		X		X	X							
Msamati, 2000 (90)	Malawi	NR			X									
Murray, 2018 (91)	South Africa	Both				X	X					X		
Musana, 2009 (92)	Kenya	Both							X					X
Muti, 2015 (93)	Zimbabwe	NR		X		X	X					X	X	
Mwanyumba, 2003 (94)	Kenya	NR							X	X				
Msyamboza, 2009 (95)	Malawi	NR		X		X								
Nabiwemba, 2013 (96)	Uganda	NR				X	X							

Author, Year (Reference)	Country	HIV status*	Outcome											
			Pregnancy Loss	Stillbirth	Congenital Anomaly	LBW	Preterm Birth	Neonatal Mortality	Chorioamnionitis	Endometritis	Postpartum Hemorrhage	Gestational Hypertension	Preeclampsia/ Eclampsia	PPROM
Naidoo, 2011 (97)	South Africa	NR	X	X										
Naidu, 2001 (98)	South Africa	Both		X				X						
Nakubulwa, 2017 (99)	Uganda	Both				X	X			X				X
Nankabirwa, 2011 (100)	Uganda	NR		X										
Nankabirwa, 2015 (101)	Uganda	Both		X				X						
Ndibazza, 2010 (102)	Uganda	Both	X	X		X								
Ndibazza, 2011 (103)	Uganda	Both			X									
Ndirangu, 2012 (104)	South Africa	Both				X	X	X						
Ndyomugyenyi, 2000 (105)	Uganda	NR	X	X		X								
Ndyomugyenyi, 2001 (106)	Uganda	NR		X		X								
Ndyomugyenyi, 2008 (107)	Uganda	NR		X	X	X	X	X						
Ndyomugyenyi, 2011 (108)	Uganda	NR	X	X	X	X	X	X						
Ngwenya, 2016 (109)	Zimbabwe	NR									X			
Ngwenya, 2017 (110)	Zimbabwe	NR											X	
Njiru, 2013 (111)	Uganda	NR				X								
Nkhoma, 2012 (112)	Malawi	Both				X								
Noble, 2005 (113)	Zimbabwe	Both					X							
Ntuli, 2012 (114)	South Africa	NR		X										
Ogbonna, 2007 (115)	Zimbabwe	Both				X								
Ononge, 2016 (116)	Uganda	Both					X				X			
Pattinson, 2003 (117)	South Africa	NR		X				X						
Pattinson, 2010 (118)	South Africa	Both				X		X			X		X	
Rempis, 2017 (119)	Uganda	Both		X										
Roh, 2017 (120)	Uganda	Positive				X	X							
Röllin, 2015 (121)	South Africa	NR				X	X							
Rollins, 2007 (122)	South Africa	Both	X	X		X								

Author, Year (Reference)	Country	HIV status*	Outcome											
			Pregnancy Loss	Stillbirth	Congenital Anomaly	LBW	Preterm Birth	Neonatal Mortality	Chorioamnionitis	Endometritis	Postpartum Hemorrhage	Gestational Hypertension	Preeclampsia/ Eclampsia	PPROM
Sania, 2017 (123)	South Africa	Positive				X								
Sayed, 2008 (124)	South Africa	NR			X									
Schrag, 2012 (125)	South Africa	Both				X		X				X		
Sebitloane, 2008 (126)	South Africa	Positive								X	X			
Sebitloane, 2017 (127)	South Africa	Both		X			X				X			
Shabalala, 2017 (128)	South Africa	Both									X			
Shamu, 2018 (129)	Zimbabwe	Both				X	X							
Sibeko, 2011 (130)	South Africa	Negative	X	X	X		X				X			
Sirak, 2014 (131)	Ethiopia	NR												X
Steyn, 2006 (132)	South Africa	NR				X	X							
Sullivan, 1999 (133)	Malawi	NR					X							
Taha, 2018 (134)	Malawi, South Africa, Zimbabwe, Uganda	Positive	X	X	X	X	X							
Talip, 2010 (135)	South Africa	NR	X	X				X						
Taylor, 2017 (136)	Malawi	NR				X	X							
Theron, 2015 (137)	South Africa	NR			X									
Ticconi, 2003 (138)t	Zimbabwe	Both	X	X										
Ticconi, 2005 (139)	Zimbabwe	Both				X	X							
Turner, 2013 (140)	Malawi	Positive				X	X		X					
Turton, 2017 (141)	South Africa	NR				X	X							
Van Den Broek, 2005 (142)	Malawi	NR	X	X		X	X							
Van Den Broek, 2009 (143)	Malawi	Both					X							
Van Den Broek, 2003 (144)	Malawi	NR		X										

Author, Year (Reference)	Country	HIV status[*]	Outcome											
			Pregnancy Loss	Stillbirth	Congenital Anomaly	LBW	Preterm Birth	Neonatal Mortality	Chorioamnionitis	Endometritis	Postpartum Hemorrhage	Gestational Hypertension	Preeclampsia/ Eclampsia	PPROM
Van Der Merwe, 2011 (145)	South Africa	Positive				X	X							
Verhoeff, 1999 (146)	Malawi	Both		X		X								
Villar, 2014 (147)	Kenya	NR												X
Wabwire-Mangen, 1999 (148)	Uganda	Positive							X					
Walter, 2006 (149)	Zambia	Positive							X					
Wanyama, 2016 (150)	Uganda	Negative				X								
Worku, 2014 (151)	Ethiopia	Both								X				
Young, 2012 (152)	Uganda	Positive	X	X										

[*] NR = not reported. Both = Includes both women living with HIV and HIV negative women. In some, but not all of these studies, prevalence was disaggregated by HIV serostatus.

SECTION B: PREGNANCY LOSS & STILLBIRTH

Table B.1. Search Result Flow

	N
**Title Review**	874
**Abstract Review**	237
**Manuscripts Reviewed**[*]	174
**Manuscripts Included From Main Search**[†]	124
**Manuscripts Added From Other Searches**[‡]	73
**Total Manuscripts Abstracted**	197
**Total Manuscripts Included For Analysis - 042 Countries Only**[§]	78
**Stillbirth**[**]	71
**Pregnancy Loss**[**]	29

[*] Does not include number of references from systematic reviews reviewed.
[†] Includes systematic review reference reviews.
[‡] Some of these include duplicate populations with the studies that were abstracted through the main search. These were removed at analysis.
[§] Excludes duplicate populations, studies with incorrect definitions of the outcomes
[**] Kimani_2016(69) reported prevalence estimates for two study countries and Taha_2018 (134) reported estimates for four countries. These studies were counted as one manuscript each but provided multiple prevalence estimates in the analysis phase.

Table B.2. Pooled Prevalence of Pregnancy Loss – Overall, by MTN-042 Country, and by HIV status

	# of estimates	# of pregnancies/ infants	Mean (Min, Max)	Median (IQR)	Pooled Prevalence (95%CI)	I^2 (p-value)
**Study Countries**	33	49,095	3.3% (0.0, 24.8)	1.6% (0.7, 2.2)	1.9% (1.1, 2.8)	97.2% (<0.01)
**Malawi**	7	3,956	1.0% (0.2, 1.8)	0.9% (0.2, 1.8)	0.6% (0.2, 1.2)	59.5% (0.02)
**South Africa**	14	30,080	4.2% (0.1, 20.8)	1.6% (1.2, 6.7)	2.5% (1.1, 4.3)	98.3% (<0.01)
**Uganda**	8	7,382	1.4% (0.0, 2.5)	1.4% (0.6, 2.2)	1.4% (0.7, 2.1)	75.3% (<0.01)
**Zimbabwe**	3	7,520	1.8% (0.3, 3.1)	2.2% (0.3, 3.1)	1.0% (0.0, 3.8)	-
**Multiple countries**(82)	1	157	24.8% -	24.8% -	24.8% (18.3, 32.4)	-
**Living with HIV**	11	5,255	2.1% (0.0, 9.1)	1.6% (0.7, 2.0)	0.8% (0.3, 1.5)	63.7% (<0.01)
**HIV Negative**	4	2,161	6.7% (0.3, 20.8)	2.9% (0.9, 12.6)	3.6% (0.5, 9.1)	94.6% (<0.01)

Figure B.1. Forest Plot Summarizing the Pooled Prevalence of Pregnancy Loss – Malawi

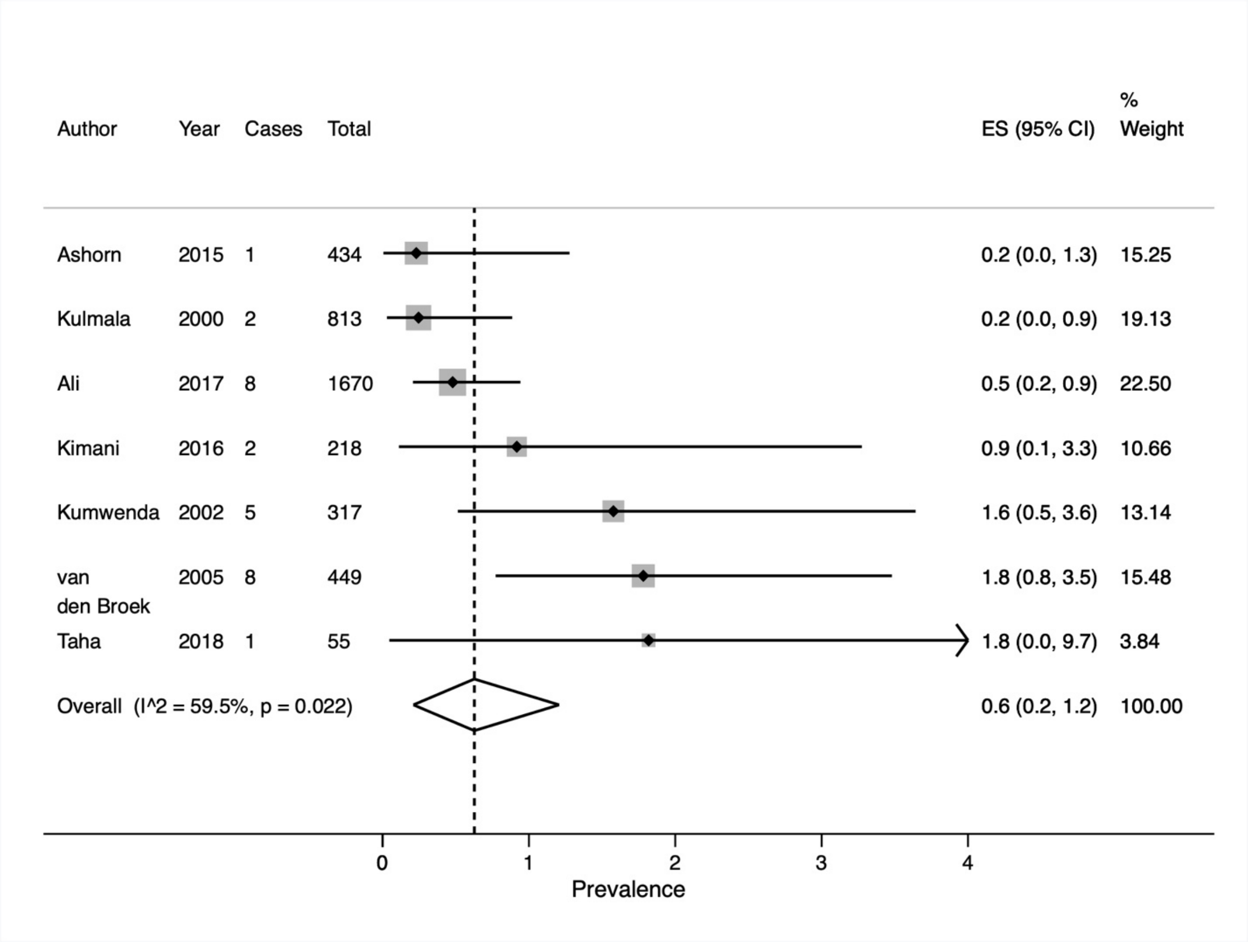

Figure B.2. Forest Plot Summarizing the Pooled Prevalence of Pregnancy Loss - South Africa

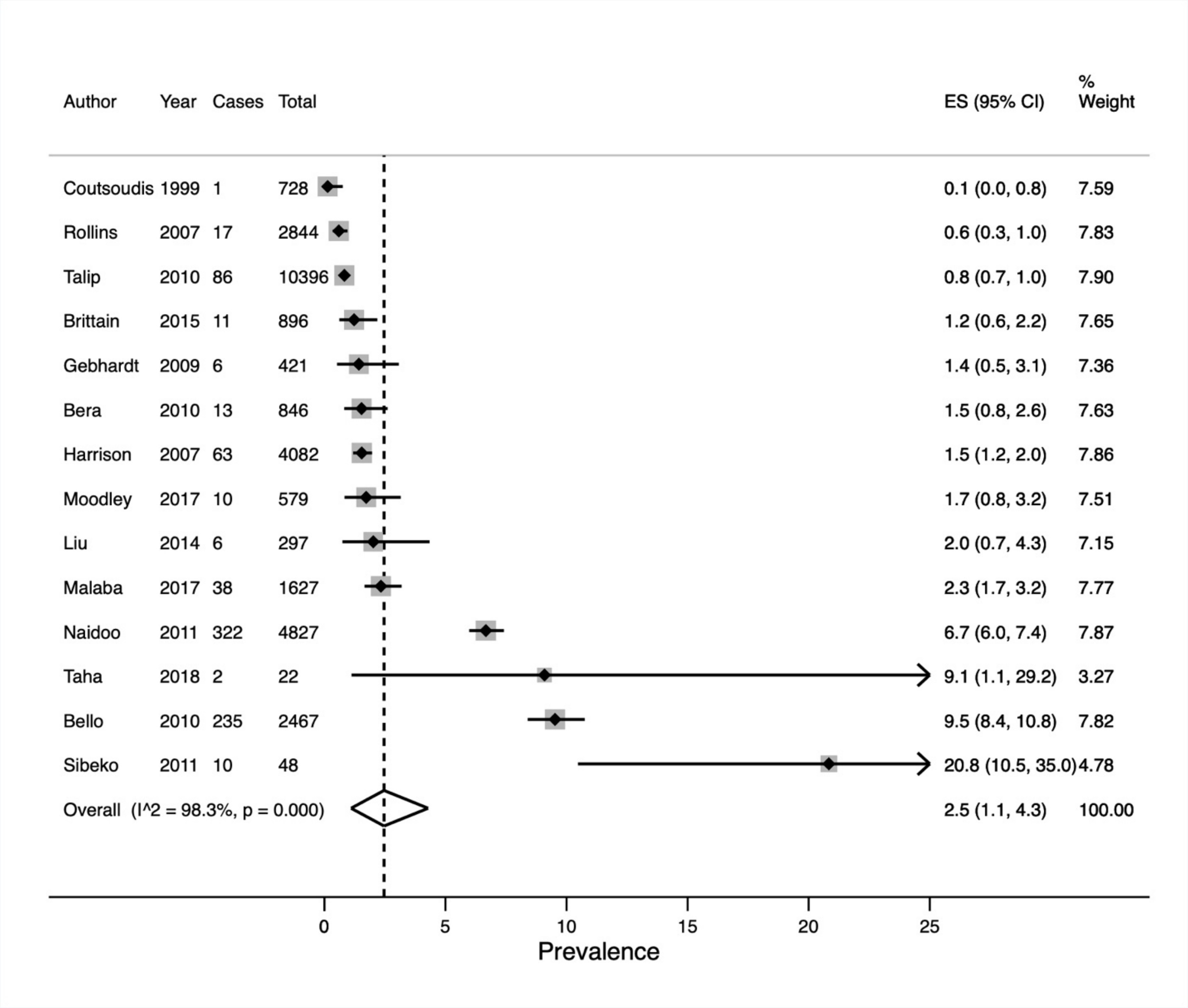

Figure B.3. Forest Plot Summarizing the Pooled Prevalence of Pregnancy Loss – Uganda

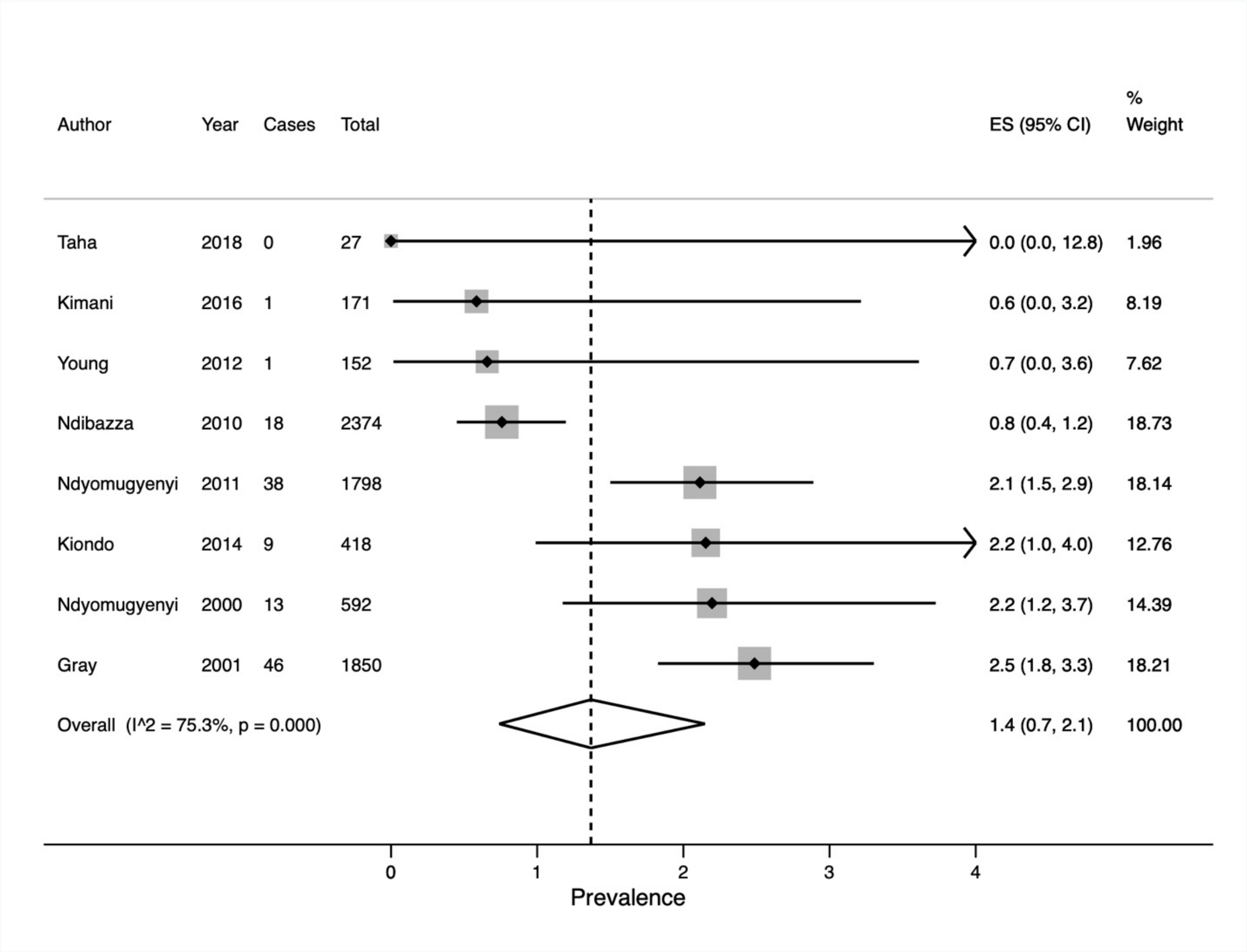

Figure B.4. Forest Plot Summarizing the Pooled Prevalence of Pregnancy Loss - Zimbabwe

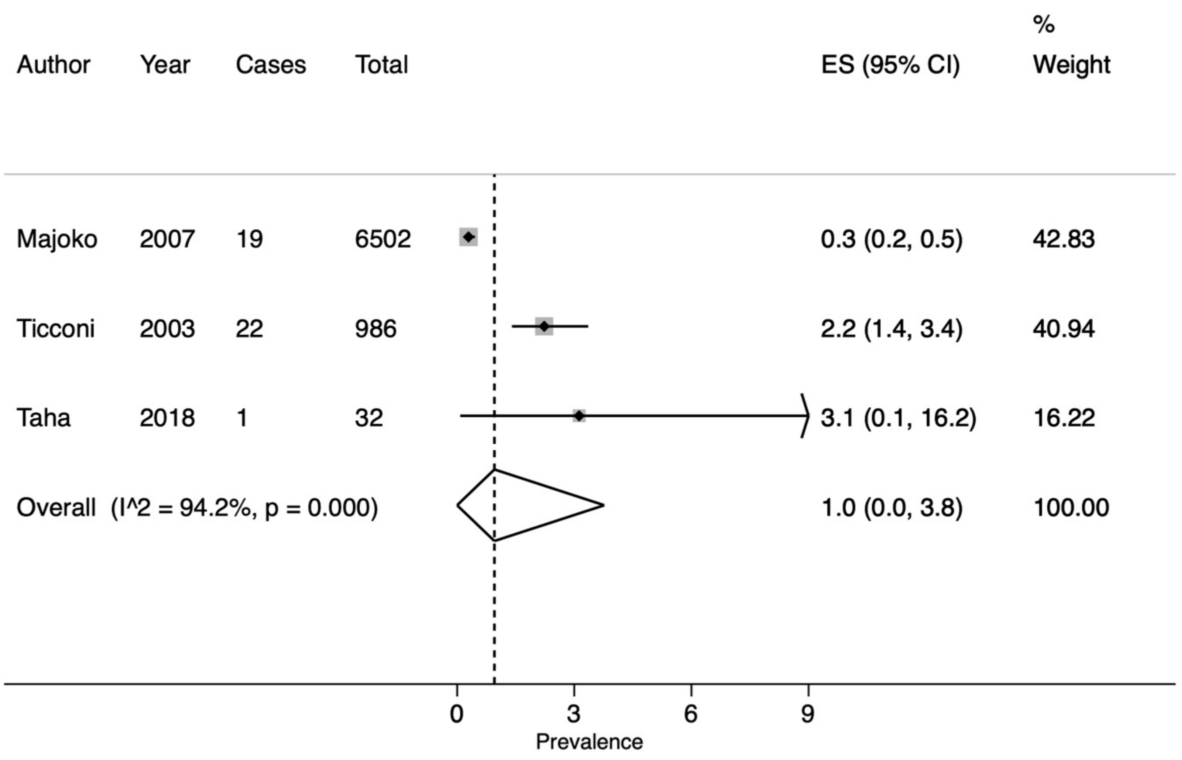

Figure B.5. Forest Plot Summarizing the Pooled Prevalence of Pregnancy Loss Among Women Living with HIV

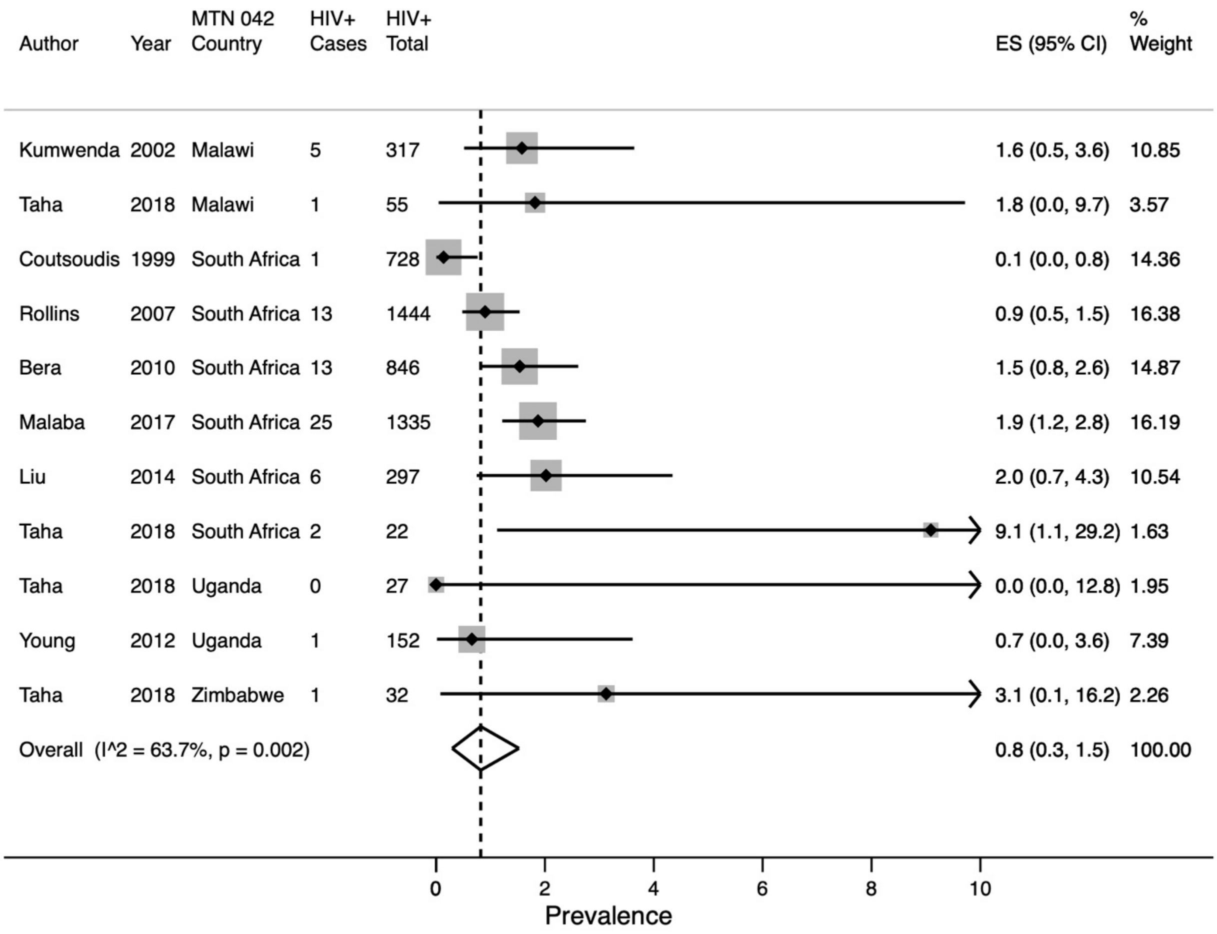

Figure B.6. Forest Plot Summarizing the Pooled Prevalence of Pregnancy Loss Among HIV Negative Women

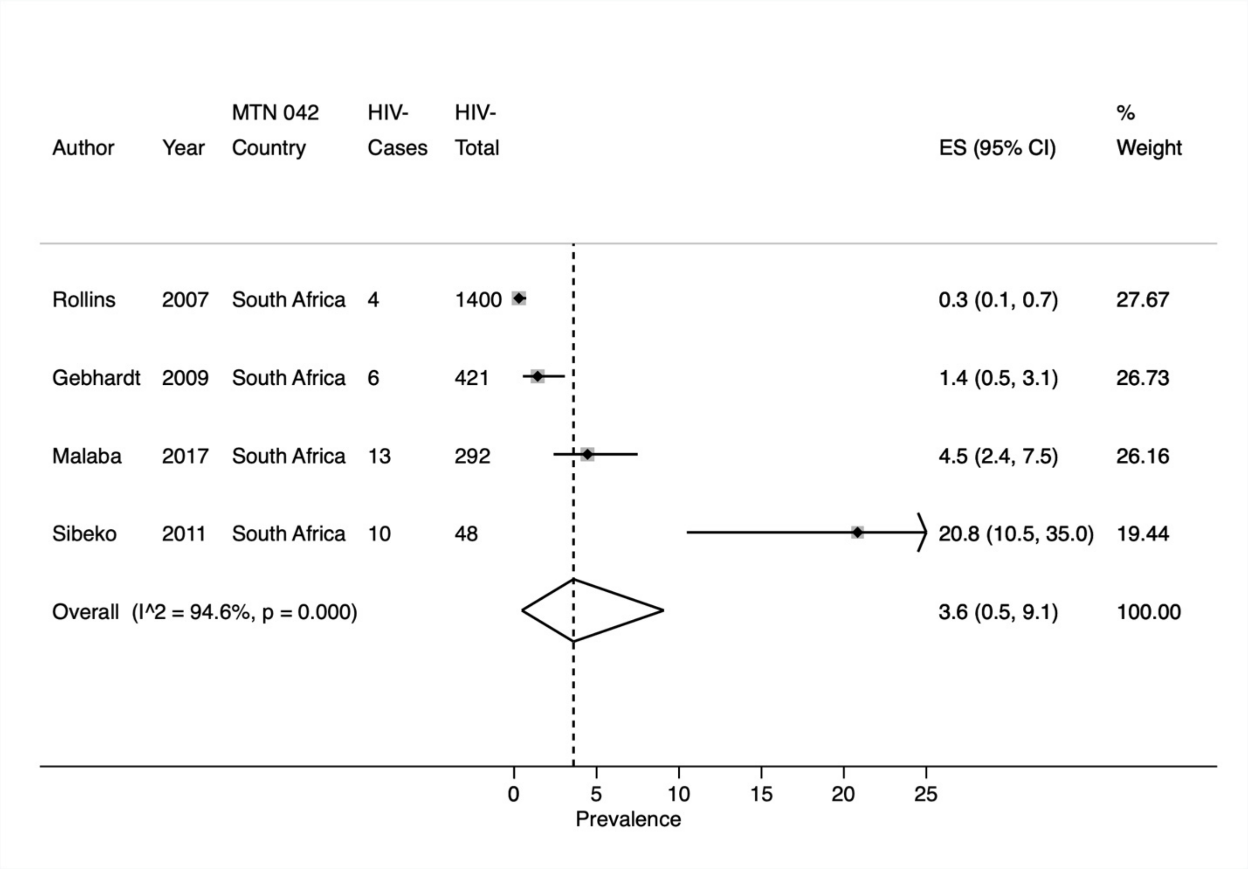

Table B.3. Sensitivity Analysis Excluding Studies with Pregnancy Loss Definition Undefined: Pooled Prevalence of Pregnancy Loss – Overall, by MTN-042 Country, and by HIV status

	# of estimates*	# of pregnancies/ infants	Mean (Min, Max)	Median (IQR)	Pooled Prevalence (95%CI)	I^2 (p-value)
**Study Countries**	25	32,780	3.0% (0.0, 24.8)	1.5% (0.6, 2.2)	1.7% (0.8, 3.0)	97.7% (<0.01)
**Malawi**	6	3,639	0.9% (0.2, 1.8)	0.7% (0.2, 1.8)	0.5% (0.1, 1.1)	56.2% (<0.01)
**South Africa**	11	18,740	3.3% (0.1, 9.5)	1.7% (1.4, 6.7)	2.3% (0.8, 4.4)	98.2% (<0.01)
**Uganda**	4	2,724	0.5% (0.0, 0.8)	0.6% (0.3, 0.7)	0.3% (0.1, 0.6)	0%
**Zimbabwe**	3	7,520	1.9% (0.3, 3.1)	2.2% (0.3, 3.1)	1.0% (0.0, 3.8)	-
**Multiple countries** (82)	1	157	24.8% -	24.8% -	24.8% (18.3, 32.4)	-
**Living with HIV**	10	4,938	2.1% (0.0, 9.1)	1.7% (0.7, 2.0)	0.8% (0.2, 1.5)	66.7% (<0.01)
**HIV negative**	3	2,113	2.1% (0.3, 4.4)	1.4% (0.3, 4.4%)	1.6% (0.1, 4.7)	92.6% (<0.01)

* Included Studies: Ali_2017 (3), Ashorn_2015 (7), Bello_2010 (14), Bera_2014 (16), Coutsoudis_1999 (8), Gebhardt_2009 (45), Harrison_2007 (54), Kimani_2016 (69), Kulmala_2000 (72), Liu_2014 (77), Majoko_2007 (81), Makanani_2018 (82), Malaba_2017 (83), Moodley_2017 (88), Nadoo_2011 (97), Ndibazza_2010 (102), Rollins_2007 (122), Taha_2018 (134), Ticconi_2003 (138), Young_2012 (152), van den Broek_2005 (142)

Table B.4. Sensitivity Analysis Including Studies Defining Pregnancy Loss ≤20 weeks: Pooled Prevalence of Pregnancy Loss – Overall, by MTN-042 Country, and by HIV status

	# of estimates*	# of pregnancies/ infants	Mean (Min, Max)	Median (IQR)	Pooled Prevalence (95% CI)	I^2 (p-value)
**Study Countries**	8	9,446	2.2% (0.0, 9.1)	1.2% (0.4, 2.7)	0.5% (0.0, 1.6)	83.9% (<0.01)
**Malawi**	2	1,725	1.1% (0.5, 1.8)	1.1% (0.5, 1.8)	0.2% (0.0, 0.6)	-
**South Africa**	1	22	9.1% -	9.1% -	9.1% (1.1,29.2)	-
**Uganda**	2	179	0.3% (0.0, 0.7)	0.3% (0.0, 0.7)	0.2% (0.0,1.9)	-
**Zimbabwe**	3	7,520	1.9% (0.3, 3.1)	2.2% (0.3, 3.1)	1.0% (0.0, 3.8)	-
**Multiple countries**	0	-	-	-	-	-
**Living with HIV**	5	288	2.9% (0.0, 9.1)	1.8% (0.7, 3.1)	1.2% (0.0, 3.9)	27.2% (0.2)
**HIV Negative**	0	-	-	-	-	-

[*] Includes studies defining miscarriage as those occurring between 12-20 weeks or ≤20 weeks. Included studies: Ali_2017 (3), Taha_2018 (134), Young_2012 (152), Majoko_2007 (81), Ticconi_2003 (138).

Table B.5. Sensitivity Analysis Including Studies Defining Pregnancy Loss ≤28 weeks: Pooled Prevalence of Pregnancy Loss – Overall, by MTN-042 Country, and by HIV status

	# of estimates*	# of pregnancies/ infants	Mean (Min, Max)	Median (IQR)	Pooled Prevalence (95% CI)	I^2 (p-value)
**Study Countries**	7	11,950	2.0% (0.1, 6.7)	1.5% (0.6, 2.3)	1.7% (0.4, 3.8)	97.6% (<0.01)
**Malawi**	1	218	0.9% -	-	0.9% (0.1, 3.3)	-
**South Africa**	5	11,561	2.5% (0.1, 6.7)	2.2% (1.5, 2.3)	2.1% (0.4, 4.9)	98.3% (<0.01)
**Uganda**	1	171	0.6% -	-	0.6% (0.0, 3.2)	-
**Zimbabwe**	0	-	-	-	-	-
**Living with HIV**	3	2,360	1.3% (0.1, 2.0)	1.9% (0.1, 2.0)	1.1% (0.1, 3.0)	89.2% (<0.01)
**HIV negative**	1	292	4.4% -	-	4.5% (2.4, 7.5)	-

* Does not includes studies defining miscarriage as those occurring between 12-20 weeks or <20 weeks. Included studies: Kimani_2016 (69), Coutsoudis_1999 (28), Harrison_2007 (54), Liu_2014 (77), Malaba_2017 (83), Naidoo_2011(97)

Table B.6. Sensitivity Analysis Excluding Outliers: Pooled Prevalence of Pregnancy Loss – Overall, by MTN-042 Country, and by HIV status

	# of estimates*	# of pregnancies/ infants	Mean (Min, Max)	Median (IQR)	Pooled Prevalence (95% CI)	I^2 (p-value)
**Study Countries**	28	41,574	1.3% (0, 3.1)	1.5% (0.6, 2.1)		86.2% (<0.01)
**Malawi**	7	3,956	1.0% (0.2, 1.8)	0.9% (0.2, 1.8)	0.6% (0.2, 1.2)	59.5% (<0.01)
**South Africa**	10	22,716	1.3% (0.1, 2.3)	1.5% (0.8, 1.7)	1.2% (0.8, 1.7)	83.7% (<0.01)
**Uganda**	8	7,382	1.4% (0.0, 2.5)	1.4% (0.6, 2.2)	1.4% (0.7, 2.1)	75.3% (<0.01)
**Zimbabwe**	3	7,520	1.9% (0.3, 3.1)	2.2% (0.3, 3.1)	1.0% (0.7, 1.4)	-
**Living with HIV**	10	5,233	1.4% (0.0, 3.1)	1.6% (0.7, 1.9)	0.9% (0.4, 1.5)	61.0% (<0.01)
**HIV negative**	3	2,113	2.1% (0.3, 4.4)	1.4% (0.3, 4.4)	1.6% (0.1, 4.7)	92.6% (<0.01)

* N=5 studies had a prevalence estimate that was greater than 1.5 times the interquartile range (IQR) of all included studies. Excluded studies and details are listed in Supplementary Table B.15.

Table B.7. Pooled Prevalence of Stillbirth– Overall, by MTN-042 Country, and by HIV status

	# of estimates	# of pregnancies/ infants	Mean (Min, Max)	Median (IQR)	Pooled Prevalence (95% CI)	I^2 (p-value)
**Study Countries**	75	1,498,361	2.8% (0, 11.0)	2.3% (1.6, 3.8)	2.5% (2.2, 2.7)	98.0% (<0.01)
**Malawi**	18	536,079	2.6% (0.1, 7.3)	1.9% (1.7, 3.4)	2.3% (1.8, 2.9)	96.9% (<0.01)
**South Africa**	26	871,383	2.4% (0.0, 5.0)	2.3% (1.5, 3.7)	2.0% (1.7, 2.4)	98.0% (<0.01)
**Uganda**	23	66,533	3.3% (0.0, 8.8)	2.7% (1.8, 4.5)	3.0% (2.1, 4.1)	97.6% (<0.01)
**Zimbabwe**	6	24,003	2.4% (1.2, 5.6)	1.7% (1.3, 3.2)	2.0% (0.4, 4.7)	98.4% (<0.01)
**Multiple countries**	2	363	7.2% (3.4, 11.0)	7.2% (3.4, 11.0)	8.1% (5.5, 11.2)	-
**Living with HIV**	17	13,377	3.4% (0.0, 11.0)	2.6% (1.9, 4.4)	2.9% (2.0, 3.8)	81.5% (<0.01)
**HIV negative**	9	9,510	2.2% (0.0, 5.4)	2.2% (1.4, 2.9)	1.9% (1.3, 2.5)	54.0% (<0.01)

Figure B.7. Forest Plot Summarizing Pooled Prevalence of Stillbirth - Malawi

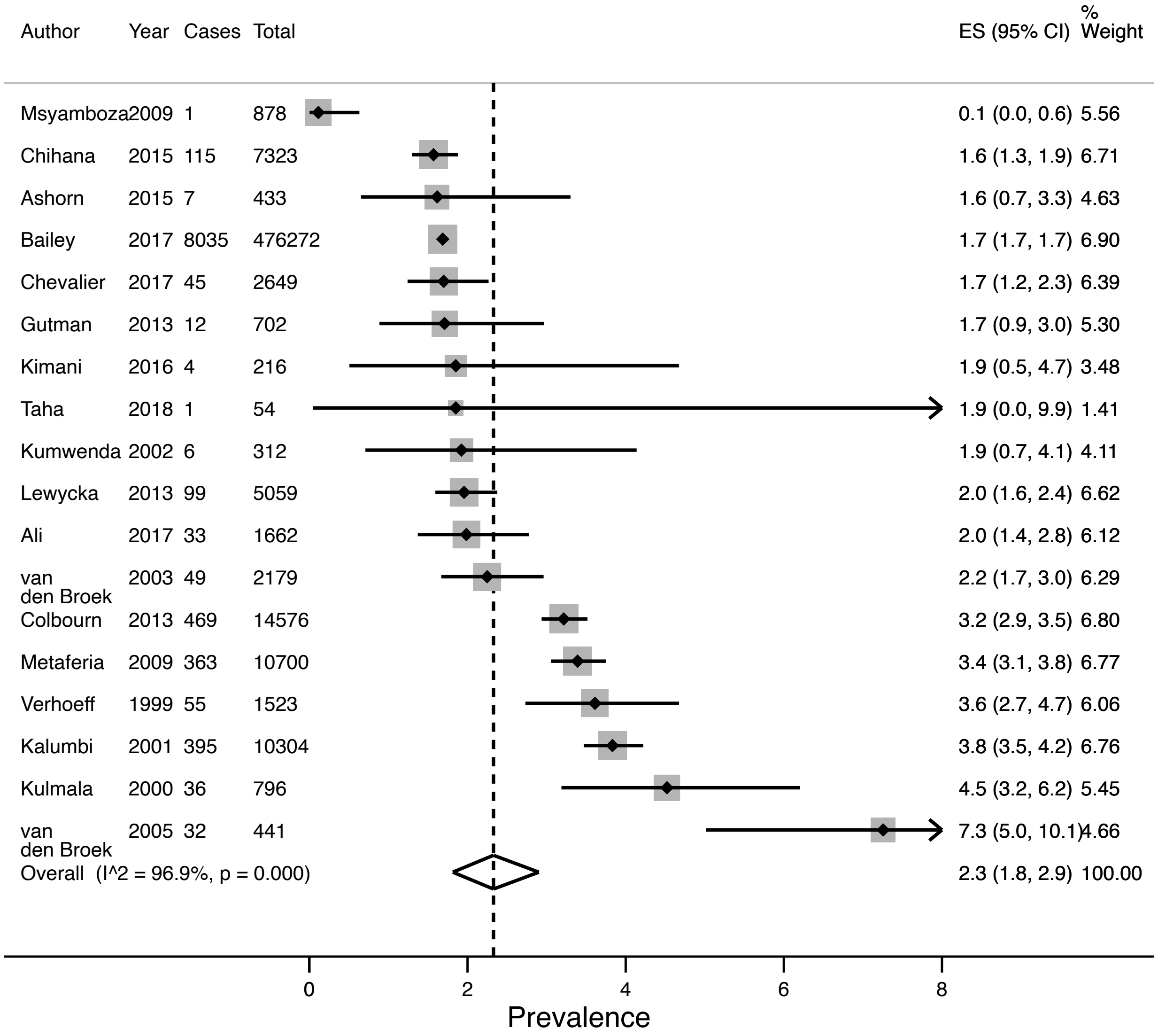

Figure B.8. Forest Plot Summarizing Pooled Prevalence of Stillbirth - South Africa

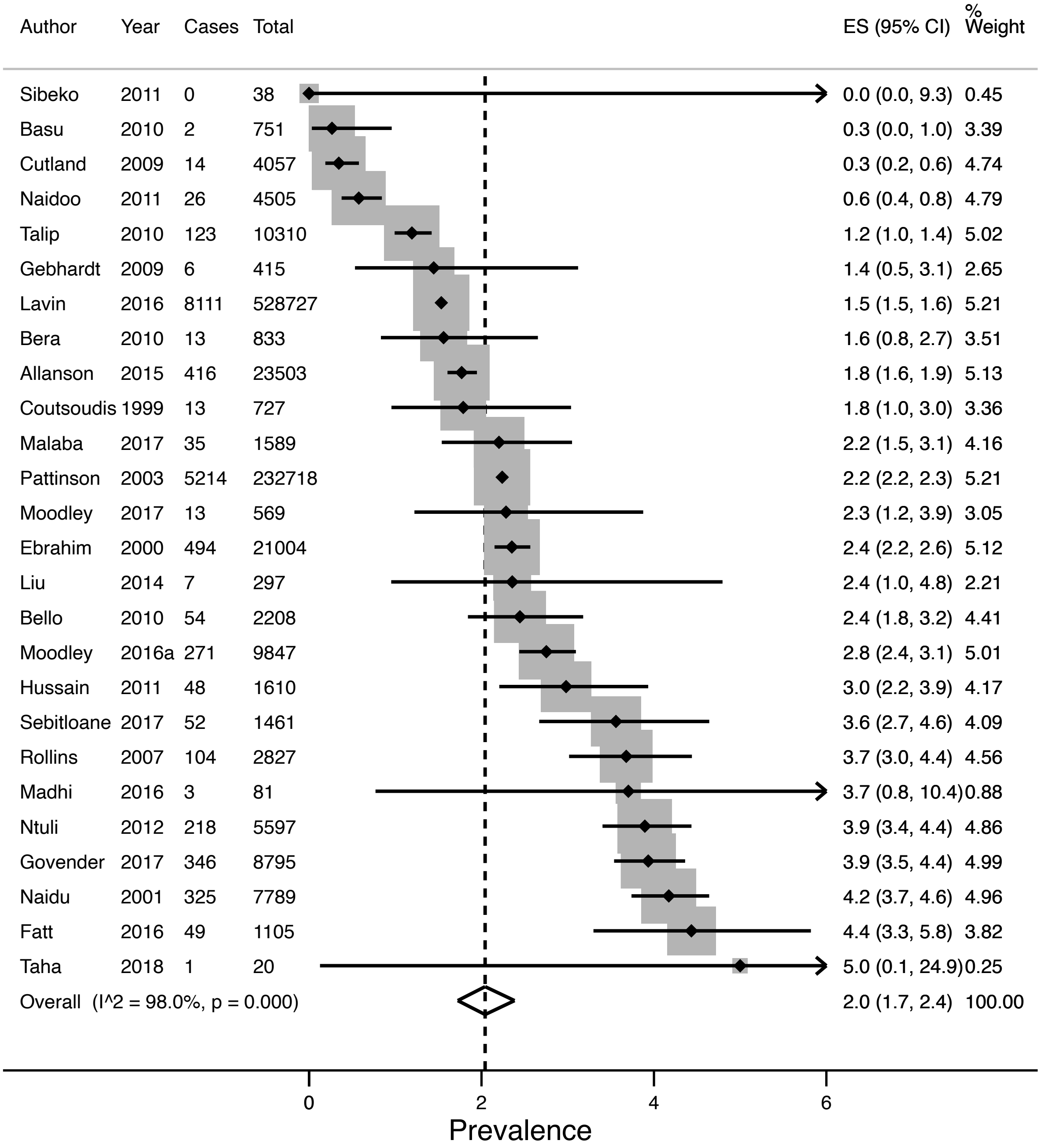

Figure B.9. Forest Plot Summarizing the Pooled Prevalence of Stillbirth - Uganda

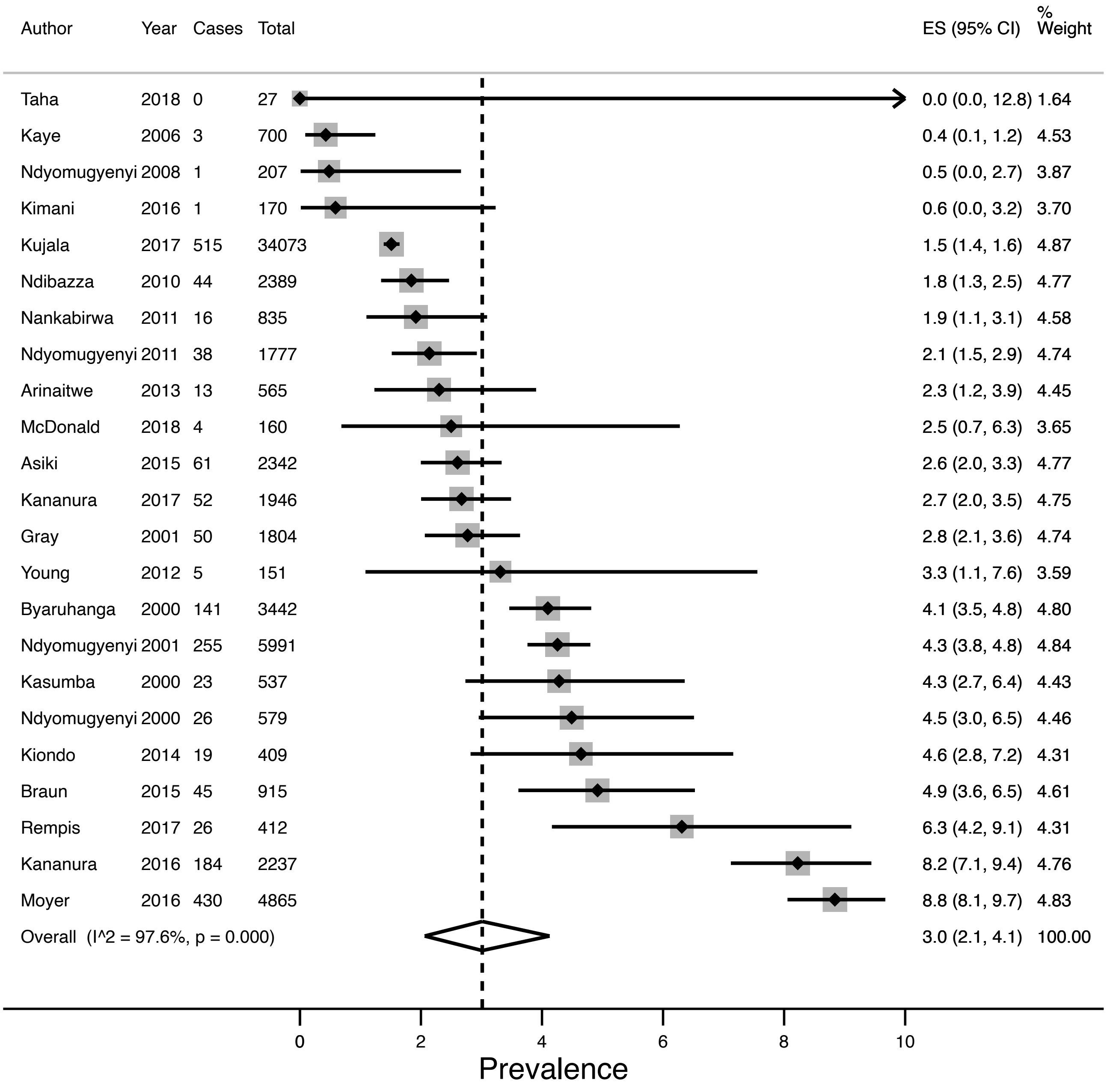

Figure B.10. Forest Plot Summarizing the Pooled Prevalence of Stillbirth - Zimbabwe

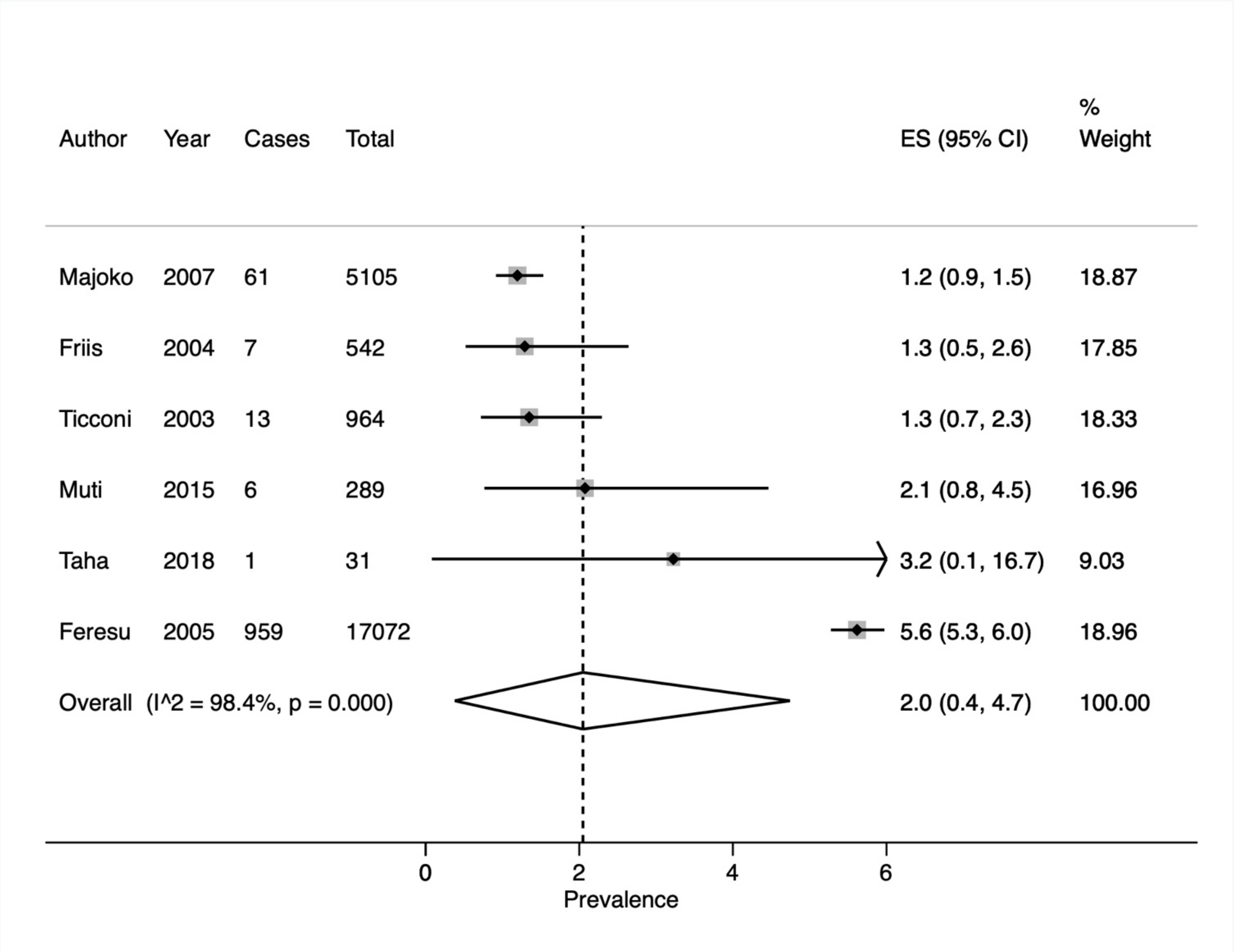

Figure B.11. Forest Plot Summarizing the Pooled Prevalence of Stillbirth among Women Living with HIV

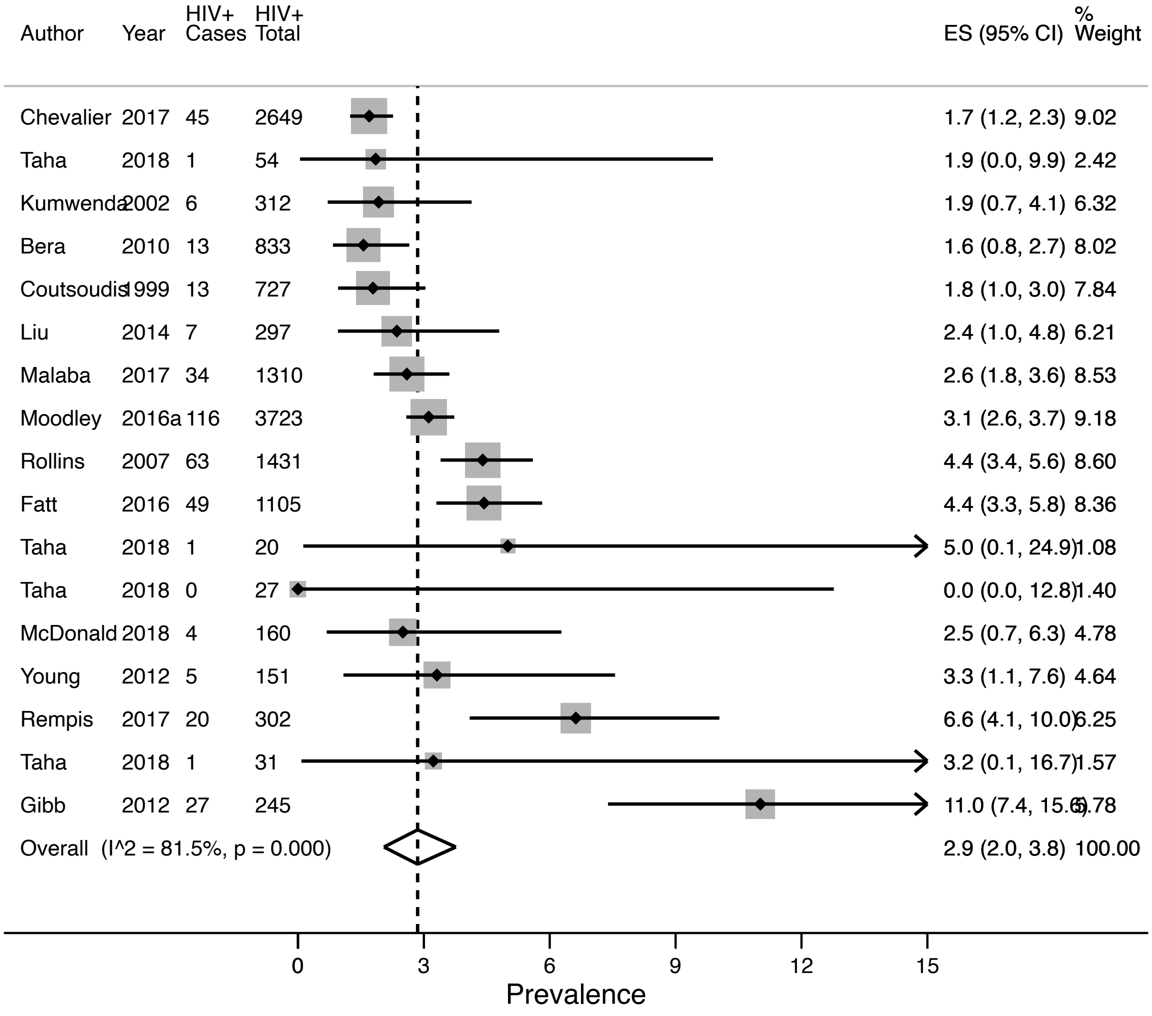

Figure B.12. Forest Plot Summarizing the Pooled Prevalence of Stillbirth among HIV Negative Women

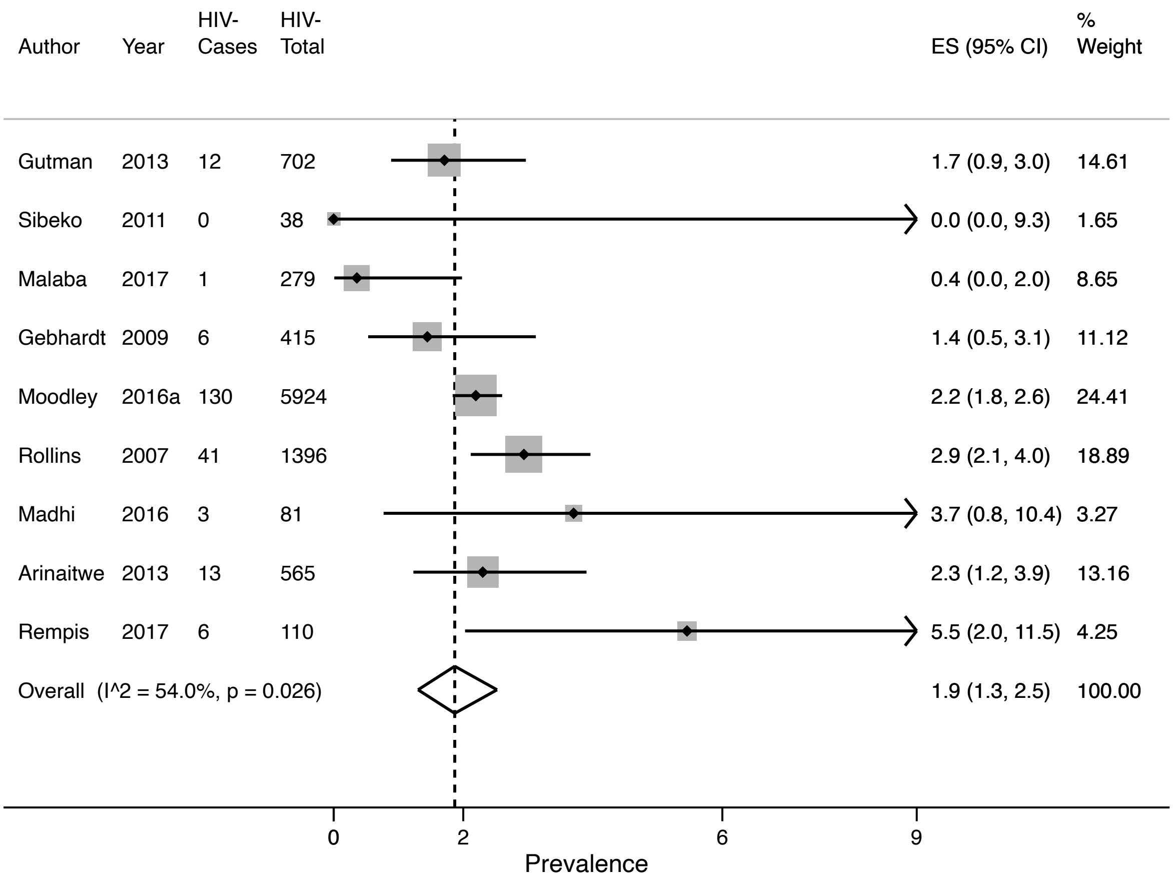

Table B.8. Sensitivity Analysis Excluding Studies with Stillbirth Definition Undefined: Pooled Prevalence of Stillbirth – Overall, by MTN-042 Country, and by HIV status

	# of estimates*	# of pregnancies/ infants	Mean (Min, Max)	Median (IQR)	Pooled Prevalence (95% CI)	I^2 (p-value)
**Study Countries**	37	1,125,008	3.2% (0.0, 11.0)	2.4% (1.8, 3.9)	2.7% (2.3, 3.1)	98.3% (<0.01)
**Malawi**	9	487,582	2.7% (1.6, 7.3)	1.9% (1.7, 2.0)	2.3% (1.7, 3,0)	87.4% (0.01)
**South Africa**	10	568,980	2.5% (0.6, 5.0)	2.3% (1.8, 3.7)	1.9% (1.4, 2.5)	96.2% (<0.01)
**Uganda**	13	50,016	3.4% (0.0, 8.8)	2.6% (1.8, 4.6)	3.2% (1.6, 5.1)	98.4% (<0.01)
**Zimbabwe**	3	18,067	3.4% (1.3, 5.6)	3.2% (1.3, 5.6)	2.9% (0.2, 7.8)	-
**Multiple countries**	2	363	7.2% (3.4, 11.0)	7.2% (3.4, 11.0)	8.1% (5.5, 11.2)	-
**Living with HIV**	12	6,094	3.6% (0.0, 11.0)	2.9% (1.8, 4.7)	3.1% (1.8, 4.9)	84.4% (<0.01)
**HIV negative**	2	1,506	4.2% (2.9, 5.4)	4.2% (2.9, 5.4)	2.9% (2.1, 3.9)	-

* Included studies: Ali_2017(3), Allanson_2015 (4), Ashorn_2015 (7), Asiki_2015 (8), Bailey_2017 (10), Bello_2010 (14), Chevalier_2017 (25), Coutsoudis_1999 (28), Feresu_2005(39), Gibb_2012 (47), Kujala_2017 (71), Kananura_2016 (62), Kananura_2017(63), Kimani_2016 (69), Kiondo_2014 (70), Kulmala_2000 (72), Lavin_2016 (75), Lewycka_2013 (76), Liu_2014 (77), Makanani_2018 (82), McDonald_2018 (84),

Moodley_2017 (88), Moyer_2016 (89), Nankabirwa_2011 (100), Naidoo_2011(97), Ndibazza_2010 (102), Ntuli_2012(114
Rollins_2007 (122), Taha_2018 (134), Ticconi_2003 (138), Young_2012 (152), van den Broek_2005 (142)

Table B.9. Pooled Prevalence of Antepartum Stillbirth – Overall and by MTN-042 Country

	# of estimates*	# of pregnancies/ infants	Mean (Min, Max)	Median (IQR)	Pooled Prevalence (95% CI)	I^2 (p-value)
**Study Countries**	9	563,797	1.9% (0.4, 3.5)	1.6% (1.1, 2.7)	1.7% (1.1, 2.4)	99.2% (<0.01)
**Malawi**	3	501,539	0.9% (0.4, 1.6)	0.8% (0.4, 1.6)	0.8% (0.4, 1.4)	-
**South Africa**	3	36,889	2.0% (1.1, 2.8)	2.2% (1.1, 2.8)	2.0% (1.0, 3.2)	-
**Uganda**	2	8,297	2.5% (1.6, 3.5)	2.5% (1.6, 3.5)	2.6% (2.3, 3.0)	-
**Zimbabwe**	1	17,072	2.7% -	-	2.7% (2.4, 2.9)	-

* Included studies: Allanson_2015 (4), Bailey_2017 (10), Byaruhanga_2000 (22), Colbourn_2013 (27), Feresu_2005 (39), Metaferia_2009 (85), Moyer_2016 (89), Naidu_2001 (98), Ntuli_2012 (114)

Table B.10. Pooled Prevalence of Intrapartum Stillbirth – Overall and by MTN-042 Country

	# of estimates*	# of pregnancies/ infants	Mean (Min, Max)	Median (IQR)	Pooled Prevalence (95% CI)	I^2 (p-value)
**Study Countries**	10	564,042	2,2% (0.6, 5.1)	1.7% (1.1, 2.5)	1.8% (1.3, 2.4)	98.5% (<0.01)
**Malawi**	3	501,539	1.4% (0.9, 1.8)	1.6% (0.9, 1.8)	1.4% (0.8, 2.1)	-
**South Africa**	3	36,889	1.2% (0.6, 1.9)	1.1% (0.6, 1.9)	1.2% (0.5, 2.1)	-
**Uganda**	2	8,297	3.8% (2.5, 5.1)	3.8% (2.5, 5.1)	3.9% (3.5, 4.4)	-
**Zimbabwe**	1	17,072	1.2% -	-	1.2% (1.0, 1.4)	-
**Multiple Countries**	1	245	4.9% -	-	4.9% (2.6, 8.4)	-

* Included studies: Allanson_2015 (4), Bailey_2017 (10), Byaruhanga_2000 (22), Colbourn_2013 (27), Feresu_2005 (39), Gibb_2012 (47), Metaferia_2009 (85), Moyer_2016 (89), Naidu_2001 (98), Ntuli_2012 (114)

Table B.11. Sensitivity Analysis Including Studies Defining Stillbirth ≥20 Weeks Gestation: Pooled Prevalence of Stillbirth – Overall and by MTN-042 Country

	# of estimates*	# of pregnancies/ infants	Mean (Min, Max)	Median (IQR)	Pooled Prevalence (95% CI)	I^2 (p-value)
**Study Countries**	12	35,853	3.2% (0.0, 8.2)	2.9% (1.5, 4.4)	3.7% (1.4, 4.3)	97.5% (<0.01)
**Malawi**	2	2,703	1.8% (1.7, 1.9)	1.8% (1.7, 1.9)	1.3% (0.9, 1.8)	-
**South Africa**	2	5,617	4.4% (3.9, 5.0)	4.4% (3.9, 5.0)	2.9% (2.4, 3.4)	-
**Uganda**	4	4,361	3.6% (0.0, 8.2)	3.0% (1.3, 5.8)	3.5% (0.7, 7.9)	95.7% (<0.01)
**Zimbabwe**	4	23,172	2.8% (1.2, 5.6)	2.3% (1.3, 4.4)	2.3% (0.2, 6.1)	99.0% (<0.01)

* Included studies: Chevalier_2017 (25), Feresu_2005 (39), Kananura_2016 (62), Kananura_2017 (63), Majoko_2007 (81), Ntuli_2012 (114), Taha_2018 (134), Ticconi_2003 (138), Young_2012 (152)

Table B.12. Sensitivity Analysis Including Studies Defining Stillbirth ≥28 weeks Gestation: Pooled Prevalence of Stillbirth – Overall and by MTN-042 Country

	# of estimates*	# of pregnancies/ infants	Mean (Min, Max)	Median (IQR)	Pooled Prevalence (95% CI)	I^2 (p-value)
**Study Countries**	16	1,084,552	2.5% (0.6, 8.8)	1.8% (1.6, 2.4)	2.1% (1.8, 2.4)	97.9% (<0.01)
**Malawi**	3	481,547	1.8% (1.7, 2.0)	1.9% (1.7, 2.0)	1.6% (1.5, 1.8)	-
**South Africa**	5	557,759	1.6% (0.6, 2.4)	1.8% (1.5, 1.8)	1.4% (1.0, 1.8)	92.1% (<0.01)
**Uganda**	8	45,246	3.3% (0.6, 8.8)	2.2% (1.7, 4.4)	2.9% (1.2, 5.4)	98.8% (<0.01)
**Zimbabwe**	0	-	-	-	-	-
**Living with HIV**	4	1486	3.3% (1.8, 6.,6)	2.4% (2.1, 4.6)	3.1% (1.3, 5.5)	78.4% (<0.01)
**HIV negative**	1	110	5.5% -	-	5.5% (2.0, 11.5)	-

* Included studies: Allanson_2015 (4), Asiki_2015 (8), Bailey_2017 (10), Coutsoudis_1999 (28), Kujala_2017 (71), Kimani_2016 (69), Lavin_2016 (75), Lewycka_2013 (76), Liu_2014 (77), McDonald_2018 (84), Moyer_2016 (89), Nankabirwa_2011 (100), Naidoo_2011(97), Ndibazza_2010 (102), Rempis_2017 (119)

Table B.13. Sensitivity Analysis Excluding Outliers: Pooled Prevalence of Stillbirth – Overall, by MTN-042 Country, and by HIV status

	# of estimates*	# of pregnancies/ infants	Mean (Min, Max)	Median (IQR)	Pooled Prevalence (95% CI)	I^2 (p-value)
**Study Countries**	71	1,490,573	2.5% (0, 6.3)	2.2% (1.6, 3.6)	2.2% (2.0, 2.4)	97.5% (<0.01)
**Malawi**	17	535,638	2.3% (0.1, 4.5)	1.9% (1.7, 3.2)	2.2% (1.7, 2.7)	96.9% (<0.01)
**South Africa**	26	871,383	2.4% (0.0, 5.0)	2.3% (1.5, 3.7)	2.0% (1.7, 2.4)	98.0% (<0.01)
**Uganda**	21	59,431	2.7% (0.0, 6.3)	2.6% (1.8, 4.3)	2.6% (0.9, 1.5)	93.6% (<0.01)
**Zimbabwe**	6	24,003	2.4% (1.2, 5.6)	1.7% (1.3, 3.2)	2.0% (0.4, 4.7)	98.4% (<0.01)
**Multiple countries**	1	118	3.4% -	-	3.4% (0.9, 8.5)	-
**Living with HIV**	16	13,132	2.9% (0.0, 6.6)	2.2% (0, 5.4)	2.5% (1.8, 3.2)	74.1% (<0.01)
**HIV negative**	9	9,510	2.2% (0.0, 5.4)	2.2% (1.4, 2.9)	1.9% (1.3, 2.5)	54.0% (<0.01)

* N=4 studies had a prevalence estimate that was greater than 1.5 times the interquartile range (IQR) of all included studies. Excluded studies and details are listed in Supplementary Table B.14.

Notes B.1. Pregnancy Loss and Stillbirth Review and Meta-Analyses: Study Specific Notes

- Basu_2010 (12) & Ebrahim_2000(32) - Some overlap in study years at same study hospital
    - Potential duplicate population, but included both
- Lewycka_2013 (76)
    - Had a 6 month inception period, and an intervention period. Abstracted the intervention period because sample size was bigger
- Colbourn_2013 (27)
    - Presents data for a baseline period, then an intervention period that had a control and 3 intervention arms. Included the baseline period only because it contributed the largest number of infants
- Harrison_2007 (54)
    - Could not stratify pregnancy loss/stillbirth estimates by trial arm, included overall estimate

Table B.14. Pregnancy Loss and Stillbirth Meta-Analyses Outliers

Outcome	Manuscript	Country	Frequency	Definition	Outcome Ascertainment	Notes
**Pregnancy Loss**	Sibeko_2011(130)	South Africa	20.8%	Miscarriage – weeks not defined, does not include terminated pregnancies	Monthly pregnancy tests	CAPRISA 004 trial. Miscarriage rate includes early preclinical pregnancy losses.
	Naidoo_2011 (97)	South Africa	6.7%	Spontaneous miscarriage ≤28 weeks gestation	Interviews with the women	
	Bello_2010 (14)	South Africa	9.5%	Spontaneous abortion – weeks not defined	Retrospective self-report	
	Makanani_2018 (82)	Malawi, South Africa, Uganda, Zimbabwe	24.8%	Spontaneous abortion, weeks not defined; estimate does not include therapeutic/elective abortion or ectopic pregnancies	Prospective follow-up of women who became pregnant until pregnancy outcome occurred	ASPIRE trial
	Taha_2018 (134)	South Africa	9.1%	Abortion/miscarriage <20 weeks	Prospective follow-up of women who became pregnant for pregnancy outcome	PROMOTE study incident pregnancies. Small sample size (2/22 pregnancies)
**Stillbirth**	Moyer_2016 (89)	Uganda	8.8%	Stillbirth - Not defined but text is suggestive of ≥28 weeks	Records review	
	Van den Broek_2003 (142)	Malawi	7.3%	Not born alive – no weeks specified	Interviews with the women	

	Kananura_2017 (63)	Uganda	8.2%	≥20 weeks gestation	Face to face interview within 12 months of delivery	
	Gibb_2012 (47)	Uganda, Zimbabwe	11.0%	≥22 weeks gestation	Prospective follow-up	

Table B.15. Pregnancy Loss Study Definition – Included Studies

B.15A. Individual study characteristics

Author, Year	Study Design	Pregnancy Loss Definition										Gestational Age[*]	
		<20 weeks	≤20 weeks	<21 weeks	<22 weeks	<24 weeks	≤24 weeks	<28 weeks	≤28 weeks	2nd trimester	Weeks Not Reported	Inclusion/ Exclusion Criteria	Determination Method
Majoko, 2007 (81)	RCT	X										Enrolled at ANC booking	Fundal heights
Ticconi, 2003 (138)	Prospective Cohort	X										Admitted pregnant women. Median GA at enrollment was in 3rd trimester	Not reported
Taha, 2018 (134)	Prospective cohort	X										Incident pregnancies, age at confirmation not reported	Not reported
Ali, 2017(3)	Prospective cohort		X[†]									Mean GA at enrollment=34.7 and 33.9 weeks	Not reported
Young, 2012 (152)	Prospective cohort		X									12-28 weeks	LMP and ultrasounds
Moodley, 2017 (88)	Retrospective Cohort			X[†]								Enrolled at ANC registration; median GA at enrollment=24 weeks	<24 weeks=obstetric ultrasounds; ≥24 weeks=fundal heights, LMP, and palpation
Kulmala, 2000 (72)	Prospective cohort				X							Enrollment at ANC registration	Fundal height
Ashorn, 2015 (7)	RCT				X							<20 weeks	Ultrasound

Rollins, 2007 (122)	Prospective cohort						X					Enrolled at ANC	LMP
Kiondo, 2014 (70)	RCT						X					12-22 weeks	Not reported
Van den Broek, 2005 (142)	Prospective cohort					X						<24 weeks	Ultrasound
Bera, 2010 (16)	Prospective cohort					X						≥14 weeks	Ultrasound
Liu, 2014 (77)	Prospective cohort							X				Enrolled at ANC or HIV care clinic	LMP and ultrasound
Kimani, 2016 (69)	RCT							X†				14-26 weeks	Ultrasound
Ndibazza, 2010 (102)	RCT							X				In 2nd or 3rd trimester per midwife assessment	Not reported
Harrison, 2007 (54)	RCT								X			Enrolled at first ANC visit	LMP and fundal height
Malaba, 2017 (83)	Prospective cohort								X			Women seeking ANC	LMP and fundal height
Lewycka, 2013 (76)	RCT								X			Incident pregnancies, participants visited monthly	Not reported
Naidoo, 2011(97)	Cross sectional								X†			Self-reported pregnancies	Self-report
Gebhardt, 2009 (45)	Prospective cohort									X		Enrollment at ANC booking	Ultrasounds
Sibeko, 2011 (130)	RCT										X	Incident pregnancies, monthly urine pregnancy testing	Not reported
Brittain, 2015 (21)	Prospective cohort										X	20-28 weeks	Fundal height
Talip, 2010 (135)	Cross sectional										X	n/a (delivery records review)	Ultrasound
Ndyomugyenyi, 2000 (105)	RCT										X	In 1st or 2nd trimester	Hackett's method
Kumwenda, 2002 (73)	RCT										X	18-28 weeks	LMP
Gray, 2001(49)	RCT										X†	Various. Approximately 1/3	Ballard score

												enrolled in each trimester.	
Bello, 2010(14)	Cross sectional										X[†]	Self-report on most recent pregnancy	Self-report
Ndyomugyenyi, 2011(108)	RCT										X[†]	≥16 weeks	Fundal height
Makanani, 2018 (82)	RCT										X[†]	Incident pregnancy, monthly urine pregnancy testing	LMP, ultrasound, and physical exam

B.15B. Summary of included studies by gestational age at enrollment details and likelihood of including first trimester pregnancy losses. Gray represents studies that did not include or included few first trimester pregnancies.

Gestational age at enrollment (or under assessment)	# of studies	Likelihood of including 1st trimester pregnancy losses
At the time of ANC booking or at ANC (no mean/median GA)	7	**Possible but few (**ANC typically initiated later in pregnancy)
Median or mean GA = 2nd or 3rd trimester	3	**Possible but few**
Incident pregnancy (with regular pregnancy testing)	4	**Likely**
≥12 weeks	2	**Possible but few**
<20 weeks, <24 weeks	2	**Not included**
≥14 weeks	6	**Not included**
Self-reported	2	**Possible**
Based on medical records after delivery	1	**Possible but few** since data collection from delivery records
1st or 2nd trimester	1	**Likely**
Any trimester	1	**Likely**

[*] ANC=antenatal care/clinic; GA=gestational age; LMP=last menstrual period
[†] Specifically defined as spontaneous abortions only.

Table B.16. Stillbirth Study Definition – Included Studies

Author, Year	Study Design	Stillbirth/IUFD Definition								Gestational Age*	
		>20 weeks	≤20 weeks	≥21 weeks	≥22 weeks	>24 weeks	>28 weeks	≥28 weeks	Weeks Not Reported	Inclusion/ Exclusion Criteria	Determination Method
Ntuli, 2012 (114)	Cross sectional	X								n/a	Not reported
Feresu, 2005 (39)	Cross sectional	X								>20 weeks	LMP
Young, 2012 (152)	Prospective Cohort	X								12-28 weeks	LMP and ultrasounds
Majoko, 2007 (81)	RCT	X								Enrolled at ANC booking	Fundal heights
Ticconi, 2003 (138)	Prospective Cohort		X							Admitted pregnant women. Median GA at enrollment was in 3rd trimester	Not reported
Kananura, 2017 (63)	Cross sectional		X							n/a	Self report
Kananura, 2016 (62)	Cross sectional		X							n/a	Self report
Chevalier, 2017 (25)	Prospective cohort		X							<30 weeks	Not reported
Taha, 2018 (134)	Prospective cohort		X							Incident pregnancies, age at confirmation not reported	Not reported
Moodley, 2017 (88)	Retrospective cohort			X						Enrolled at ANC registration; median GA at enrollment=24 weeks	<24 weeks=obstetric ultrasounds; ≥24 weeks=fundal heights, LMP, and palpation

Ali, 2017 (3)	Prospective cohort			X						Mean GA at enrollment=34.7 and 33.9 weeks	Not reported
Ashorn, 2015 (7)	RCT				X					<20 weeks	Ultrasound
Rollins, 2007 (122)	Prospective cohort					X				Enrolled at ANC	LMP
Kiondo, 2014 (70)	RCT					X				12-22 weeks	Not reported
Van den Broek, 2005 (142)	Prospective cohort					X				<24 weeks	Ultrasound
Bera, 2010 (16)	Prospective cohort					X				≥14 weeks	Ultrasound
Bailey, 2017 (10)	Cross sectional							X		n/a	Not reported
Allanson, 2015 (4)	Cross sectional							X		n/a	Fundal height
Rempis, 2017 (119)	Cross sectional							X		n/a	Finnstrom scoring
Moyer, 2016 (89)	Cross sectional							X		n/a	Not reported
Kimani, 2016 (69)	RCT							X		14-26 weeks	Ultrasound
Kujala, 2017 (71)	Cross sectional							X		n/a	Self-report
Liu, 2014 (77)	Prospective cohort							X		Enrolled at ANC or HIV care clinic	LMP and ultrasound
McDonald, 2018 (84)	Prospective cohort							X		12-28 weeks gestation	Not reported
Naidoo, 2011 (97)	Cross sectional							X		Self-reported pregnancies	Self-report
Ndibazza, 2010 (102)	RCT							X		In 2nd or 3rd trimester per midwife assessment	Not reported
Harrison, 2007 (54)	RCT						X			Enrolled at first ANC visit	LMP and fundal height
Malaba, 2017 (83)	Prospective cohort						X			Women seeking ANC	LMP and fundal height

Asiki, 2015 (8)	Cross sectional						X			n/a	Not reported
Lewycka, 2013 (76)	RCT						X			Incident pregnancies, participants visited monthly	Not reported
Lavin, 2016 (75)	Cross sectional						X			n/a	LMP, ultrasound, clinical examination
Nankabirwa, 2011 (100)	Prospective cohort						X			>7 months	Not reported
Coutsoudis, 1999 (28)	RCT						X			Recruited from ANC, mean age at enrollment 28 weeks	Not reported
Byaruhanga, 2000 (22)	Cross sectional								X	n/a	Not reported
Verhoeff, 1999 (146)	Prospective cohort								X	Enrolled at first ANC	Not reported
Makanani, 2018 (82)	RCT								X	Incident pregnancy, monthly urine pregnancy testing	LMP, ultrasound, and physical exam
Kaye_2006 (67)	Prospective cohort								X	>13 weeks	LMP, ultrasound, and clinical exam
Fatti_2016 (35)	Prospective cohort								X	Enrolled at maternal faciltiies	Not reported
Kumwenda_2002 (73)	RCT								X	18-28 weeks	LMP
Muti_2015 (93)	Cross sectional								X	n/a	Not reported
Ndyomugyenyi_2008 (107)	RCT								X	≥16 weeks	Fundal height and LMP
Kasumba_2000 (65)	Cross sectional								X	n/a	Physical examination
Ndyomugyenyi_2011 (108)	RCT								X	≥16 weeks	Fundal height
Ndyomugyenyi_2000 (105)	RCT								X	In 1st or 2nd trimester	Hackett's method
Ndyomugyenyi_2001 (106)	Cross sectional								X	n/a	Not reported
Pattinson_2003 (117)	Cross sectional								X	n/a	Not reported

Talip_2010 (135)	Cross sectional								X	n/a (delivery records review)	Ultrasound
Bello_2010 (14)	Cross sectional								X	Self-report on most recent pregnancy	Self-report
Govender_2017 (48)	Cross sectional								X	n/a	Not reported
Ebrahim_2000 (32)	Cross sectional								X	n/a	Not reported
Gebhardt, 2009 (45)	Prospective cohort								X	Enrollment at ANC booking	Ultrasounds
Basu_2010 (12)	Cross sectional								X	n/a	Ultrasound
Colbourn_2013 (27)	RCT								X	n/a	Not reported
van den Broek_2003 (144)	Cross sectional								X	n/a	Not reported
Msyamboza_2009 (95)	Prospective cohort								X	n/a	Not reported
Metaferia_2009 (85)	Cross sectional								X	n/a	LMP
Kalumbi_2001 (61)	Cross sectional								X	n/a	Not reported
Arinaitwe_2013 (6)	Cross sectional								X	n/a	Not reported
Madhi_2016 (79)	RCT								X	28-35 weeks	Ultrasound, LMP, and fundal height
Sibeko_2011 (130)	RCT								X	Incident pregnancies, monthly urine pregnancy testing	Not reported
Gutman_2013 (52)	Cross sectional								X	n/a	Ballard score
Friis_2004 (43)	RCT								X	22-36 weeks	LMP and fundal height
Gray_2001 (49)	RCT								X	Various. Approximately 1/3 enrolled in each trimester.	Ballard score
Braun_2015 (20)	Cross sectional								X	n/a	Not reported
Hussain_2011 (57)	Cross sectional								X	n/a	Ballard score
Moodley_2016 (86)	Cross sectional								X	n/a	Not reported

Naidu_2001 (98)	Cross sectional								X	n/a	Not reported
Sebitloane_2017 (127)	Cross sectional								X	n/a	Not reported
Kulmala_2000 (72)	Prospective cohort								X[†]	Enrollment at ANC registration	Fundal height
Chihana_2015 (26)	Prospective cohort								X	n/a	Not reported

[*] ANC=antenatal clinic/care; GA=gestational age; LMP=last menstrual period
[†] However, based on definition of abortion, it is likely the stillbirth definition are losses at ≥22 weeks.

SECTION C: PRETERM BIRTH

Table C.1. Search Result Flow

	N
**Title Review**	590
**Abstract Review**	265
**Manuscripts Reviewed***	189
**Manuscripts Included From Main Search†**	109
**Manuscripts Added From Other Searches‡**	48
**Total Manuscripts Abstracted**	157
**Total Manuscripts Included For Analysis – MTN 042 Countries Only§**	63

* Does not include number of references from systematic reviews reviewed.
† Includes systematic review reference reviews.
‡ Some of these include duplicate populations with the studies that were abstracted through the main search. These were removed at analysis.
§ Kimani_2016 (69) reported prevalence estimates for two study countries and Taha_2018 (134) reported estimates for four countries. These studies were counted as one manuscript each but provided multiple prevalence estimates in the analysis phase.

Table C.2. Pooled Prevalence of Preterm Birth – Overall, by MTN-042 Country, and by HIV status

	# of estimates *	# of pregnancies/ infants	Mean (Min, Max)	Median (IQR)	Pooled Prevalence (95%CI)	I^2 (p-value)
**Study Countries**	67	134,763	13.4% (0.0, 32.1)	14.4% (8.6, 18.4)	12.7% (11.2, 14.3)	98.4% (<0.01)
**Malawi**	13	9,850	14.7% (0.6, 23.8)	17.4% (11.3, 19.7)	13.5% (9.1, 18.5)	97.7% (<0.01)
**South Africa**	28	85,559	13.6% (0.0, 32.1)	13.7% (6.8, 19.5)	12.6% (10.0, 15.5)	99.2% (<0.01)
**Uganda**	15	11,066	12.0% (3.7, 23.5)	11.9% (4.8, 16.6)	11.4% (9.0, 14.1)	93.1% (<0.01)
**Zimbabwe**	10	28,063	13.9% (0.0, 23.9)	15.1% (10.7, 17.4)	14.6% (12.4, 16.9)	93.7% (<0.01)
**Multiple Countries†**	1	225	9.8% -	-	9.8% (6.2, 14.4)	-
**Living with HIV**	21	18,592	14.3% (0.0, 25.1)	16.1% (9.8, 21.8)	14.1% (11.0, 17.6)	97.2% (<0.01)
**HIV negative**	13	11,108	11.0% (0.6, 20.9)	9.7% (7.5, 16.4)	10.0% (5.7, 15.4)	98.1% (<0.01)

* No outliers were identified so no sensitivity analysis excluding outliers was conducted.
† Uganda+ Zimbabwe, Gibb_2012 (47)

Figure C.1. Forest Plot Summarizing the Pooled Prevalence of Preterm Birth – Malawi

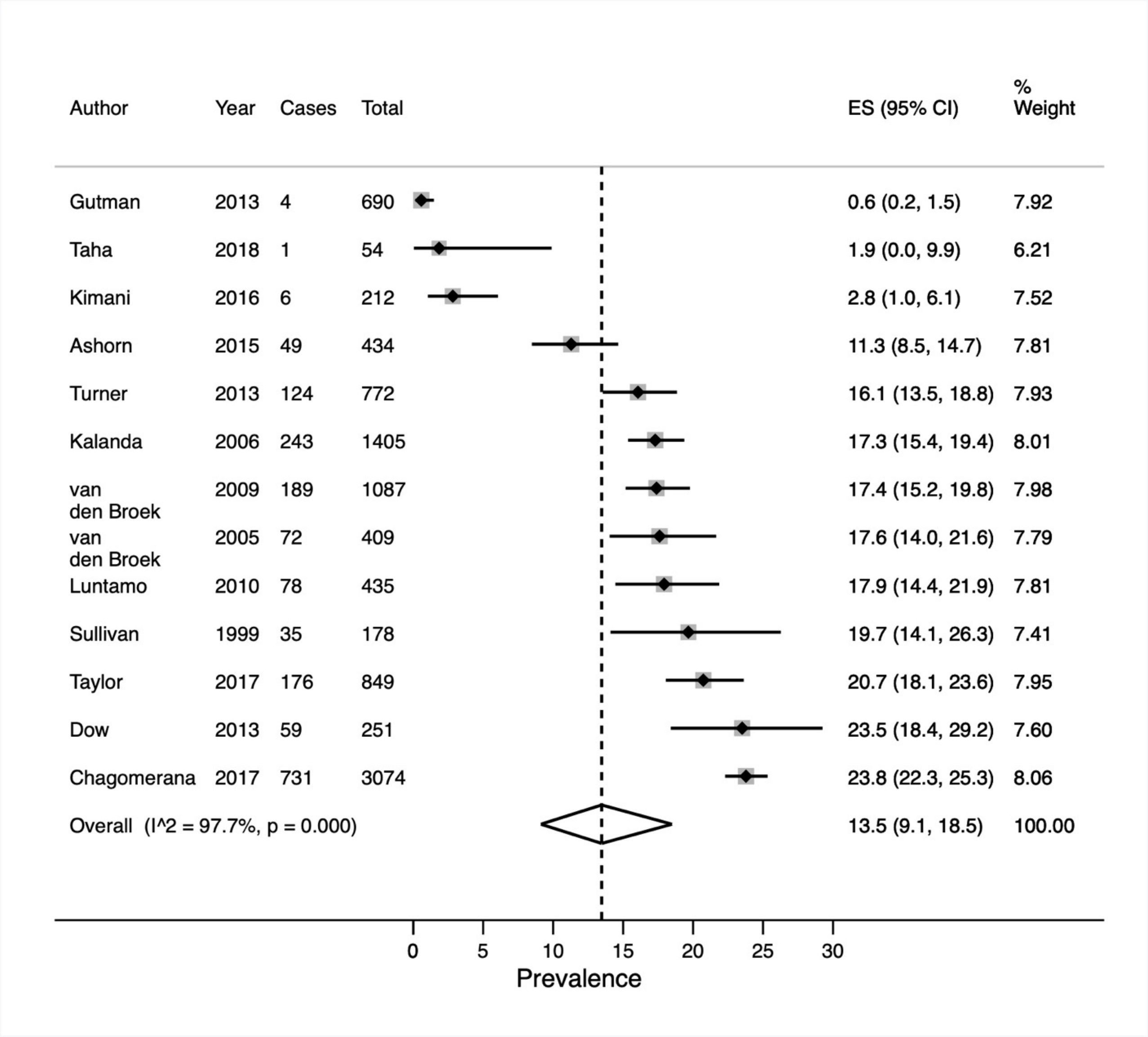

Figure C.2. Forest Plot Summarizing the Pooled Prevalence of Preterm Birth – South Africa

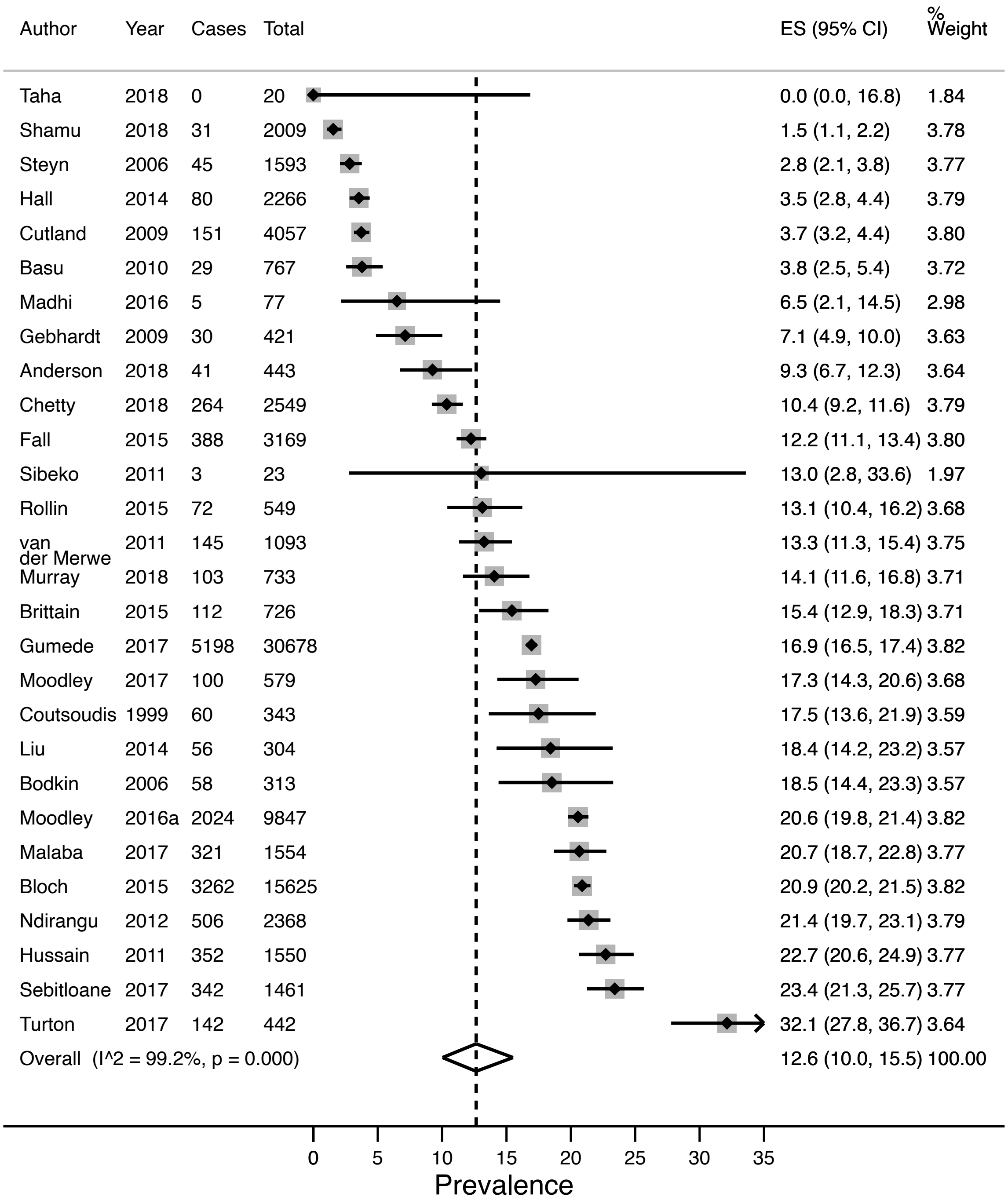

Figure C.3. Forest Plot Summarizing the Pooled Prevalence of Preterm Birth – Uganda

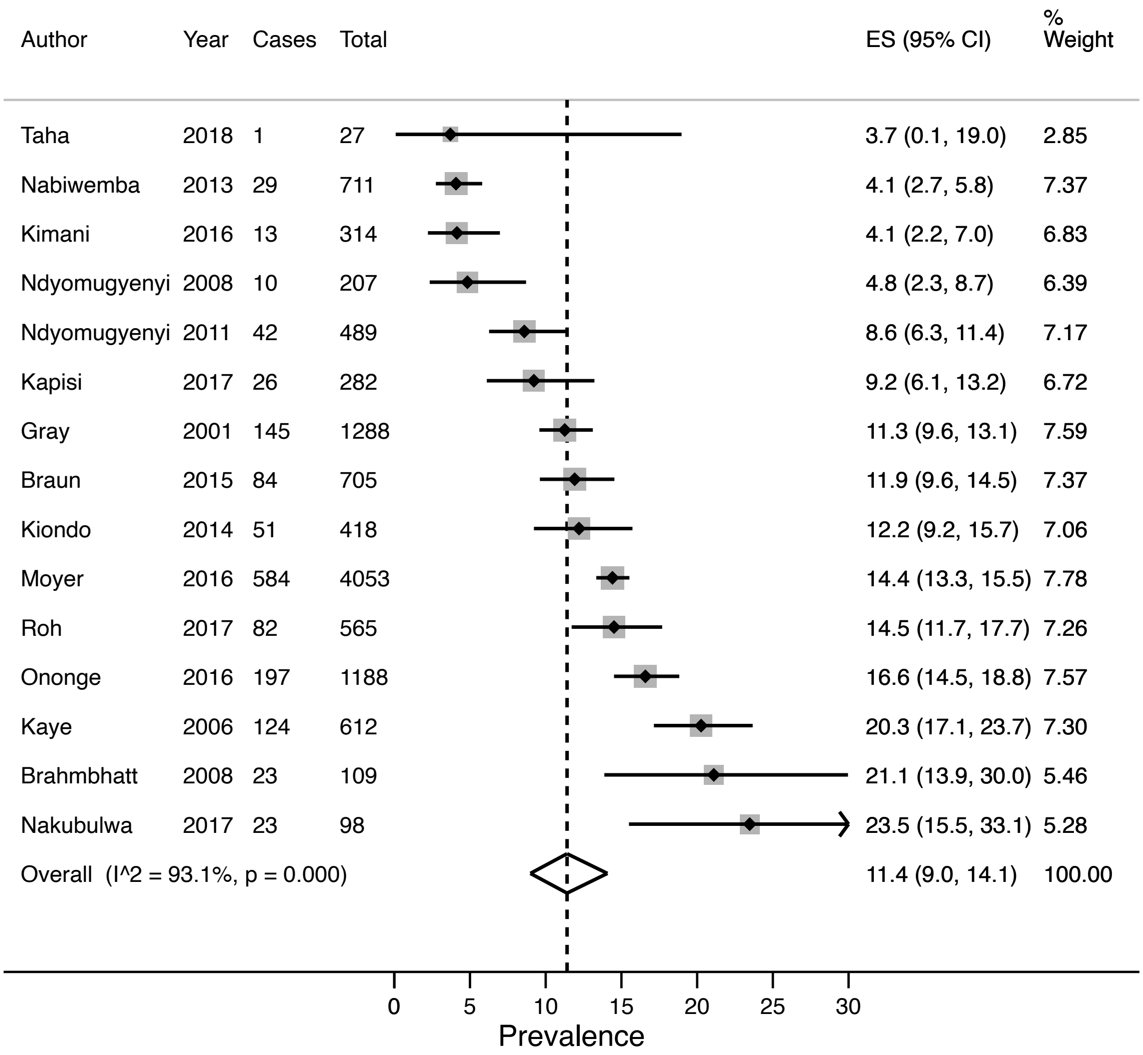

Figure C.4. Forest Plot Summarizing the Pooled Prevalence of Preterm Birth – Zimbabwe

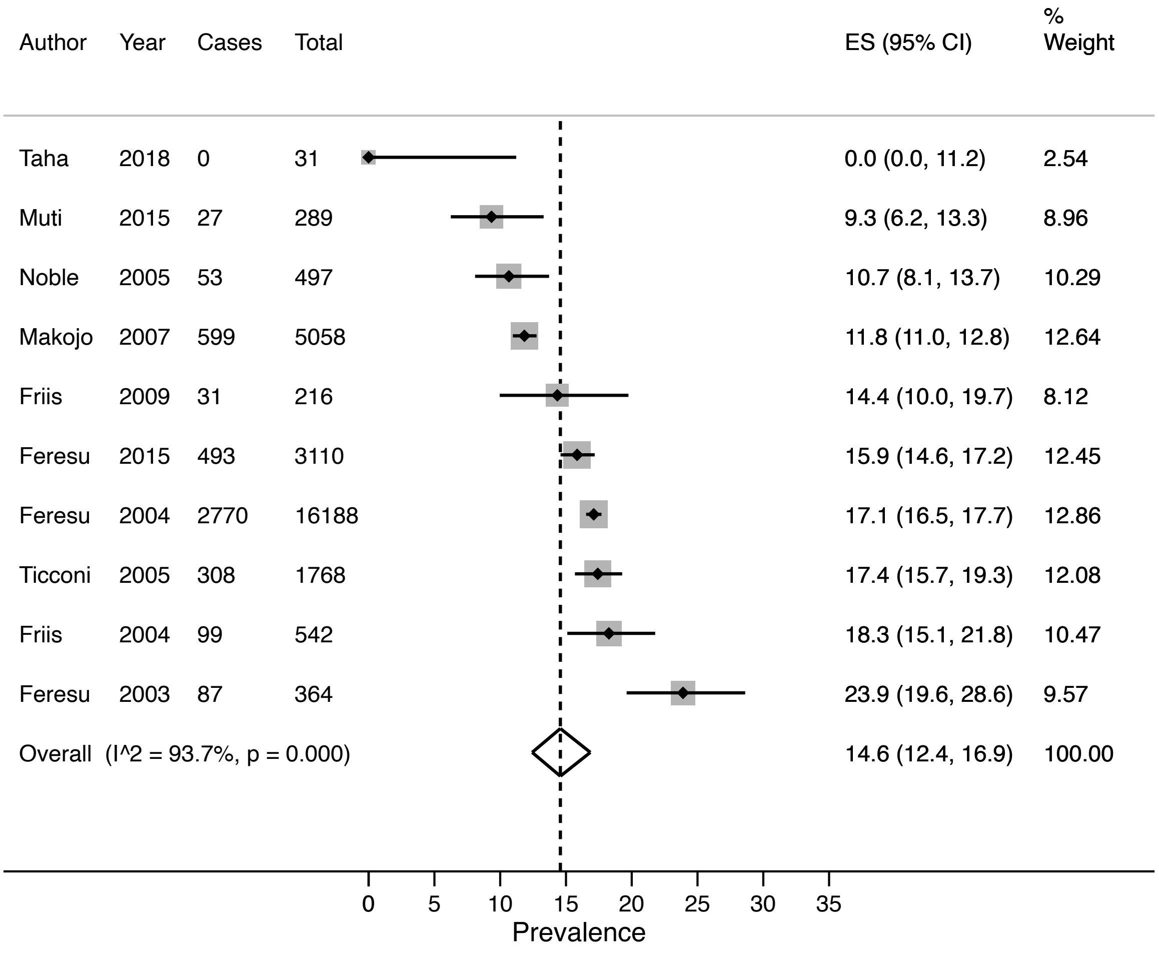

Figure C.5. Forest Plot Summarizing the Pooled Prevalence of Preterm Birth Among Women Living with HIV

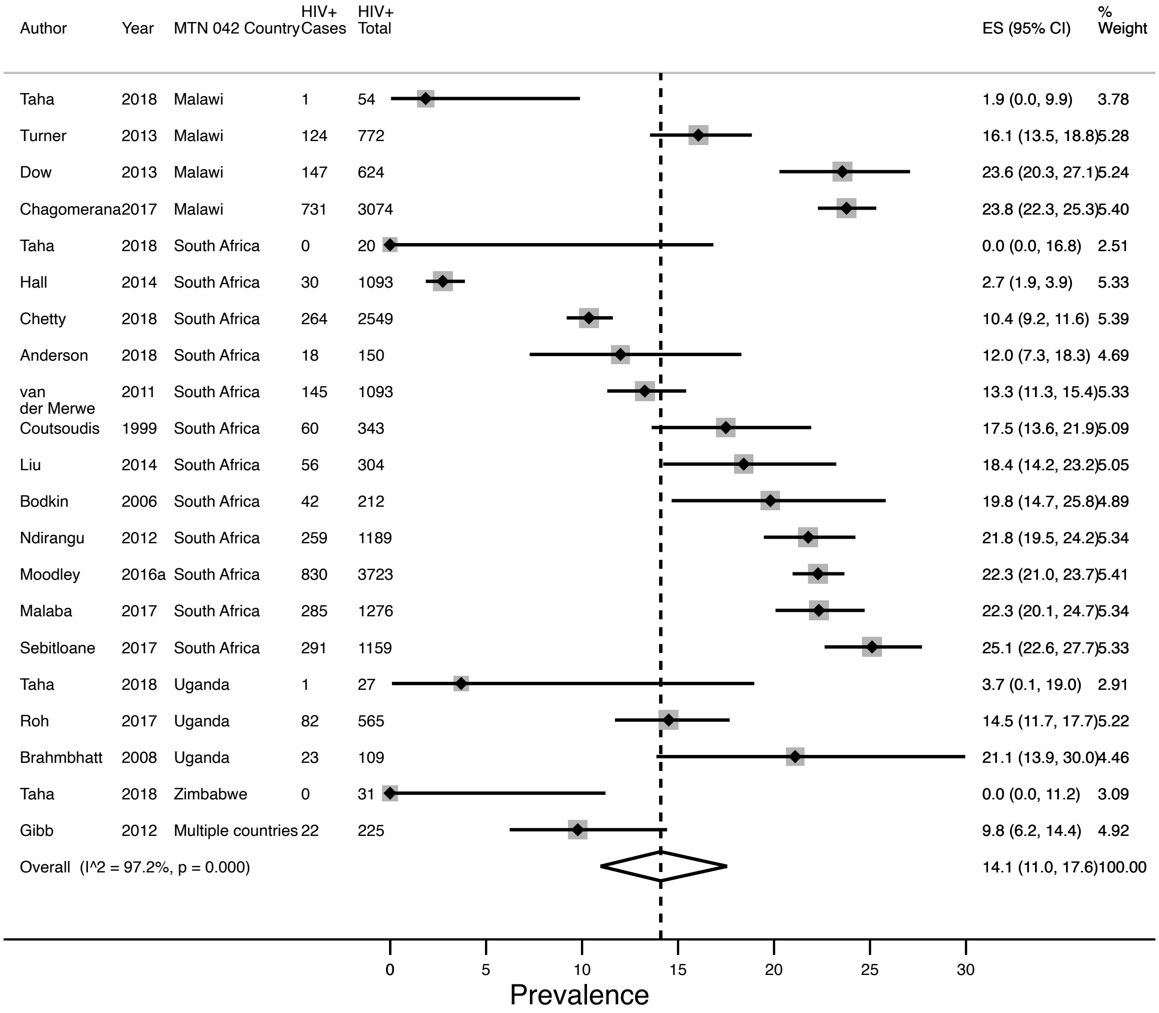

Figure C.6. Forest Plot Summarizing the Pooled Prevalence of Preterm Birth Among HIV Negative Women

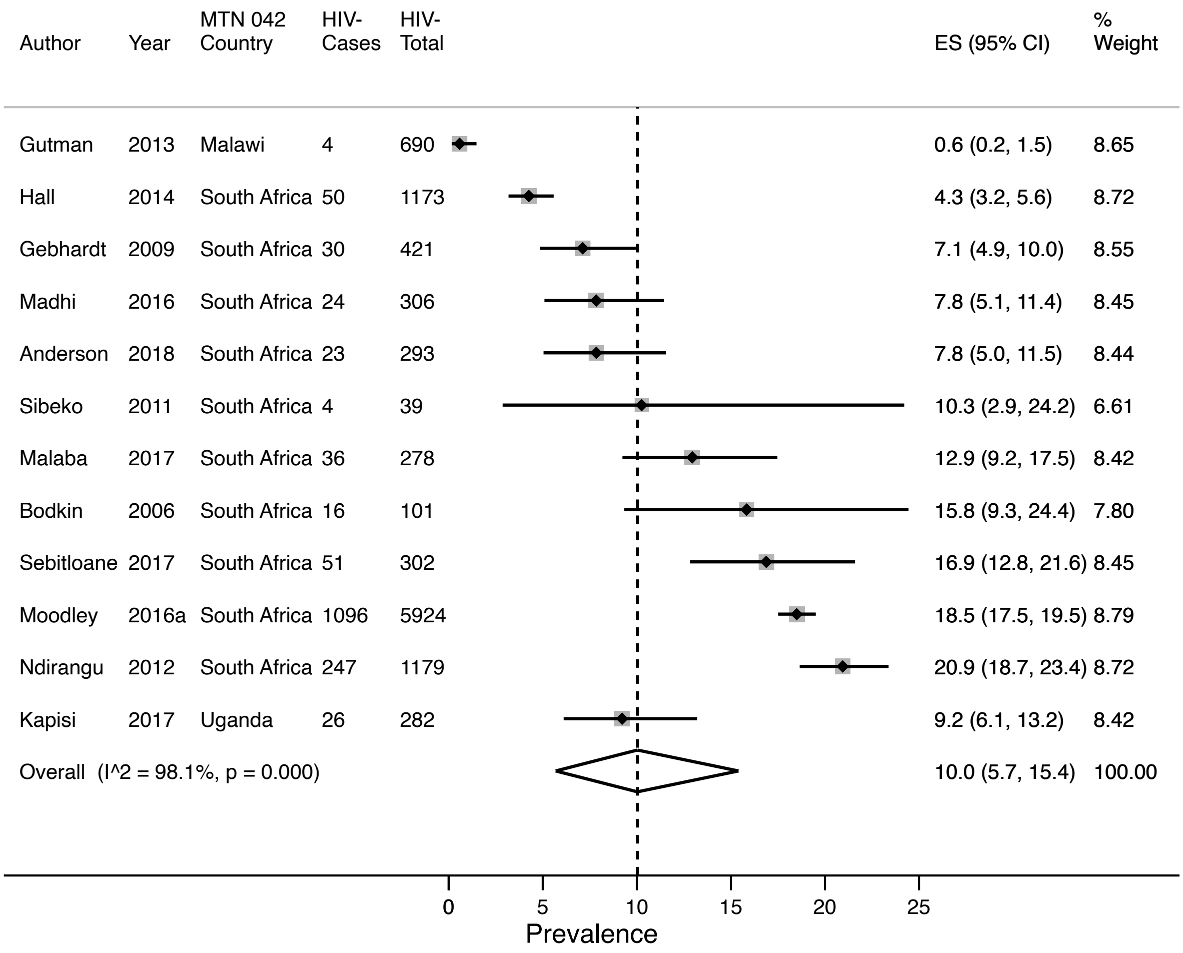

Table C.3. Sensitivity Analysis Live Birth Only: Pooled Prevalence of Preterm Birth – Overall, by MTN-042 Country, and by HIV Status

	# of estimates*	# of pregnancies/ infants	Mean (Min, Max)	Median (IQR)	Pooled Prevalence (95% CI)	I^2 (p-value)
**Study Countries**	30	47,187	14.0% (0.6, 32.1)	15.6% (8.6, 19.7)	12.7% (10.1, 15.6)	98.5% (<0.01)
**Malawi**	7	6,740	14.0% (0.6, 23.8)	17.3% (2.8, 19.7)	12.2% (5.7, 20.8)	98.7% (<0.01)
**South Africa**	13	16,707	15.0% (1.5, 32.1)	15.4% (10.4, 20.7)	13.8% (8.8, 19.6)	99.0% (<0.01)
**Uganda**	7	4,217	12.0% (4.1, 23.5)	11.3% (4.1, 20.3)	10.8% (6.7, 15.7)	95.2% (<0.01)
**Zimbabwe**	2	19,298	16.5% (15.9, 17.1)	16.5% (15.9, 17.1)	16.9% (16.4, 17.4)	-
**Multiple Countries†**	1	225	9.8% -	-	9.8% (10.6, 16.1)	-
**Living with HIV**	8	9,732	17.5% (9.8, 23.8)	18.0% (13.2, 22.1)	17.3% (13.0, 22.1)	96.9% (<0.01)
**HIV negative**	5	2,492	10.5% (0.6, 20.9)	10.3% (7.8, 12.9)	9.0% (1.4, 22.0)	98.8% (<0.01)

* Only studies where the preterm birth estimate was clearly defined as prevalence among live-births only were included. Included studies: Braun_2015 (20), Brittain_2015 (21), Chagomerana_2017 (23), Chetty_2018 (24), Coutsoudis_1999 (28), Fall_2015 (34), Feresu_2004 (38), Feresu_2015 (40), Gibb_2012 (47), Gray_2001 (49), Gutman_2013 (52), Hussain_2011 (57), Kalanda_2006 (59), Kaye_2006 (67), Kimani_2016 (69), Liu_2014 (77), Madhi_2016 (79), Malaba_2017 (83), Nabiwemba_2013 (96), Nakubulwa_2017 (99), Ndirangu_2012 (104), Ndyomugyenyi_2011 (108), Shamu_2018 (129), Sibeko_2011(130), Steyn_2006 (132), Sullivan_1999 (133), Turner_2013 (140), Turton_2013 (141), van den Broek_2005 (142)

† Uganda+ Zimbabwe, Gibb_2012 (47)

Table C.4. Sensitivity Analysis Excluding Studies with Preterm Birth Definition Undefined: Pooled Prevalence of Preterm Birth – Overall, by MTN-042 Country, and by HIV Status

	# of estimates*	# of pregnancies/ infants	Mean (Min, Max)	Median (IQR)	Pooled Prevalence (95%CI)	I^2 (p-value)
**Study Countries**	59	123,610	13.4% (0.0, 32.1)	14.1% (8.6, 18.3)	12.9% (11.4, 14.4)	98.1% (<0.01)
**Malawi**	13	9.850	14.7% (0.6, 23.8)	17.4% (11.3, 19.7)	13.5% (9.1, 18.5)	97.7% (<0.01)
**South Africa**	24	80,948	14.3% (0.0, 32.1)	14.7% (8,2, 20.6)	13.8% (11.3, 16.4)	98.9% (<0.01)
**Uganda**	12	6,292	10.4% (3.7, 23.5)	10.2% (4.4, 13.4)	9.9% (7.2, 12.9)	92.0% (<0.01)
**Zimbabwe**	9	26,295	13.5% (0.0, 23.9)	14.4% (10.7, 17.1)	14.1% (11.8, 16.7)	94.3% (<0.01)
**Multiple Countries†**	1	225	9.8% -	-	9.8% (6.2, 14.4)	-
**Living with HIV**	18	17,178	14.2% (0.0, 25.1)	15.3% (9.8, 22.3)	15.0% (12.3, 18.0)	95.5% (<0.01)
**HIV negative**	10	9,795	11.3% (0.6, 20.9)	9.2% (7.8, 16.9)	10.3% (5.5, 16.4)	99.3% (<0.01)

* Excluded studies: Bodkin_2006, Brahmbhatt_2008, Hall_2014, Kaye_2006, Moyer_2016, Shamu_2018, Sibeko_2011, Ticconi_2005
† Uganda+ Zimbabwe, Gibb_2012 (47)

SECTION D: CONGENITAL ANOMALIES

Table D.1. Search Result Flow

	N
**Title Review**	620
**Abstract Review**	75
**Manuscripts Reviewed**[*]	40
**Manuscripts Included From Main Search**[†]	15
**Manuscripts Added From Other Searches**	25
**Total Manuscripts Abstracted**	40
**Total Manuscripts Included For MTN-042 Analysis**[‡]	19

[*] Does not include # of references from systematic reviews reviewed
[†] Including systematic review reference reviews
[‡] Included all prevalence estimates regardless of whether it was reported for live births only or included stillbirths, and independent of HIV status. Some studies reported all congenital abnormalities, or all on a long list of possible abnormalities of interest. Other studies only reported no prevalence of specific anomalies (ex: NTDs, cleft lip) and do not report on other abnormalities that may have occurred in the same study population.

Table D.2. Pooled Prevalence of Congenital Anomalies – Overall, by MTN-042 Country, and by HIV status

	# of estimates*	# of pregnancies/ infants	Mean (Min, Max)	Median (IQR)	Pooled Prevalence (95%CI)	I^2 (p-value)
**Study Countries**	22	402,215	1.5% (0.0, 7.5)	0.3% (0.02, 2.7)	0.4% (0.2,0.7)	97.9% (<0.01)
**Malawi**	4	27,951	0.4% (0.0, 1.2)	0.2% (0.1,0.7)	0.2% (0.0, 0.6)	78.8% (<0.01)
**South Africa**	8	312,903	1.3% (0.0, 6.7)	0.1% (0.0, 1.6)	0.2% (0.0, 0.6)	98.5% (<0.01)
**Uganda**	7	60,997	1.6% (0.1, 6.0)	0.3% (0.1, 3.7)	0.6% (0.0, 1.5)	98.7% (<0.01)
**Zimbabwe**	1	31	0.0% -	-	0.0% (0.0, 11.2)	-
**Multiple Countries**	2	333	5.3% (3.1, 7.5)	5.3% (3.1, 7.5)	4.3% (2.3, 6.8)	-
**Living with HIV**	8	1,846	2.1% (0.0, 6.7)	1.8% (0.0, 3.4)	1.8% (0.5, 3.6)	67.8% (<0.01)
**HIV negative**	2	739	0.6% (0.0, 1.2)	0.6% (0.0, 1.2)	0.7% (0.1, 1.6)	-

* Naevus/birthmarks were excluded when possible. They were retained in the overall estimates when unable to confirm # of infants versus # anomalies.

Figure D.1. Forest Plot Summarizing the Pooled Prevalence of Congenital Anomalies

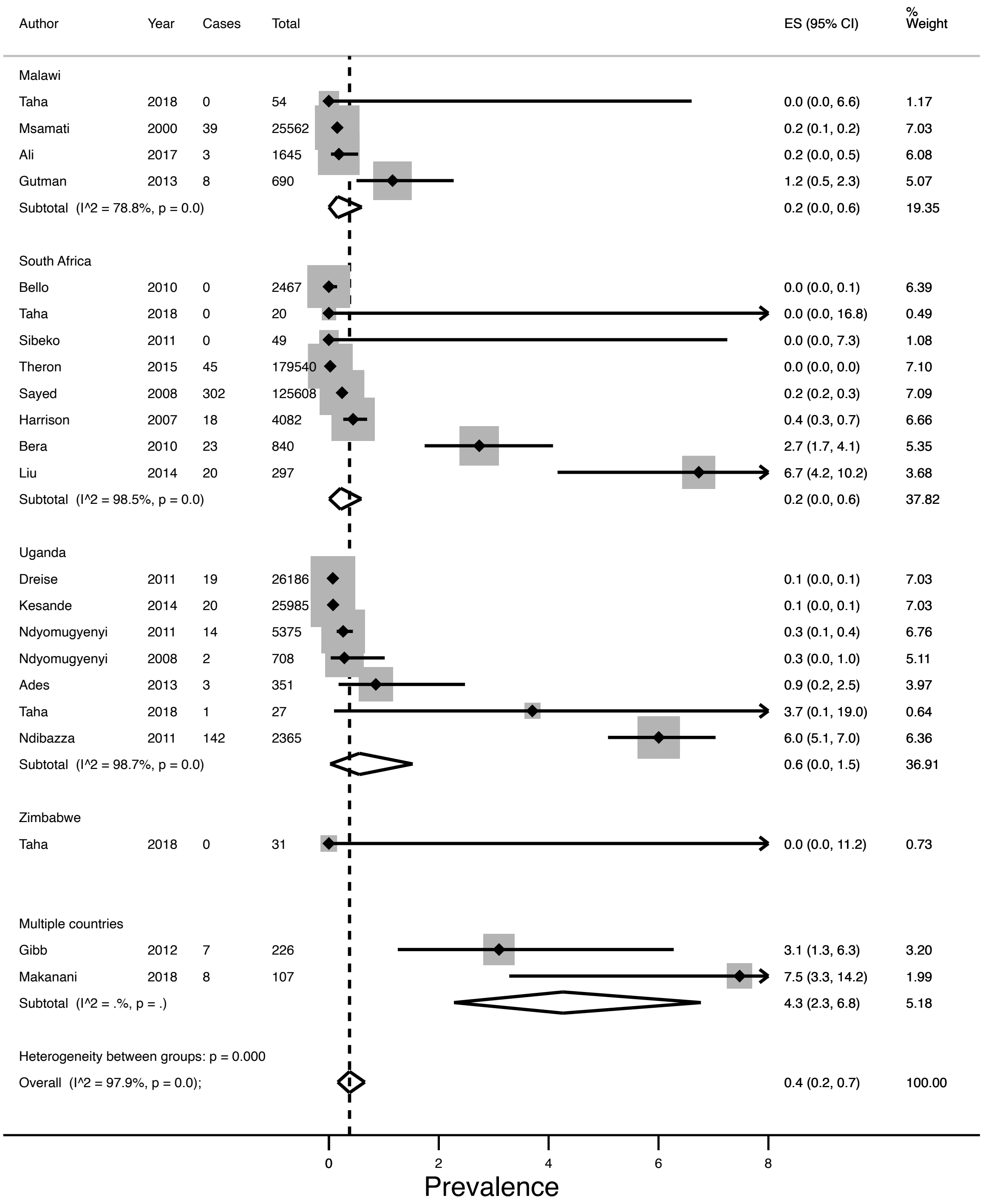

Figure D.2. Forest Plot Summarizing the Pooled Prevalence of Congenital Anomalies Among Women Living with HIV

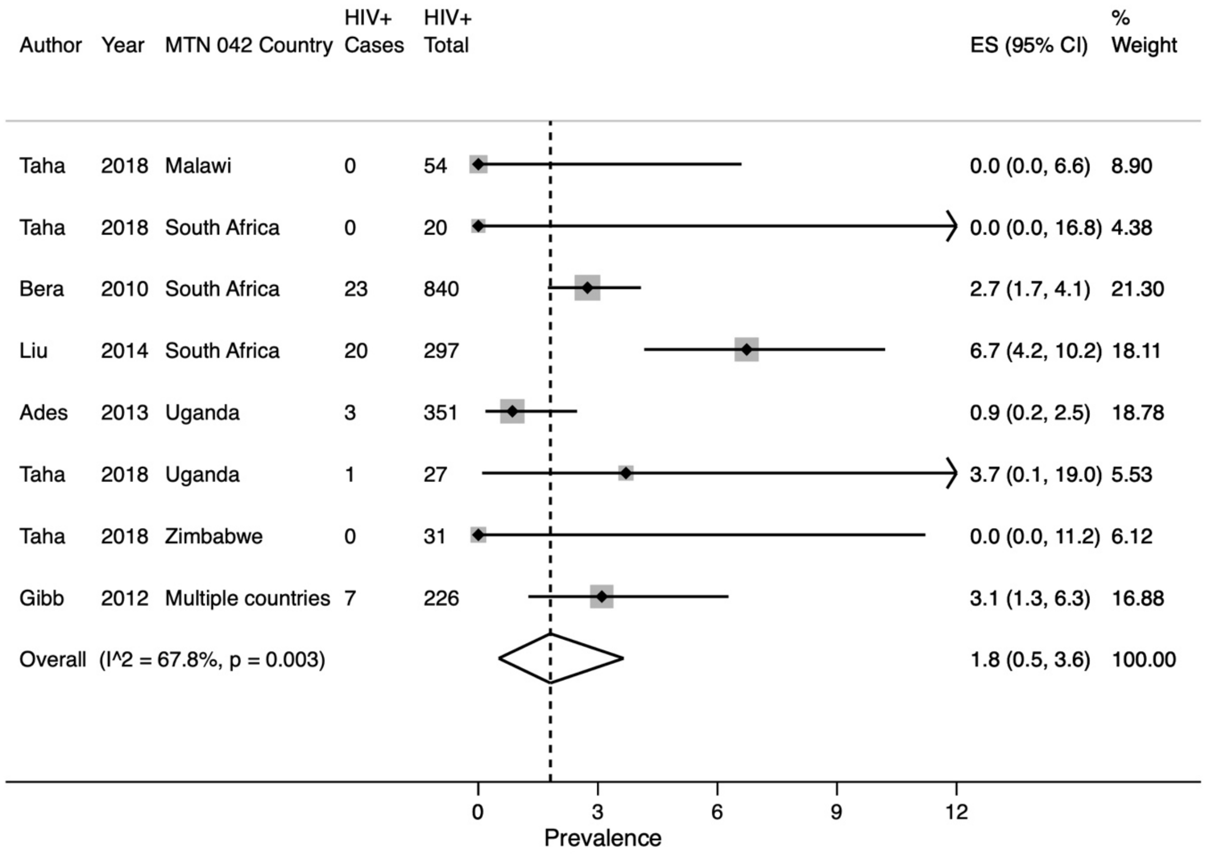

Note: Only two studies reported congenital anomaly prevalence among HIV negative women so no forest plot was generated.

Table D.3. Sensitivity Analysis Excluding Outliers: Pooled Prevalence of Congenital Anomalies – Overall, by MTN-042 Country, and by HIV status

	# of estimates*	# of pregnancies/ infants	Mean (Min, Max)	Median (IQR)	Pooled Prevalence (95%CI)	I^2 (p-value)
**MTN 042 Countries**	21	402,108	1.2% (0.0, 6.7)	0.2% (0.03, 1.2)	0.3% (0.1,0.6)	98.0% (<0.01)
**Malawi**	4	27,951	0.4% (0.0, 1.2)	0.2% (0.1,0.7)	0.2% (0.0, 0.6)	78.8% (<0.01)
**South Africa**	8	312,903	1.3% (0.0, 6.7)	0.1% (0.0, 1.6)	0.2% (0.0, 0.6)	98.5% (<0.01)
**Uganda**	7	60,997	1.6% (0.1, 6.0)	0.3% (0.1, 3.7)	0.6% (0.0, 1.5)	98.7% (<0.01)
**Zimbabwe**	1	31	0.0% -	-	0.0% (0.0, 11.2)	-
**Multiple Countries**	1	226	3.1% -	-	3.1% (1.3, 6.3)	-
**Living with HIV**	8	1,846	2.1% (0.0, 6.7)	1.8% (0.0, 3.4)	1.8% (0.5, 3.6)	67.8% (<0.01)
**HIV negative**	2	739	0.6% (0.0, 1.2)	0.6% (0.0, 1.2)	0.7% (0.1, 1.6)	-

[*] N=1 study had a prevalence estimate that was greater than 1.5 times the interquartile range (IQR) of all included studies. The excluded study was Makanani_2018 (82). Details in Supplemental Table D.7.

Table D.4. Sensitivity Analysis Excluding Non-Randomized Trial Data: Pooled Prevalence of Congenital Anomalies – Overall, by MTN-042 Country, and by HIV status

	# of estimates*	# of pregnancies/ infants	Mean (Min, Max)	Median (IQR)	Pooled Prevalence (95%CI)	I^2 (p-value)
**MTN 042 Countries**	8	13,263	2.3% (0.0, 7.5)	0.6% (0.3, 4.6)	1.5% (0.2, 3.6)	97.7%
**Malawi**	0	-	-	-	-	-
**South Africa**	2	4,131	0.2% (0, 0.4)	0.2% (0, 0.4)	0.1% (0.0, 0.3)	-
**Uganda**	4	8,799	1.9% (0.3, 6.0)	0.6% (0.3, 3.4)	1.3% (0.0, 5.1)	98.8%
**Zimbabwe**	0	-	-	-	-	-
**Multiple Countries**	2	333	5.3% (3.1, 7.5)	5.3% (3.1, 7.5)	4.3% (2.3, 6.8)	-
**Living with HIV**	2	577	2.0% (0.9, 3.1)	2.0% (0.9, 3.1)	1.6% (0.7, 2.8)	-
**HIV negative**	1	49	0%	0%	0.0% (0.0, 7.3)	-

* Includes studies using randomized trial data as a proxy for closer surveillance for anomalies. Included studies = Ades_2013 (2), Gibb_2012 (47), Harrison_2007 (54), Makanani_2018 (82), Ndibazza_2011 (103), Ndyomugyenyi_2008 (107), Ndyomugyenyi_2011 (108), Sibeko_2011 (130)

Figure D.3. Forest Plot Summarizing the Pooled Prevalence of Congenital Anomalies Among Women Living with HIV – Restricted to Studies Using Randomized Trial Data

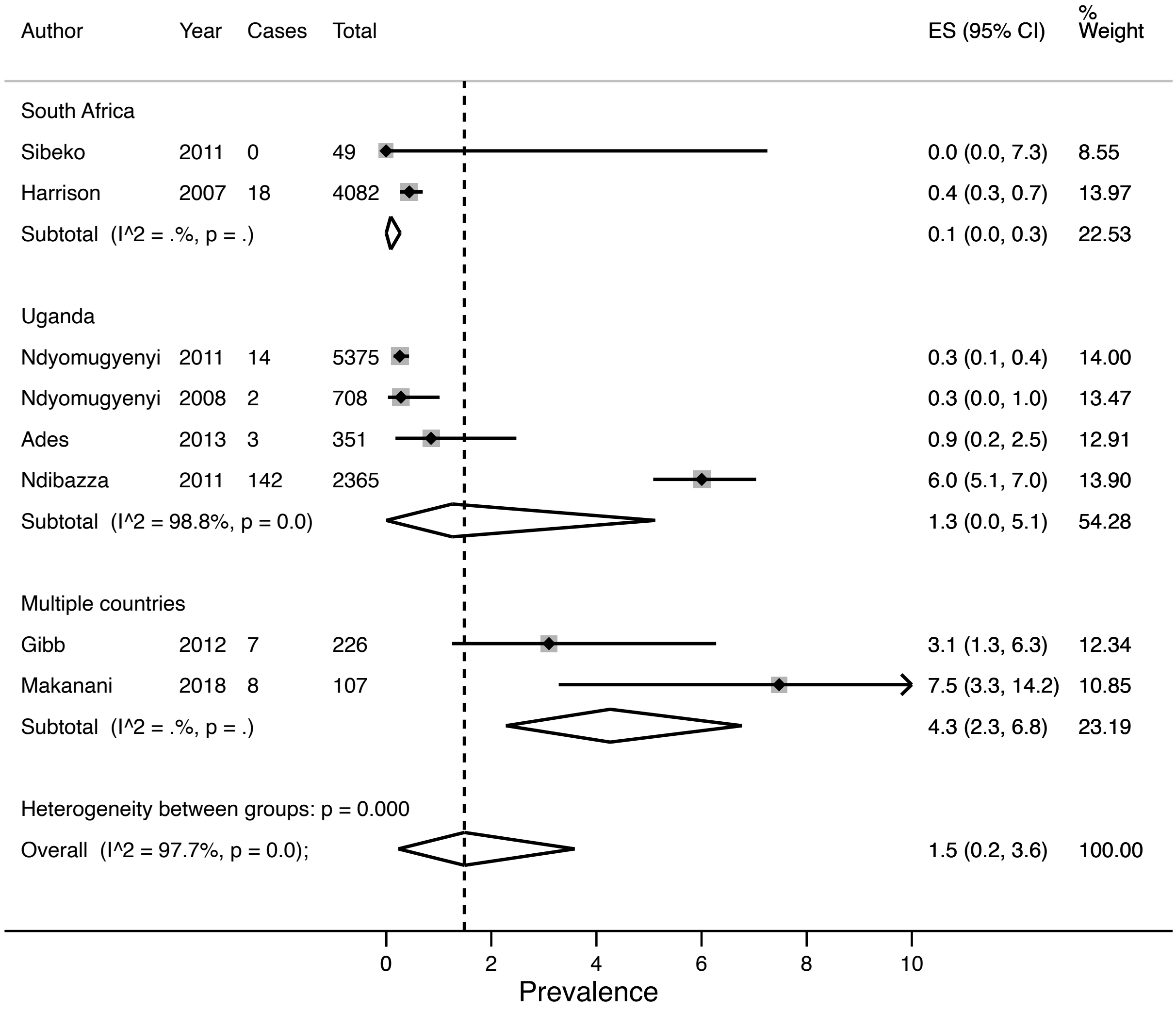

Table D.5. Overall pooled prevalence of specific and system-specific congenital anomalies

Anomaly Type*	# manuscripts	# cases	# of pregnancies/ infants at risk	Mean (Min, Max)	Median (IQR)	Pooled Prevalence (95%CI)	Per 1000	I^2 (p-value)
Not defined	8	49	13,372	0.75% (0.0, 3.10)	0.36% (0.13, 0.96)	0.34% (0.04, 0.82)	3.4	85.3% (<0.01)
Cleft Lip and/or Palate	8	111	205,537	0.14% (0.04, 0.34)	0.10% (0.07, 0.21)	0.03% (0.01, 0.05)	0.3	58.9% (0.02)
NTDs and/or Hydrocephalus	3	182	153,535	0.11% (0.09, 0.13)	0.12% (0.09, 0.13)	0.11% (0.08, 0.14)	1.1	32.2% (0.23)
Cardiovascular	3	8	3,502	0.43% (0.12, 1.01)	0.17% (0.12, 1.01)	0.23% (0.01, 0.65)	2.3	57.0% (0.10)
Polydactyly & Syndactyly	6	43	4,668	0.82% (0.14, 1.35)	1.00% (0.28, 1.14)	0.70% (0.30, 1.22)	7	56.4% (0.04)
Musculoskeletal including clubfoot	5	14	5,855	0.31% (0.06, 0.67)	0.28% (0.24, 0.30)	0.20% (0.07, 0.38)	2	25.7% (0.25)
Umbilical & Inguinal Hernia	4	79	3,609	2.25% (0.48, 4.67)	1.92% (0.74, 3.75)	1.73% (0.40, 3.81)	17.3	89.2% (<0.01)
Esophageal, gastrointestinal, or anorectal	3	47	182,745	0.06% (0.03, 0.12)	0.04% (0.03, 0.12)	0.02% (0.0, 0.07)	0.2	31.8% (0.23)
Genitourinary	2	8	2,662	0.46% (0.25, 0.67)	0.46% (0.25, 0.67)	0.23% (0.06, 0.48)	2.3	-
Trisomy	3	3	4,850	0.07% (0.04, 0.12)	0.07% (0.04, 0.12)	0.05% (0.05, 0.15)	0.5	0% (0.7)
Multiple Systems	1	10	2,365	0.42%	0.42%	0.42% (0.20, 0.78)	4.2	-
Other†	6	123	130,916	0.82% (0.06, 1.87)	0.58% (0.07, 1.71)	0.40% (0.06, 0.98)	4	91.5% (<0.01)

[*] Congenital anomalies were grouped into subtypes by common types (ex: NTDs) and then by system (ex: musculoskeletal). a subgroup was created when there was more than one reported case or study reporting the type/system of the anomaly. When multiple anomalies were listed per infant, the infant was included as one overall infant but was included as a case in each of the anomalies sub-types. If specific anomalies were not specified, such infants were included in the "multiple systems" sub-group. Naevus/birthmarks were excluded when possible for the overall and type specific analyses.

[†] Includes singular, or infrequent, reports of rare or non-specific anomalies that did not fit well into other defined sub-groups. Examples include natal tooth, anophthalmia, facial asymmetry, arachnoid cyst, hypopigmented skin, macrocephaly with brain defect, subtle dysmorphism, and plagiocephaly.

Table D.6. Congenital Anomaly: Study Definition

Author, Year	Study Design	Definition of Congenital Anomaly	Method for Ascertainment	Timing of Ascertainment	Anomalies Reported (diagnoses)	Notes
Msamati, 2000 (90)	Cross sectional	Cleft lip, cleft palate, spina bifida, hydrocephalus	Delivery and nursery records in obstetrics & gynecology and pediatrics departments	At (or near) time of delivery	Clefts (lip or palate), spina bifida, and hydrocephalus	Only assessed for specific set of anomalies
Gutman, 2013 (52)	Cross sectional	Physical abnormality	Not reported	At (or near) time of delivery	Details not reported.	
Ali, 2017 (3)	Cohort	Malformations = physical defect in a live infant that was identified by a clinician at designated health facilities thorough a clinical examination form that included a screening process to detect abnormalities	Physical examination (“Newborn babies presenting with health problems, per mothers’ reports, were referred to the designated health facility where a qualified medical professional did a medical examination to detect and manage any malformations or other health issues.”	Near time of delivery (exact timing not specified, visited at home by village health volunteer after delivery)	Congenital limb defect, Down's syndrome, and "not growing normally"	Children with abnormalities were referred to government health-care system for final diagnosis and care
Bera, 2010 (16)	Cohort	Birth defects	Physical examination by a pediatrician or senior obstetrician	At delivery (and potentially up to 6 weeks when mother/baby follow-up ended)	Arachnoid cyst, pulmonary stenosis, postaxial polydactyly, facial asymmetry and overlapping fingers, bilateral clubfeet, congenital naevus, and umbilical hernia, Trisomy 19, arthrogryposis multiplex congenita, oesophageal atresia with tracheo-oesophageal fistura, postaxpostaxial polydactyly of fingers and preaxial polydactyly with syndactyly of toes, and lower central incisor tooth	Pregnancy registry for EFV-based ART exposures
Sayed, 2008 (124)	Before/After	Neural Tube Defects (NTDs) and Non NTDs	Health care workers were trained on clinical recognition of the priority birth defects by	At or near time of delivery, or hospitalization	Anencephaly, spina bifida, encephalocoele, cleft lip, cleft	Assessment of NTDs before and

			a medical geneticist. Information on cases was extracted from hospital records.		palate, Down syndrome, and oculo-cutaneous albinism	after folic acid fortification in South Africa; primarily assessed for a specific set of anomalies
Theron, 2015 (137)	Cross sectional	Anorectal malformations	Reviewing the admission and theater registries and patient's medical records	Not clear; retrospective assessment but unclear ages of included patients	Anorectal malformation	Only included anorectal malformation s
Bello, 2010 (14)	Cross sectional	Malformation at birth	Retrospective, self report	At birth	No anomalies reported	
Liu, 2014 (77)	Cohort	A major congenital anomaly has an adverse outcome on either the function or the social acceptability of the individual, and a minor congenital anomaly has no medical or cosmetic importance	South Africa: examination by specialists and infants with suspected congenital anomalies by the clinical geneticist.<br>Zambia: study- midwives trained to assess for congenital anomalies by physical exam.	Birth, 6 weeks, 3 months, 6 months, 1 year	Major: Ambiguous genitalia, bilateal club foot, BPES, cleft lip, genu recurvartum, hypospadias, optic nerve atrophy, persistent foramen ovale, plagiocephaly, secundum atrial septal defect, syndactyly, ventricular septal defect, macrocephaly with brain atrophy<br><br>Minor: hypopigmented skin, polydactyly, umbilical hernia, preauricular sinus and cyst, preauricular tag, strabismus, subtle dysmorphism	ART registry
Sibeko, 2011 (130)	RCT	Not defined	On pregnancy cessation, the clinical staff collected data on pregnancy complications and documented pregnancy outcomes. Pregnant women presented their babies once in the early postpartum period to the study clinician for	Early postpartum	No anomalies reported for early neonatal period; live born only (term and preterm)	CAPRISA 004; zero anomalies reported so used the overall population total in the analysis

			assessment for abnormalities.			(versus placebo arm only)
Gibb, 2012 (47)	Cohort (secondary analysis of an RCT)	Not defined	Not specified	Near to delivery, length of potential follow-up unclear	Talipes, cardiac (atrial septal defect and patent ductus arteriousus), undescended testes, hydrochephalus, and skin tag	DART trial
Ndibazza, 2011(103)	Cohort (secondary analysis of an RCT)	Any structural defect present at birth	"Midwives carried out an external examination for overt anomalies, and no specific examination routine was followed. Anomalies detected by the physicians followed a thorough physical examination, supported when necessary by referral to specialists. Special investigations such as an echocardiogram were only done if there were clinical indication. Infants who had congenital anomalies involving more than one system were recorded once as having a multiple congenital anomaly. When no definite diagnosis was made, the infant was classified and coded as having an unspecified congenital anomaly." Anomalies were classified by organ system according to the ICD10.	At delivery and "accumulated to 1 year of age (per original trial methods, Ndibazza, 2010, CID)	GIT, Genitourinary, Cardiovascular, Dysmorphic, Head and neck, Multiple systems, Musculoskeletal, Central nervous system, Skin (birthmarks and poliosis), and unspecific cardiorespiratory	38 skin related abnormalities were reported. We excluded these cases from the prevalence analysis.
Ndyomugyenyi, 2011(108)	RCT	Not defined	Physical examination	At delivery, potentially through 28 days	Not reported	Used the overall population total in the analysis (versus

						placebo arm only)
Dreise, 2011 (31)	Cross sectional	All clefts	"The coordinator made the final diagnosis of the cleft, while a diagnosis of other defects was made by the plastic surgeons during the first visit of the baby to the cleft clinic."	At birth	Isolated cleft lip, cleft lip and palate, and isolated palate; three had other anomalies (constriction ring syndrome, trisomy 13 or 18, Pierre Robin syndrome)	Only assessed for specific set of anomalies; live birth only
Kesande, 2014 (68)	Cross sectional	Cleft lip and cleft palate	Medical records	At birth	Cleft lip and cleft palate	Only assessed for specific set of anomalies; live birth only
Ades, 2013 (2)	Cohort (secondary analysis of RCT data)	Not defined	Delivery data were collected by study physicians who reviewed the medical record	At birth, and up to 1 month	Cleft lip and palate, Polydactyly, and Other	
Ndyomugyenyi, 2008 (107)	RCT	Not defined	Physical examination	At birth, and up to 1 month	talipes equinovarus of the right foot and talipes equinovarus of the left foot, cleft palate, and multiple fingers	Used the overall population total in the analysis (versus placebo arm only)
Harrison, 2007 (54)	RCT	Not defined	Records review?	At delivery	Details not reported.	
Makanani, 2018 (82)	Cohort (secondary analysis of RCT data)	Structural anomalies only	Infant congenital anomalies identified at the time of delivery were determined by participant report and medical record review, when available.	At or around delivery	Umbilical hernia that was reducible, micrognathia and epicanthic folds, inguinal hernia that was repaired, craniofacial (structural) right frontal skull depression positional plagiocephaly, reducible umbilical hernia, umbilical hernia, and polydactyly	ASPIRE trial; While anomalies were reported by study arm, we included all congenital anomalies (overall) in our analysis. Prevalence was similar in both arms.

Taha, 2018 (134)	Cohort	Not defined	Physical examination	At delivery, potentially later follow-up	Not reported	PROMOTE Study

Table D.7. Congenital Anomaly Meta-Analyses Outliers

Manuscript	Country	Frequency	Definition	Outcome Ascertainment	Notes
Makanani_2018 (82)	Malawi, South Africa, Uganda, Zimbabwe	7.5%	All were structural anomalies (no further definition)	Congenital anomalies were identified at the time of delivery by participant report and medical records review, when available	ASPIRE study. Similar prevalence in placebo and dapivirine vaginal ring arm

SECTION E: LOW BIRTH WEIGHT

Table E.1. Search Result Flow

	N
**Title Review**	791
**Abstract Review**	213
**Manuscripts Reviewed**[*]	140
**Manuscripts Included From Main Search**[†]	115
**Manuscripts Added From Other Searches**[‡]	60
**Total Manuscripts Abstracted**	175
**Total Manuscripts Included For Analysis - 042 Countries Only**[§]	64

[*] Does not include number of references from systematic reviews reviewed.
[†] Systematic review reference review was not completed.
[‡] Some of these include duplicate populations with the studies that were abstracted through the main search. There will be removed at analysis.
[§] Kimani_2016 (69) reported prevalence estimates for two study countries and Taha_2018 (134) reported estimates for four countries. These studies were counted as one manuscript each, but provided multiple prevalence estimates in the analysis phase.

Table E.2. Pooled Prevalence of Low Birth Weight – Overall, by MTN-042 Country, and by HIV status

	# of estimates	# of pregnancies/ infants	Mean (Min, Max)	Median (IQR)	Pooled Prevalence (95%CI)	I^2 (p-value)
**Study Countries**	68	117,578	12.0% (2.8, 37.1)	11.3% (8.7, 14.4)	11.7% (10.6, 12.9)	97.1% (<0.01)
**Malawi**	17	14,827	10.6% (2.8, 21.2)	11.8% (7.1, 13.3)	10.4% (8.5, 12.5)	93.0% (<0.01)
**South Africa**	20	54,144	12.8% (5.0, 24.8)	12.6% (9.2, 15.1)	12.7% (10.9, 14.5)	96.8% (<0.01)
**Uganda**	20	19,760	12.3% (3.7, 37.1)	10.1% (9.2, 13.5)	11.9% (9.8, 14.2)	94.4% (<0.01)
**Zimbabwe**	11	28,847	12.0% (3.2, 22.5)	11.4% (8.0, 16.7)	11.8% (8.0, 16.2)	98.7% (<0.01)
**Living with HIV**	18	17,181	13.1% (3.2, 22.4)	14.1% (7.4, 17.8)	13.7% (11.2, 16.3)	94.5% (<0.01)
**HIV negative**	10	20,529	10.4% (3.0, 14.3)	11.1% (9.4, 13.5)	10.0% (7.7, 12.5)	95.1% (<0.01)

Figure E.1. Forest Plot Summarizing the Pooled Prevalence of Low Birth Weight– Malawi

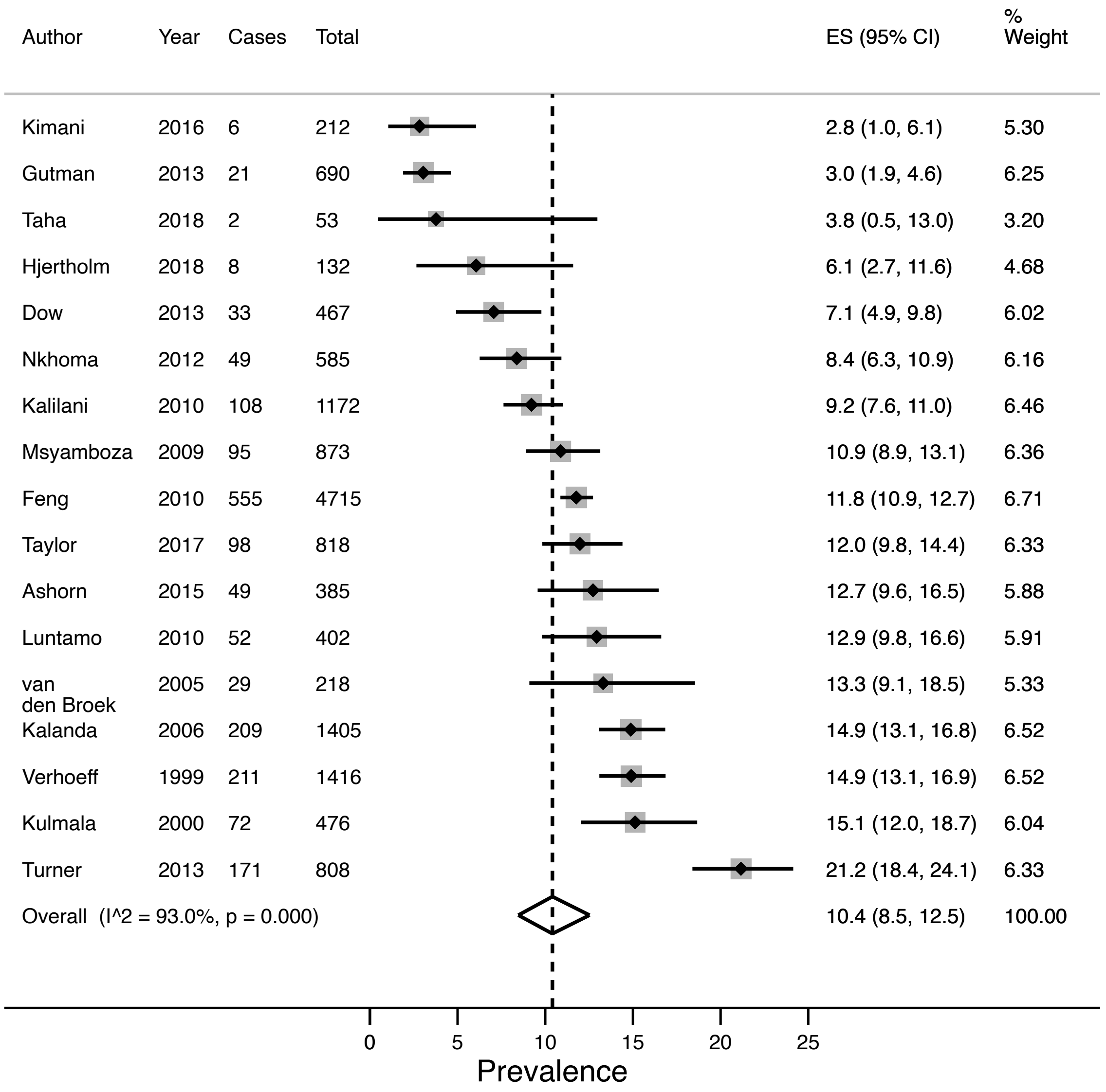

Figure E.2. Forest Plot Summarizing the Pooled Prevalence of Low Birth Weight - South Africa

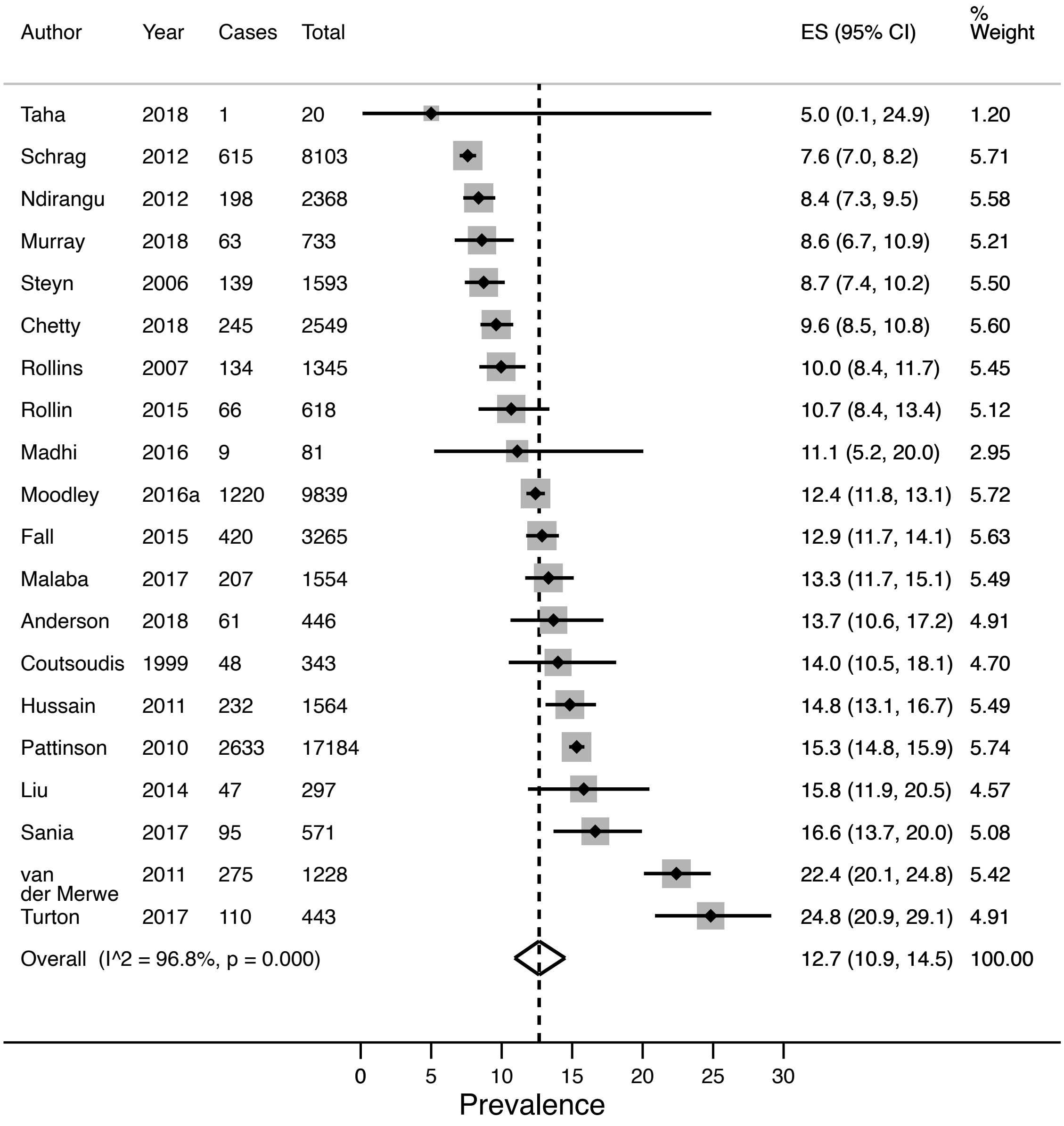

Figure E.3. Forest Plot Summarizing the Pooled Prevalence of Low Birth Weight – Uganda

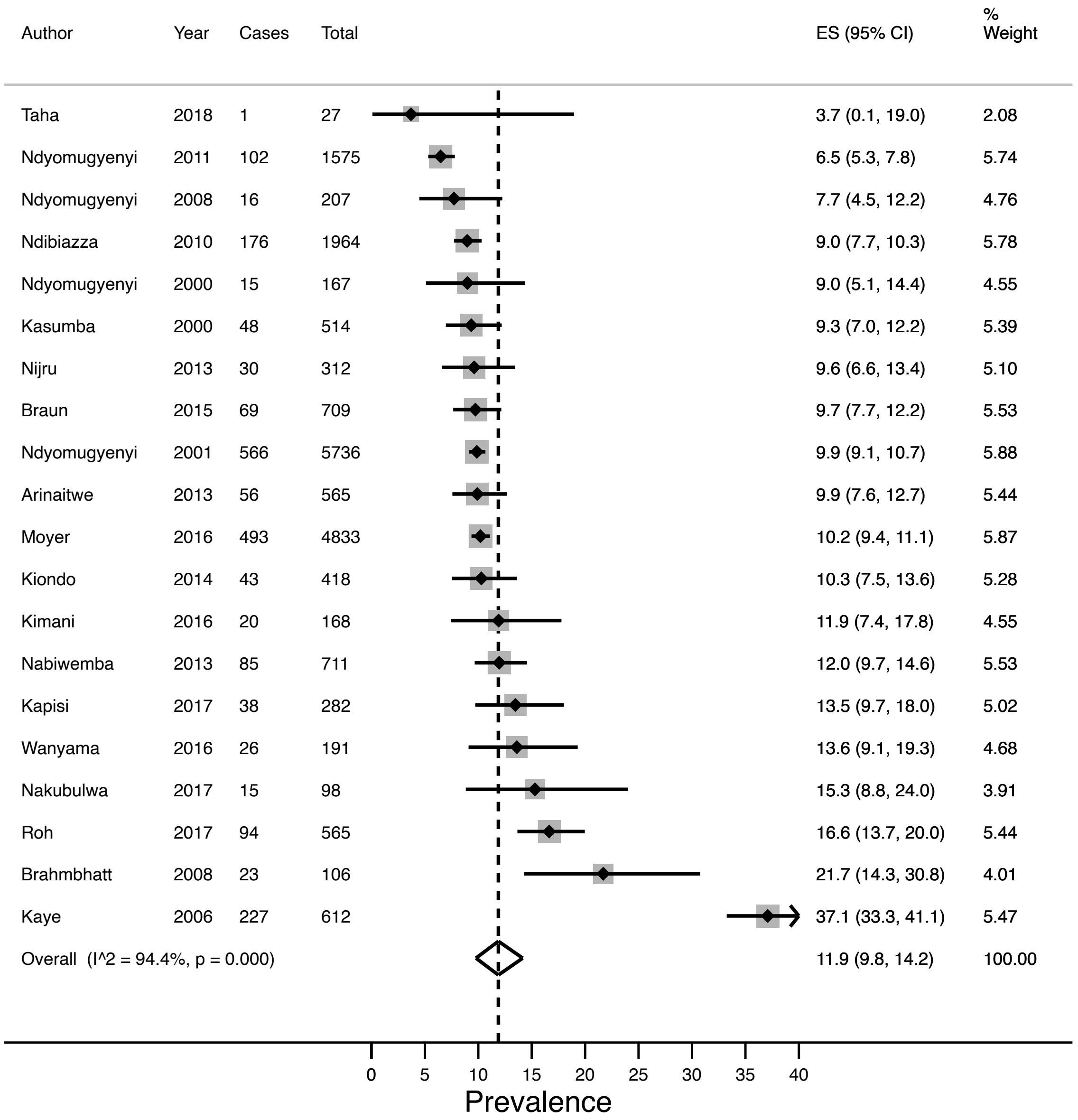

Figure E.4. Forest Plot Summarizing the Pooled Prevalence of Low Birth Weight – Zimbabwe

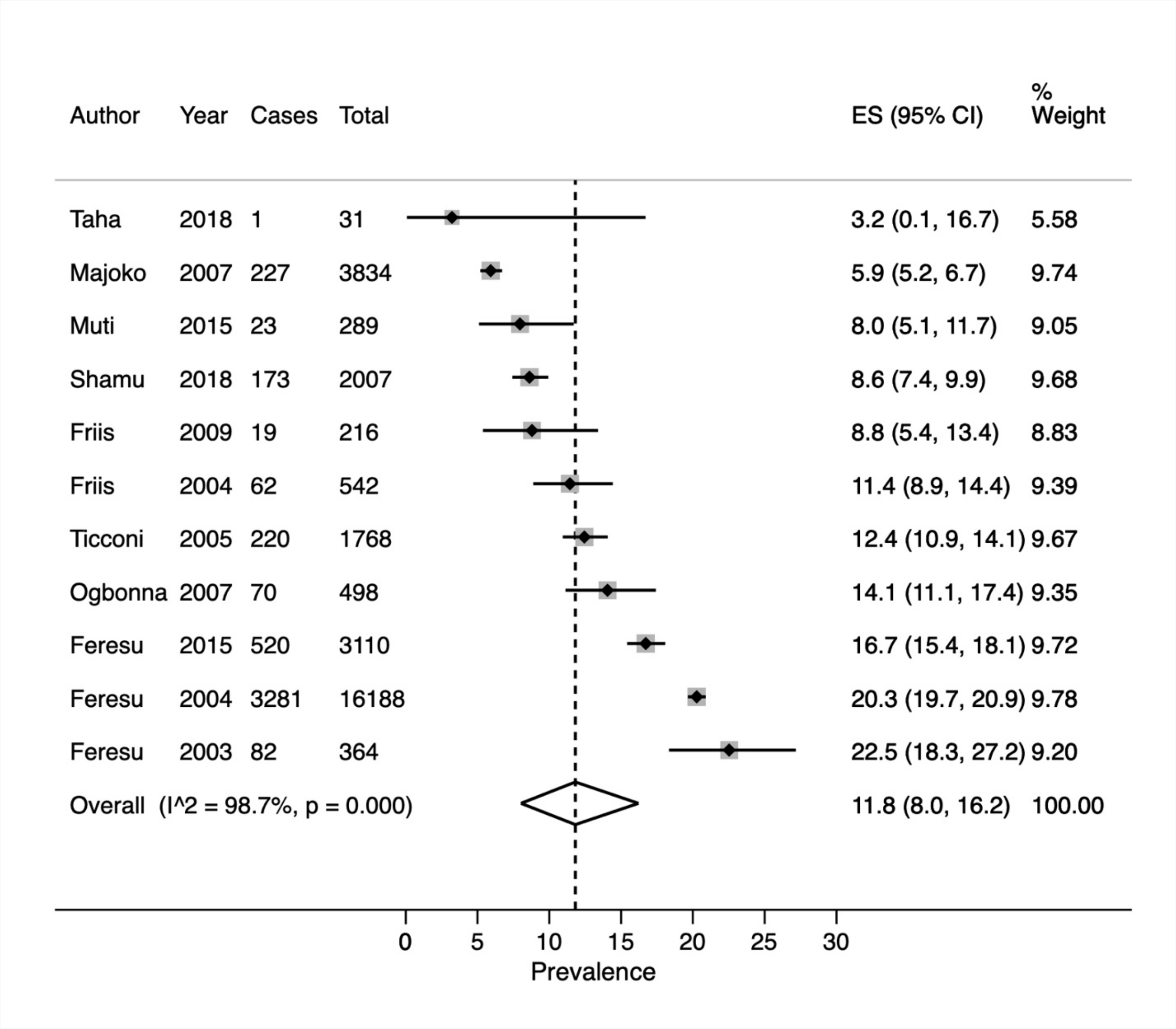

Figure E.5. Forest Plot Summarizing the Pooled Prevalence of Low Birth Weight - Among Women Living with HIV

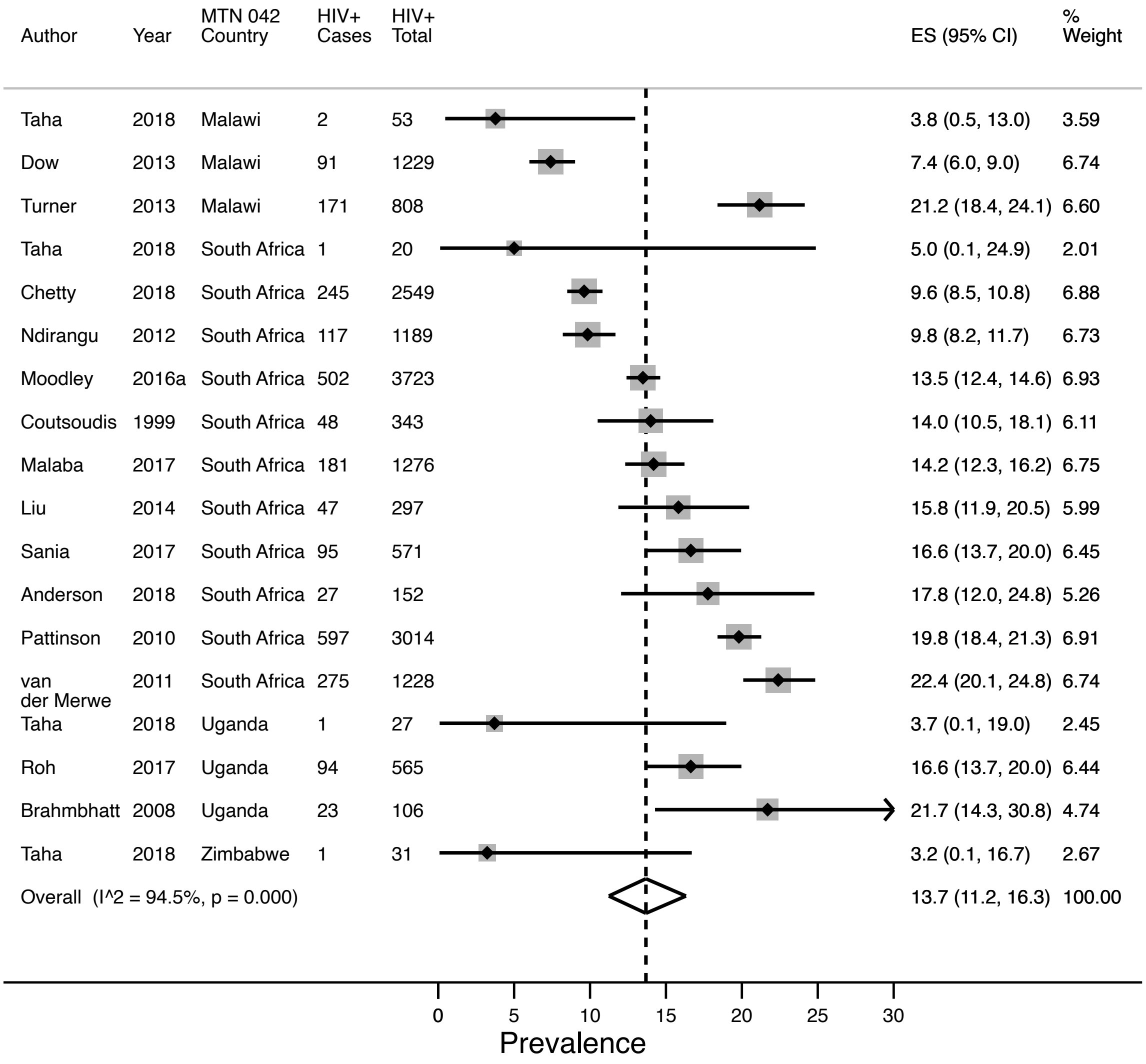

Figure E.6. Forest Plot Summarizing the Pooled Prevalence of Low Birth Weight Among HIV Negative Women

Author	Year	MTN 042 Country	HIV- Cases	HIV- Total	ES (95% CI)	% Weight
Gutman	2013	Malawi	21	690	3.0 (1.9, 4.6)	10.82
Ndirangu	2012	South Africa	81	1179	6.9 (5.5, 8.5)	11.27
Malaba	2017	South Africa	26	278	9.4 (6.2, 13.4)	9.49
Moodley	2016a	South Africa	651	5916	11.0 (10.2, 11.8)	11.82
Madhi	2016	South Africa	9	81	11.1 (5.2, 20.0)	6.33
Anderson	2018	South Africa	34	294	11.6 (8.1, 15.8)	9.60
Pattinson	2010	South Africa	1581	11053	14.3 (13.7, 15.0)	11.88
Arinaitwe	2013	Uganda	56	565	9.9 (7.6, 12.7)	10.60
Kapisi	2017	Uganda	38	282	13.5 (9.7, 18.0)	9.52
Wanyama	2016	Uganda	26	191	13.6 (9.1, 19.3)	8.67
Overall (I^2 = 95.1%, p = 0.000)					10.0 (7.7, 12.5)	100.00

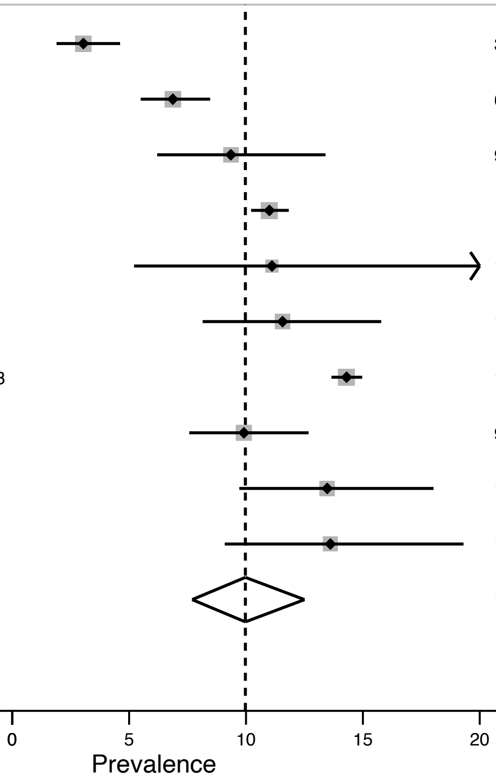

Table E.3. Sensitivity Analysis Including Studies with Live Birth Only: Pooled Prevalence of Low Birth Weight – Overall, by MTN 042 Country, and by HIV Status

	# of estimates*	# of pregnancies/ infants	Mean (Min, Max)	Median (IQR)	Pooled Prevalence (95%CI)	I^2 (p-value)
**Study Countries**	34	53,021	13.4% (2.8, 37.1)	13.1% (9.3, 15.3)	12.9% (10.9, 14.9)	97.6% (<0.01)
**Malawi**	7	5,392	12.1% (2.8, 21.2)	14.9% (3.0, 15.1)	11.3% (6.9, 16.5)	96.7% (<0.01)
**South Africa**	11	14,774	13.4% (8.4, 24.8)	13.3% (9.6, 15.8)	12.9% (10.8, 15.1)	92.8% (<0.01)
**Uganda**	11	10,688	12.9% (6.5, 37.1)	9.9% (9.0, 13.6)	12.2% (8.4, 16.6)	96.7% (<0.01)
**Zimbabwe**	5	22,167	16.4% (8.6, 22.5)	16.7% (14.1, 20.3)	16.0% (11.4, 21.3)	98.2% (<0.01)
**Living with HIV**	8	7,185	14.9% (9.6, 21.2)	15.0% (11.9, 17.2)	14.5% (11.5, 17.8)	92.4% (<0.1)
**HIV negative**	5	2,632	8.9% (3.0, 13.6)	9.4% (6.9, 11.6)	8.2% (4.9, 12.3)	90.6% (<0.01)

*Included studies: Anderson_2018(5), Ashorn_2015 (7), Braun_2015 (20), Chetty_2018 (24), Coutsoudis_1999 (28), Fall_2015 (34), Feresu_2003 (37), Feresu_2004 (38), Feresu_2015 (40), Gutman_2013 (52), Kalanda_2006 (59), Kasumba_2000 (65), Kaye_2006(67), Kimani_2016 (69), Kulmala_2000 (72), Liu_2014 (77), Malaba_2017 (83), Nabiwemba_2013 (96), Nakubulwa_2017 (99), Ndirangu_2012 (104), Ndyomugyenyi_2000 (105), Ndyomugyenyi_2001 (106), Ndyomugyenyi_2008 (107), Ndyomugyenyi_2011 (108), Ogbonna_2007 (115), Rollins_2007 (122), Sania_2017 (123), Shamu_2018 (129), Steyn_2006 (132), Turner_2013 (140), Turton_2017 (141), Verhoeff_1999 (146), Wanyama_2016 (150)

Table E.4. Sensitivity Analysis Excluding Outliers: Pooled Prevalence of Low Birthweight – Overall, by MTN-042 Country, and by HIV status

	# of estimates*	# of pregnancies/ infants	Mean (Min, Max)	Median (IQR)	Pooled Prevalence (95%CI)	I^2 (p-value)
**Study Countries**	66	116,523	11.4% (2.8, 22.5)	11.0% (8.6, 14.0)	11.3% (10.2, 12.4)	96.8% (<0.01)
**Malawi**	17	14,827	10.6% (2.8, 21.2)	11.8% (7.1, 13.3)	10.4% (8.5, 12.5)	93.0% (<0.01)
**South Africa**	19	53,701	12.2% (5.0, 22.4)	12.4% (8.7, 14.8)	12.1% (10.4, 13.9)	96.7% (<0.01)
**Uganda**	19	19,148	11.0% (3.7, 21.7)	9.9% (9.0, 13.5)	10.5% (9.4, 11.6)	76.8% (<0.01)
**Zimbabwe**	11	28,847	12.0% (3.2, 22.5)	11.4% (8.0, 16.7)	11.8% (8.0, 16.2)	98.7% (<0.01)
**Living with HIV**	18	17,181	13.1% (3.2, 22.4)	14.1% (7.4, 17.8)	13.7% (11.2, 16.3)	94.5% (<0.01)
**HIV negative**	10	20,529	10.4% (3.0, 14.3)	11.1% (9.4, 13.5)	10.0% (7.7, 12.5)	95.1% (<0.01)

*N=2 studies had a prevalence estimate that was greater than 1.5 times the interquartile range (IQR) of all included studies. The excluded studies are described in Supplemental Table E.5

Table E.5. Low Birthweight Meta-Analysis Outliers

Manuscript	Country	Frequency	Definition	Outcome Ascertainment	Notes
Turton_2017(141)	South Africa	24.8%	<2500g	Abstraction from maternal hospital record	The aim of this study was to investigate the oral health status and pregnancy outcomes of mothers attending an antenatal clinic in South Africa in order to assess periodontal disease as an independent risk factor for low birthweight. "The exclusion criteria were a history of medical problems that may have affected the study outcome, such as current use of systemic corticosteroids and/or antibiotics, existing heart disease, hypertension, diabetes, asthma, blood-cell disorders and chronic renal disease. Also excluded were mothers with induced labour, multiple pregnancies, smokers and those who used or abused alcohol."
Kaye_2006 (67)	Uganda	37.1%	<2500g	At time of delivery, participant interview by research assistant	Enrolled women in second trimester (excluded women with chronic disease associated with frequent hospitalization or likely to cause LBW)

SECTION F: NEONATAL MORTALITY

Table F.1. Search Result Flow

	N
**Title Review**	834
**Abstract Review**	379
**Manuscripts Reviewed**[*]	211
**Manuscripts Included From Main Search**[†]	100
**Manuscripts Added From Other Searches**[‡]	41
**Total Manuscripts Abstracted**	141
**Total Manuscripts Included For Neonatal Mortality Analysis – MTN-042 Countries Only**[§]	26

[*] Does not include number of references from systematic reviews reviewed.
[†] Includes systematic review reference reviews.
[‡] Some of these include duplicate populations with the studies that were abstracted through the main search. These were removed at analysis.
[§] Excludes duplicate populations, studies with incorrect definitions of the outcomes, studies that only reported early neonatal mortality. Definitions accepted for neonatal mortality meta-analysis includes deaths occurring within 28 days, before 29 days, within one month, and before 30 days.

Table F.2. Pooled Prevalence of Neonatal Mortality – Overall, by MTN-042 Country, and by HIV status

	# of estimates*	# of pregnancies/ infants	Mean (Min, Max)	Median (IQR)	Pooled Prevalence (95% CI)	I^2 (p-value)
**Study Countries**	26	342,853	2.0% (0.3, 4.2)	1.9% (0.7, 3.0)	1.7% (1.4, 2.1)	97.2% (<0.01)
**Malawi**	8	22,030	2.7% (0.5, 4.2)	3.2% (1.3, 3.9)	2.4% (1.4, 3.5)	94.8% (<0.01)
**South Africa**	10	276,251	1.0% (0.3, 2.5)	0.7% (0.6, 1.3)	0.9% (0.6, 1.2)	95.4% (<0.01)
**Uganda**	7	40,616	2.7% (2.0, 3.7)	2.7% (2.2, 3.4)	2.6% (2.3, 3.0)	38.7% (0.1)
**Zimbabwe**	1	3,956	1.3% -	-	1.3% (0.9, 1.7)	-
**Living with HIV**	5	6,713	1.3% (0.3, 3.7)	1.0% (0.5,1.1)	1.0% (0.5, 1.8)	81.2% (<0.01)
**HIV negative**	1	11,053	0.5% -	-	0.5% (0.4, 0.6)	-

* No outliers were identified so no sensitivity analysis excluding outliers was conducted.

Figure F.1. Forest Plot Summarizing the Pooled Prevalence of Neonatal Mortality – Overall and by Country

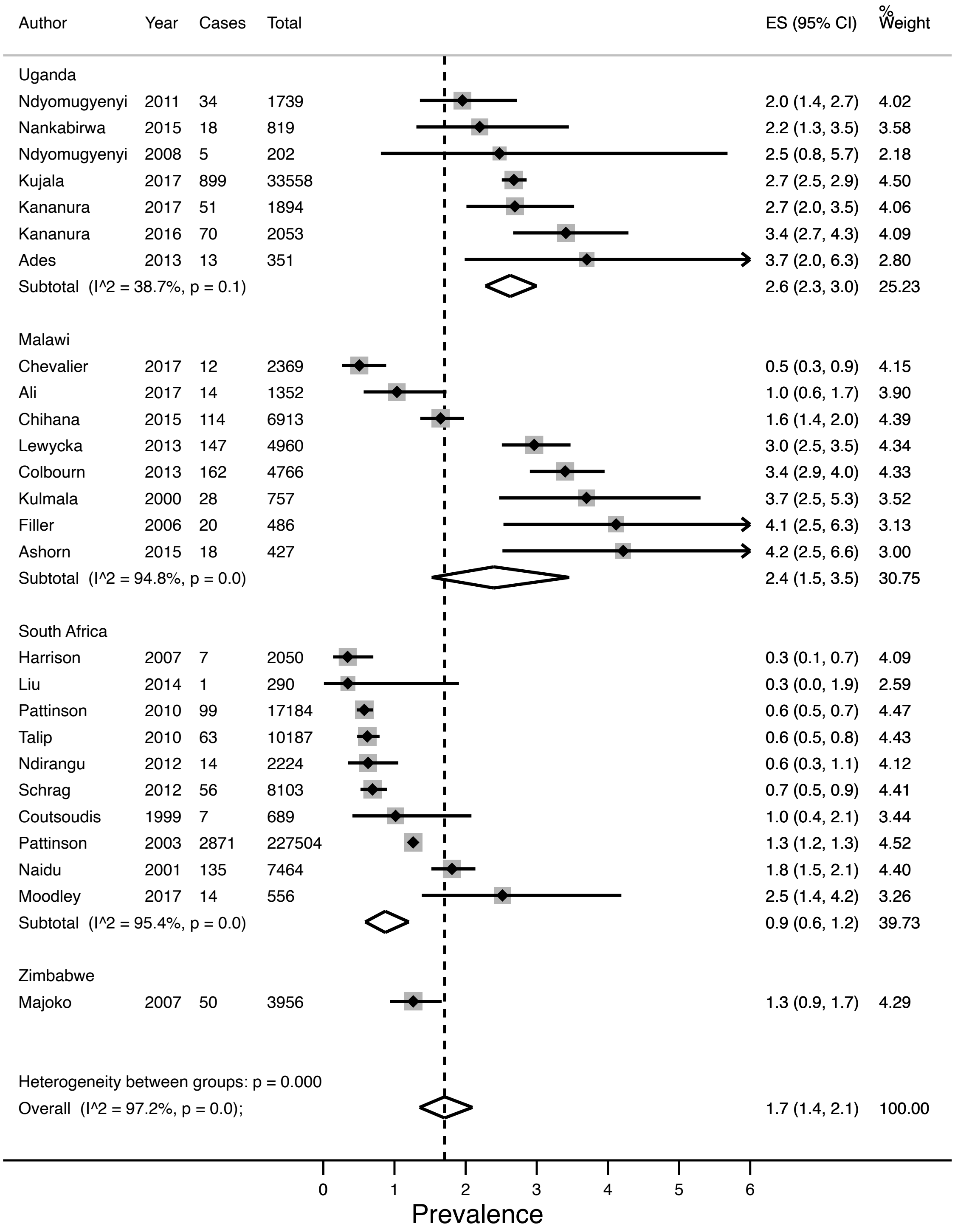

Figure F.2. Forest Plot Summarizing the Pooled Prevalence of Neonatal Mortality Among Women Living with HIV

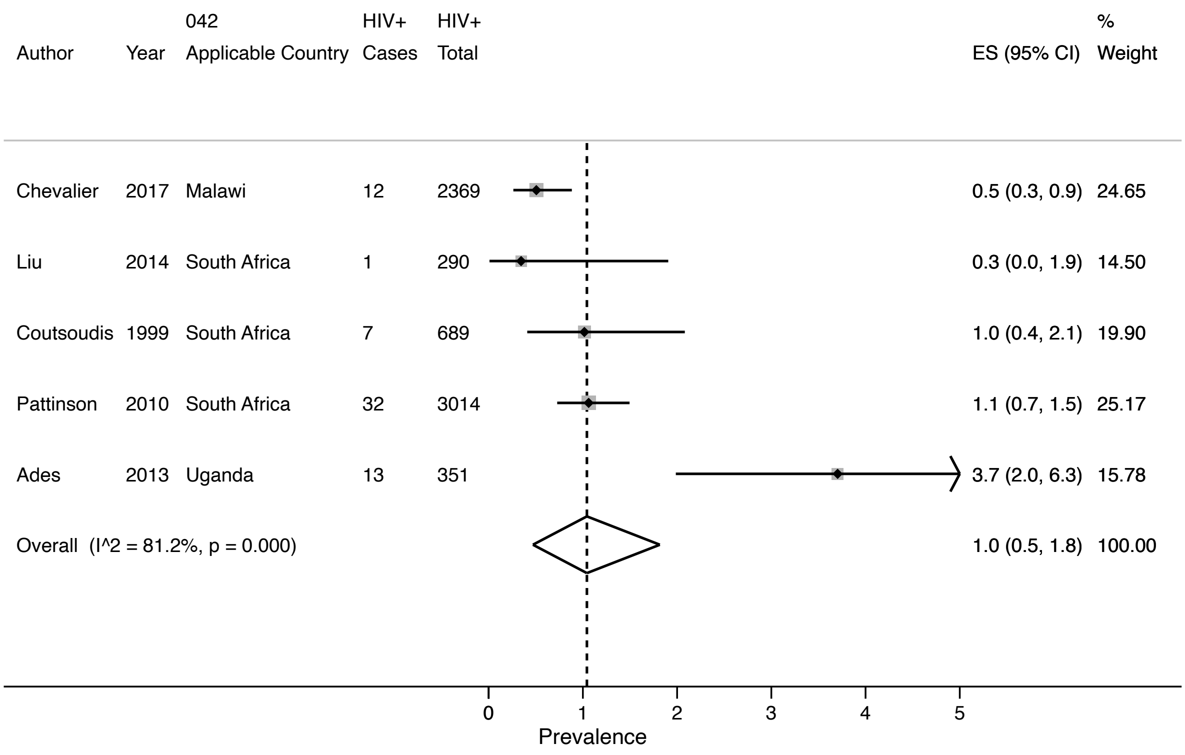

Note: No forest plot was generated for HIV negative women as there was only one study reporting a prevalence estimate.

Table F.3. Neonatal Mortality (<28 days) Summary - Sensitivity analysis excluding studies that did not define “neonatal mortality”

	# of estimates*	# of pregnancies/ infants	Mean (Min, Max)	Median (IQR)	Pooled Prevalence (95%CI)	I^2 (p-value)
**Study Countries**	19	73,952	2.3% (0.3, 4.2)	2.5% (1.0, 3.4)	2.0% (1.5, 2.6)	95.2% (<0.01)
**Malawi**	8	22,030	2.7% (0.5, 4.2)	3.2% (1.3, 3.9)	2.4% (1.5, 3.5)	94.8% (<0.01)
**South Africa**	4	11,306	0.7% (0.3, 1.0)	0.7% (0.5, 0.9)	0.6% (0.5, 0.8)	0.0% (0.7)
**Uganda**	7	40,616	2.7% (2.0, 3.7)	2.7% (2.2, 3.4)	2.6% (2.3, 3.0)	38.7% (0.1)
**Zimbabwe**	0	-	-	-	-	-
**Living with HIV**	4	3,699	1.4% (0.3, 3.7)	0.8% (0.4, 2.4)	1.1% (0.3, 2.4)	75.0% (<0.01)
**HIV negative**	0	-	-	-	-	-

* Included studies: Ades_2013 (2), Ali_2017 (3), Ashorn_2015 (7), Chevalier_2017 (25), Chihanna_2015 (26), Colbourn_2013 (27), Coutsoudis_1999 (28), Filler_2006 (41), Kananura_2016 (62), Kananura_2017 (63), Kujala_2017 (71), Kulmala_2000 (72), Lewycka_2013 (76), Liu_2014 (77), Nankabirwa_2015 (101), Ndirangu_2012 (104), Ndyomugyenyi_2008 (107), Ndyomugyenyi_2011 (108), Schrag_2012 (125)

Notes F.1. Neonatal Mortality Study Specific Notes:

- RCTs where overall proportion (not control prevalence) was used:
    - Filler 2006 was an RCT testing monthly SP vs 2 dose SP during pregnancy in women living with HIV and HIV negative women. The n was not presented by arm or HIV status, just proportions. The analysis therefore included the combined (intervention + control) prevalence estimate.
- Pattinson_2010
    - Used neonatal mortality rates and N for HIV subgroups to calculate group specific prevalence; the total number of deaths by calculation (n=99) is smaller than a number reported in the paper (103). Used 99 as the numerator and included the paper since the prevalence difference using 99 vs 103 was insignificant given the total denominator at risk n=17184).

SECTION G: CHORIOAMNIONITIS

Table G.1. Search Result Flow

	N
**Title Review**	6
**Abstract Review**	1
**Manuscripts Reviewed**	1
**Manuscripts Included From Main Search***	1
**Manuscripts Added From Other Searches**	5
**Total Manuscripts Abstracted†**	6
**Total Manuscripts Included For Analysis‡**	6

* Does not include number of references from systematic reviews reviewed
† Includes systematic review reference reviews
‡ Includes studies from non-MTN042 countries

Table G.2. Pooled Prevalence of Chorioamnionitis – Overall, by MTN-042 Country, and by HIV status

	# of estimates	# of pregnancies/ infants	Mean (Min, Max)	Median (IQR)	Pooled Prevalence (95% CI)	I^2 (p-value)
**Total**	6	2,086	17.6% (4.7, 36.0)	13.2% (8.1, 30.6)	16.2% (8.0, 26.7)	96.9% (<0.01)
**Study Countries**	3	1,099	26.6% (13.1, 36.0)	30.6% (13.1, 36.0)	25.9% (14.0, 39.8)	-
**Malawi**	1	676	30.6% -	-	30.6% (27.2, 34.2)	-
**South Africa**	0	-	-	-	-	-
**Uganda**	2	4,23	24.6% (13.1, 36.0)	24.6% (13.1, 36.0)	21.5% (17.7, 25.6)	-
**Zimbabwe**	0	-	-	-	-	-
**Total - Living with HIV**	4	1,171	20.4% (4.7, 36.0)	20.4% (7.5, 33.3)	18.7% (5.6, 36.9)	97.5% (<0.01)
**Total - HIV Negative**	1	68	5.9% -	-	5.9% (1.3, 13.0)	-

Figure G.1. Forest Plot Summarizing the Pooled Prevalence of Chorioamnionitis

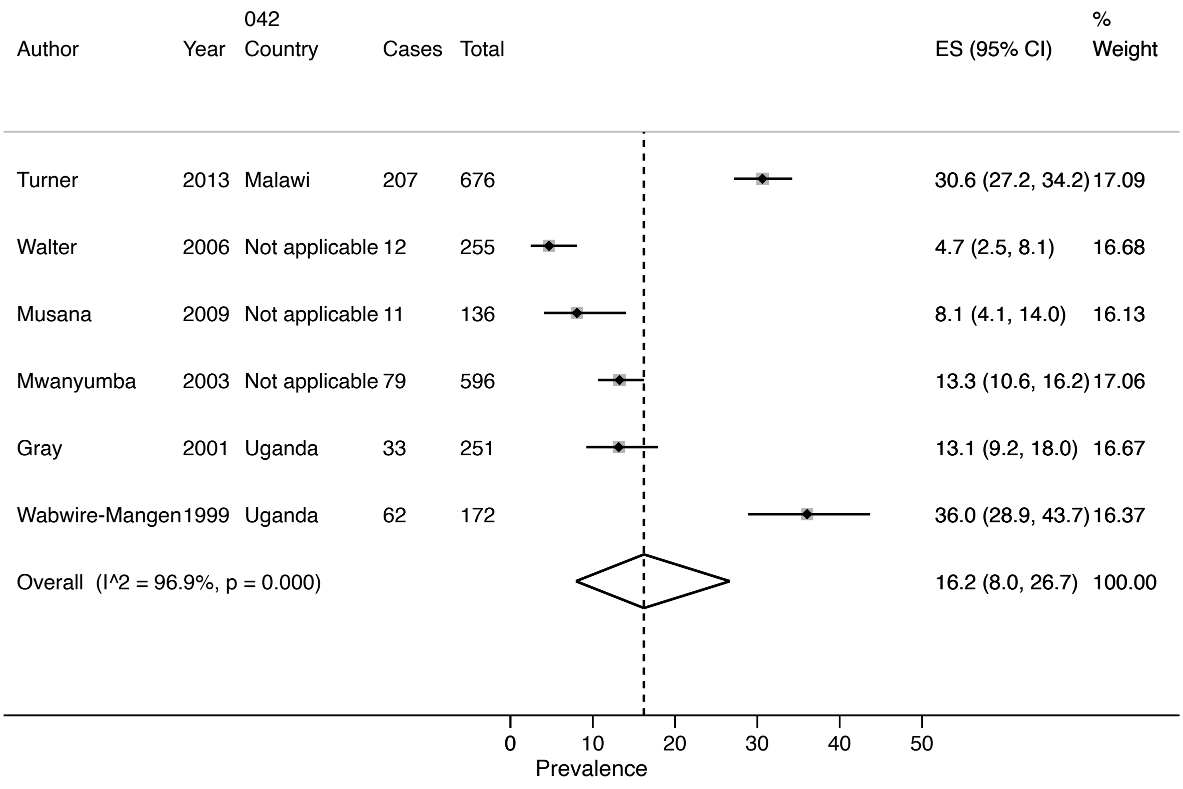

Notes G.1. Chorioamnionitis Study Specific Notes

- Definitions of Chorioamnionitis
  - Clinical symptom: Walter_2006 (149)
  - Histopathology: Turner_2013 (140), Gray_2001 (49), Mwanyumba_2003 (94), Wabwire-Mangen_1999 (148)
  - Undefined: Musana_2009 (92)
- Studies that included women living with HIV with low CD4 count or advanced AIDS (WHO 3 or 4)
  - Turner_2013 (140)
    - Malawi. All were ART naive at enrollment, but 95% received single dose nevirapine at delivery.
  - Walter_2006 (149)
    - Zambia. All had low CD4 and excluded women who began taking ART while pregnant.
  - Musana_2009 (92)
    - Kenya. Included women Living with HIV with advanced disease.
  - Wabwire-Mangen_1999 (148)
    - Uganda. Study was conducted in 1990-1992, pre-ART.

SECTION H: ENDOMETRITIS

Table H.1. Search Result Flow

	N
**Title Review**	43
**Abstract Review**	12
**Manuscripts Reviewed**[*]	4
**Manuscripts Included From Main Search**[†]	4
**Manuscripts Added From Other Searches**	4
**Total Manuscripts Abstracted**	8
**Total Manuscripts Included For Analysis**	7

[*] Does not include number of references from systematic reviews reviewed.
[†] Includes systematic review reference reviews.

Table H.2. Pooled Prevalence of Endometritis – Overall, by MTN-042 Country, and by HIV status

	# of estimates*	# of pregnancies/ infants	Mean (Min, Max)	Median (IQR)	Pooled Prevalence (95% CI)	I^2 (p-value)
**Total**	7	12,653	5.5% (0.0, 19.8)	1.8% (0.3, 13.6)	3.3% (1.1, 6.6)	98.4% (p<0.01)
**Study Countries**	5	11,416	3.3% (0.0, 13.6)	0.8% (0.3, 1.8)	1.6% (0.4, 3.4)	96.7% (p<0.01)
**Malawi**	1	2,791	0.8% -	-	0.8% (0.5, 1.2)	-
**South Africa**	2	4,197	6.9% (0.3, 13.6)	6.9% (0.3, 13.6	0.3% (0.2, 0.6)	-
**Uganda**	2	4,428	0.9% (0.0, 1.8)	0.9% (0, 1.8)	1.6% (0.4, 3.4)	-
**Zimbabwe**	0	-	-	-	-	-
**Total - Living with HIV**	3	4,022	5.0% (0.5, 13.6)	0.8% (0.5, 13.6)	2.9% (0.3, 7.6)	96.9% (p<0.01)
**Total - HIV Negative**	1	2, 916	0.2% -	-	0.2% (0.1, 0.4)	-

[*] No outliers were identified so no sensitivity analysis excluding outliers was conducted.

Figure H.1. Forest Plot Summarizing the Pooled Prevalence of Endometritis

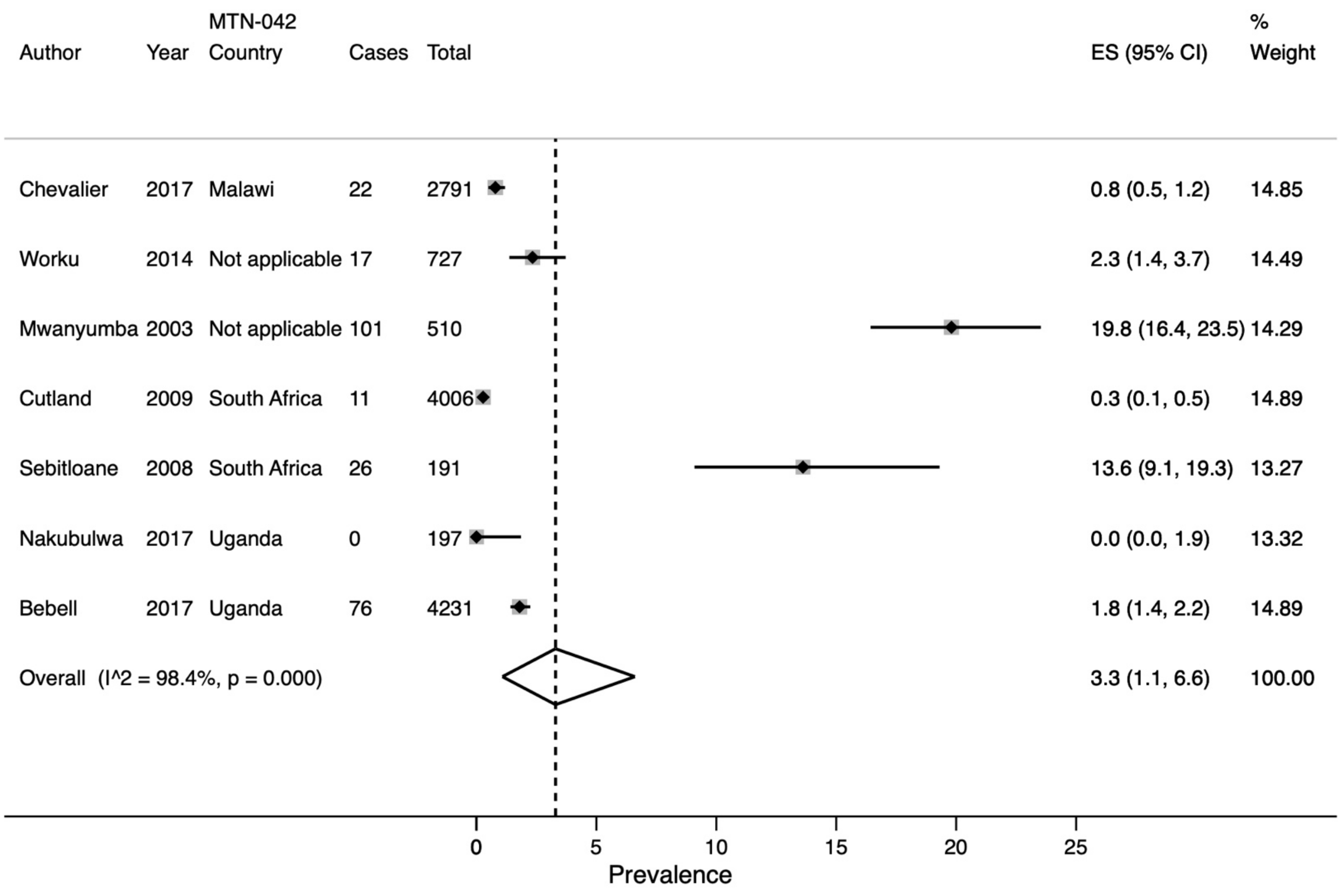

SECTION I: POSTPARTUM HEMORRHAGE

Table I.1. Search Result Flow

	N
**Title Review**	217
**Abstract Review**	68
**Manuscripts Reviewed***	32
**Manuscripts Included From Main Search†**	16
**Manuscripts Added From Other Searches**	17
**Total Manuscripts Abstracted for Antepartum and Postpartum Hemorrhage**	33
**Total Manuscripts Abstracted for Postpartum Hemorrhage only**	28
**Total Manuscripts Included For Postpartum Hemorrhage Analysis- MTN 042 Only‡**	17

* Does not include number of references from systematic reviews reviewed.
† Includes systematic review reference reviews

Table I.2. Pooled Prevalence of Postpartum Hemorrhage – Overall, by MTN-042 country, and by HIV status

	# of estimates*	# of pregnancies/ infants	Mean (Min, Max)	Median (IQR)	Pooled Prevalence (95%CI)	I^2 (p-value)
**Study Countries**	17	71,308	5.6% (0.6, 17.4)	3.1% (1.6, 9.0)	4.4% (3.0, 6.0)	98.7% (<0.01)
**Malawi**	2	5,875	2.1% (1.1, 3.1)	2.1% (1.1, 3.1)	2.0% (1.7, 2.4)	-
**South Africa**	10	57,046	4.7% (0.6, 15.8)	3.1% (0.7, 7.0)	3.6% (2.0, 5.6)	98.9% (<0.01)
**Uganda**	3	3,564	9.7% (2.8, 17.4)	9.0% (2.8, 17.4)	8.8% (2.5, 18.3)	-
**Zimbabwe**	2	4,823	7.4% (1.6, 13.3)	7.4% (1.6, 13.3)	1.9% (1.5, 2.3)	-
**Living with HIV**	5	7,541	8.2% (0.7, 33.3)	1.1% (0.7, 4.9)	4.5% (1.3, 9.4)	98.3% (<0.01)
**HIV negative**	4	11,650	7.1% (0.3, 21.4)	3.4% (1.5, 12.7)	5.2% (0.4, 14.0)	97.0% (<0.01)
**≥500mL lost†**	7	25,720	8.9% (1.6,17.4)	9.0% (2.5, 15.8)	7.5% (4.6, 11.1)	98.5% (<0.01)

* No outliers were identified so no sensitivity analysis excluding outliers was conducted.
† For the purpose of this sub-analysis analysis, any study reporting ≥500mL of blood lost independent of length of time or delivery type (vaginal, c-section) was included. Studies included: Beltman_2011 (15), Bloch_2015 (17), Abdel-Aleem_2010 (1), Ononge_2016 (116), Atukunda_2014 (9), Ngwenya_2016 (109), Kundodyiwa_2001 (74)

Figure I.1. Forest Plot Summarizing the Pooled Prevalence of Postpartum Hemorrhage

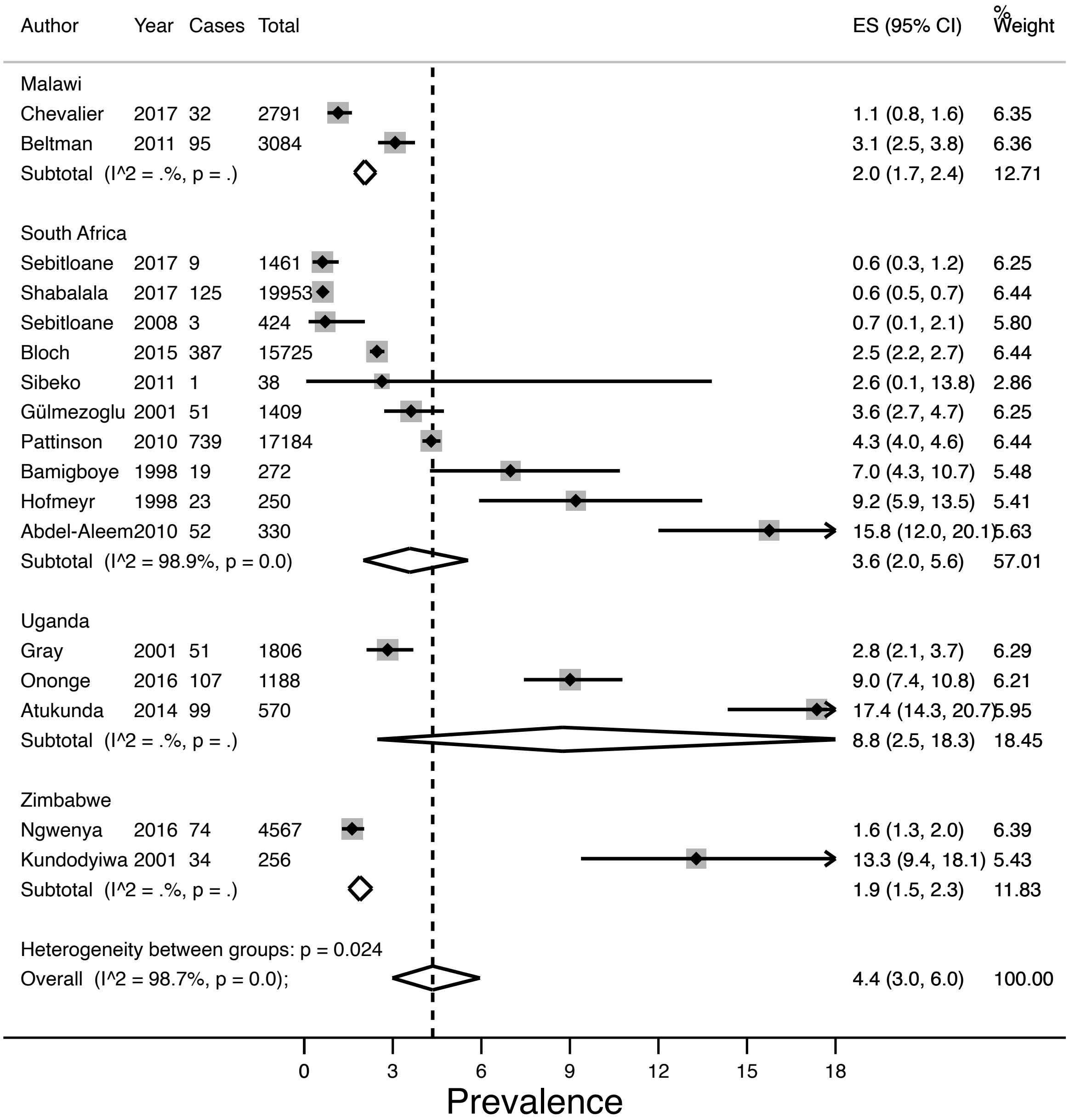

Figure I.2. Forest Plot Summarizing the Pooled Prevalence of Postpartum Hemorrhage Among Women Living with HIV

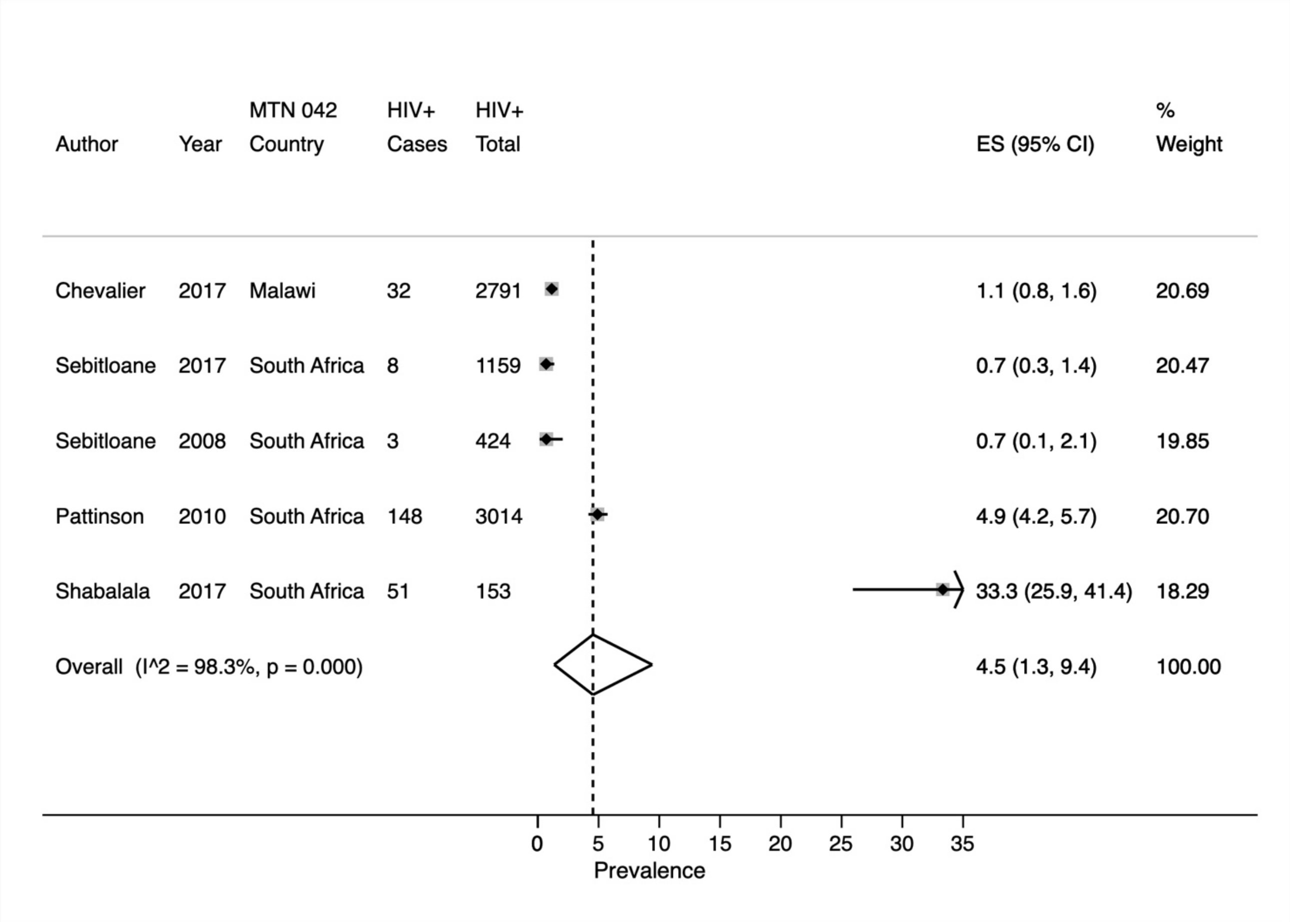

Figure I.3. Forest Plot Summarizing the Pooled Prevalence of Postpartum Hemorrhage Among HIV Negative Women

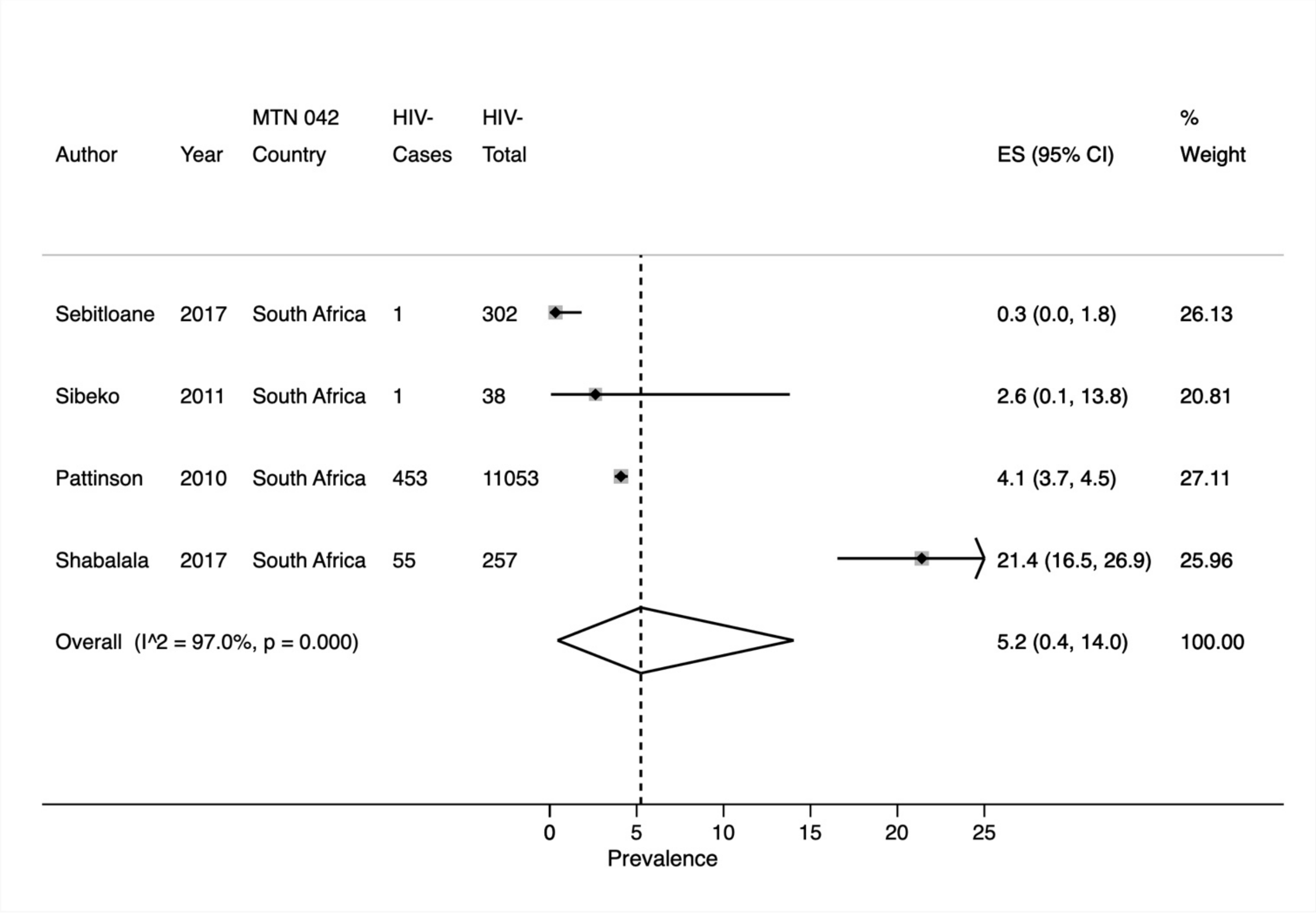

Figure I.4. Forest Plot Summarizing the Pooled Prevalence of Postpartum Hemorrhage - ≥500mL Blood Loss

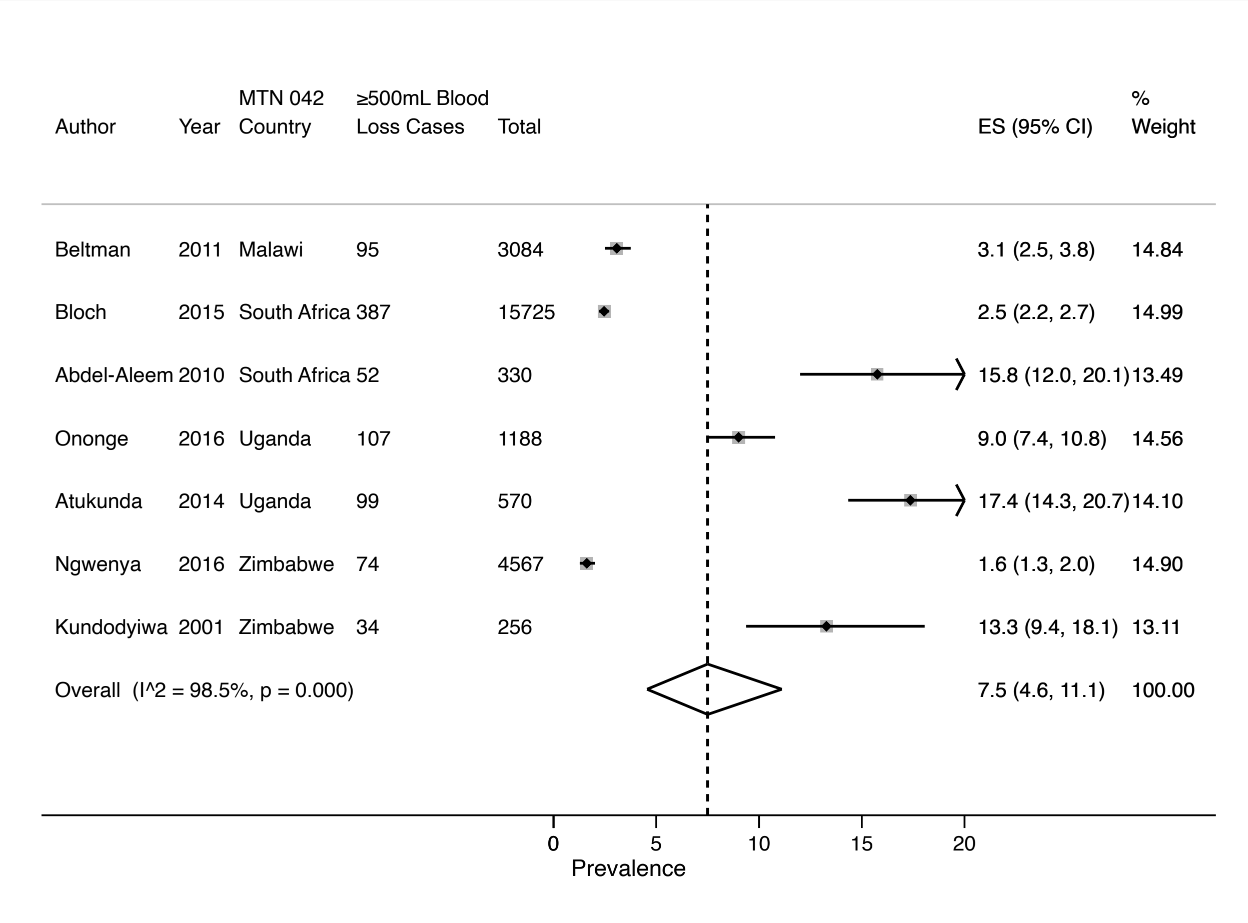

SECTION J: HYPERTENSIVE DISORDERS OF PREGNANCY

Table J.1. Search Result Flow

	N
**Title Review**	549
**Abstract Review**	124
**Manuscripts Reviewed***	54
**Manuscripts Included From Main Search†**	19
**Manuscripts Added From Other Searches**	33
**Total Manuscripts Abstracted**	52
**Total Manuscripts Included For MTN-042 Analysis**	
**Gestational Hypertension**	14
**Pre-eclampsia/Eclampsia**	9

* Does not include number of references from systematic reviews reviewed.
† Includes systematic review reference reviews.

GESTATIONAL HYPERTENSION

Table J.2. Pooled Prevalence of Gestational Hypertension – Overall, by MTN-042 Country and by HIV status

	# of estimates	# of pregnancies/ infants	Mean (Min, Max)	Median (IQR)	Pooled Prevalence (95%CI)	I^2 (p-value)
**Study Countries**	14	32,024	12.4% (0.7, 23.4)	12.3% (8.6, 15.4)	11.4% (7.8, 15.7)	99.1% (<0.01)
**Malawi**	0	-	-	-	-	-
**South Africa**	10	23,225	11.1% (0.7, 21.0)	12.3 % (6.7, 14.8)	10.0% (5.8, 15.3)	99.2% (<0.01)
**Uganda**	1	418	11.5% -	-	11.5% (8.6, 14.9)	-
**Zimbabwe**	3	8,381	17.1% (8.6, 23.4)	19.4% (8.6, 23.4)	16.5% (6.5, 29.9)	-
**Living with HIV**	3	3,201	11.1% (1.6, 17.0)	14.8% (1.6, 17.0)	9.6% (1.3, 24.3)	99.0% (<0.01)
**HIV Negative**	4	2,399	7.1% (0.7, 14.9)	6.5% (1.9, 12.4)	5.8% (0.9, 14.3)	97.7% (<0.01)

Figure J.1. Forest Plot Summarizing the Pooled Prevalence of Gestational Hypertension

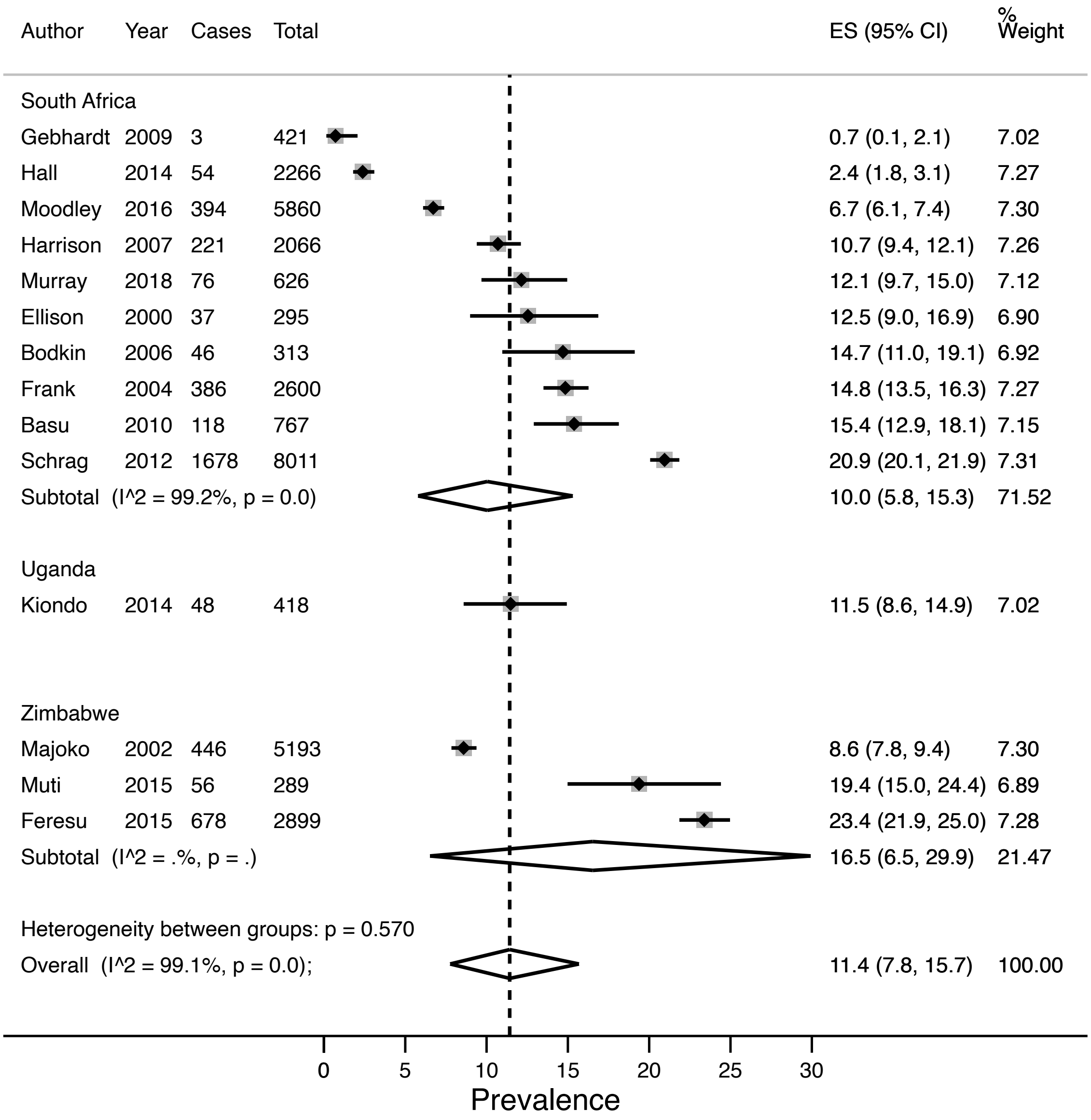

*Moodley 2016 refers to Moodley, 2016b (87)

Figure J.2. Forest Plot Summarizing the Pooled prevalence of Gestational Hypertension Among Women Living with HIV

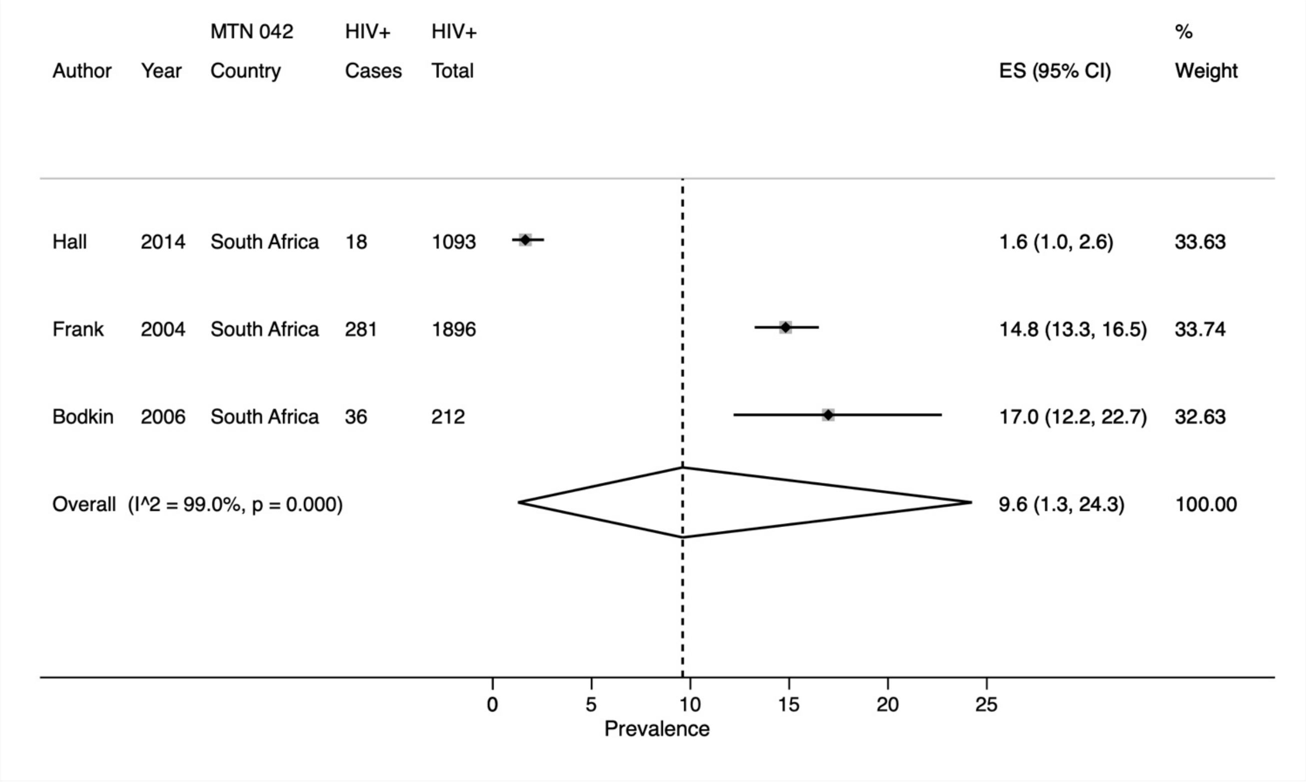

Figure J.3. Forest Plot Summarizing the Pooled prevalence of Gestational Hypertension Among HIV Negative Women

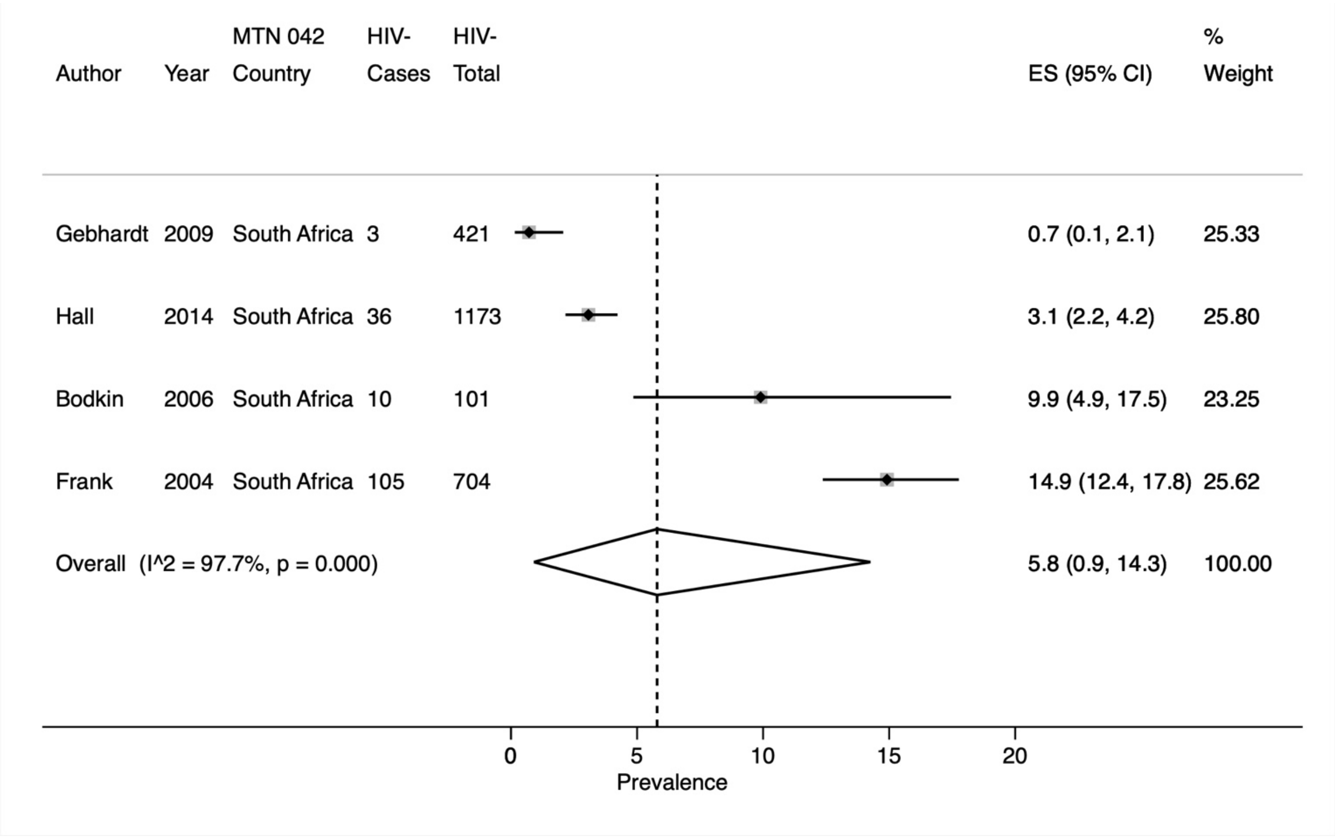

PRE-ECLAMPSIA/ECLAMPSIA

Table J.3. Pooled Prevalence of Preeclampsia/Eclampsia – Overall, by MTN-042 Country, and by HIV status

	# of estimates*	# of pregnancies/ infants	Mean (Min, Max)	Median (IQR)	Pooled Prevalence (95%CI)	I^2 (p-value)
**Study Countries**	9	50,234	4.5% (0.7, 10.7)	4.5% (2.1, 5.8)	4.0% (1.9, 6.8)	99.4% (<0.01)
**Malawi**	1	2,791	0.7% -	-	0.7% (0.4, 1.1)	-
**South Africa**	5	37,650	6.4% (2.8, 10.7)	5.8% (5.3, 7.6)	6.2% (3.0, 10.3)	99.5% (<0.01)
**Uganda**	1	418	4.5% -	-	4.5% (2.8, 7.0)	-
**Zimbabwe**	2	9,375	1.7 % (1.3, 2.1)	1.7 % (1.3, 2.1)	1.3% (1.1, 1.5)	-
**Living with HIV**	3	7,701	2.7% (0.7, 5.2)	2.1% (0.7, 5.2)	2.3% (0.6, 5.2)	97.9% (<0.01)
**HIV Negative**	3	4,910	5.5% (3.1, 7.6)	5.7% (3.1, 7.6)	5.2% (2.6, 8.4)	92.7% (<0.01)

*Only included studies reporting prevalence of both preeclampsia and eclampsia either as a composite or each separately. In addition, no outliers were identified so no sensitivity analysis excluding outliers was conducted.

Figure J.4. Forest Plot Summarizing the Pooled Prevalence of Pre-eclampsia/Eclampsia

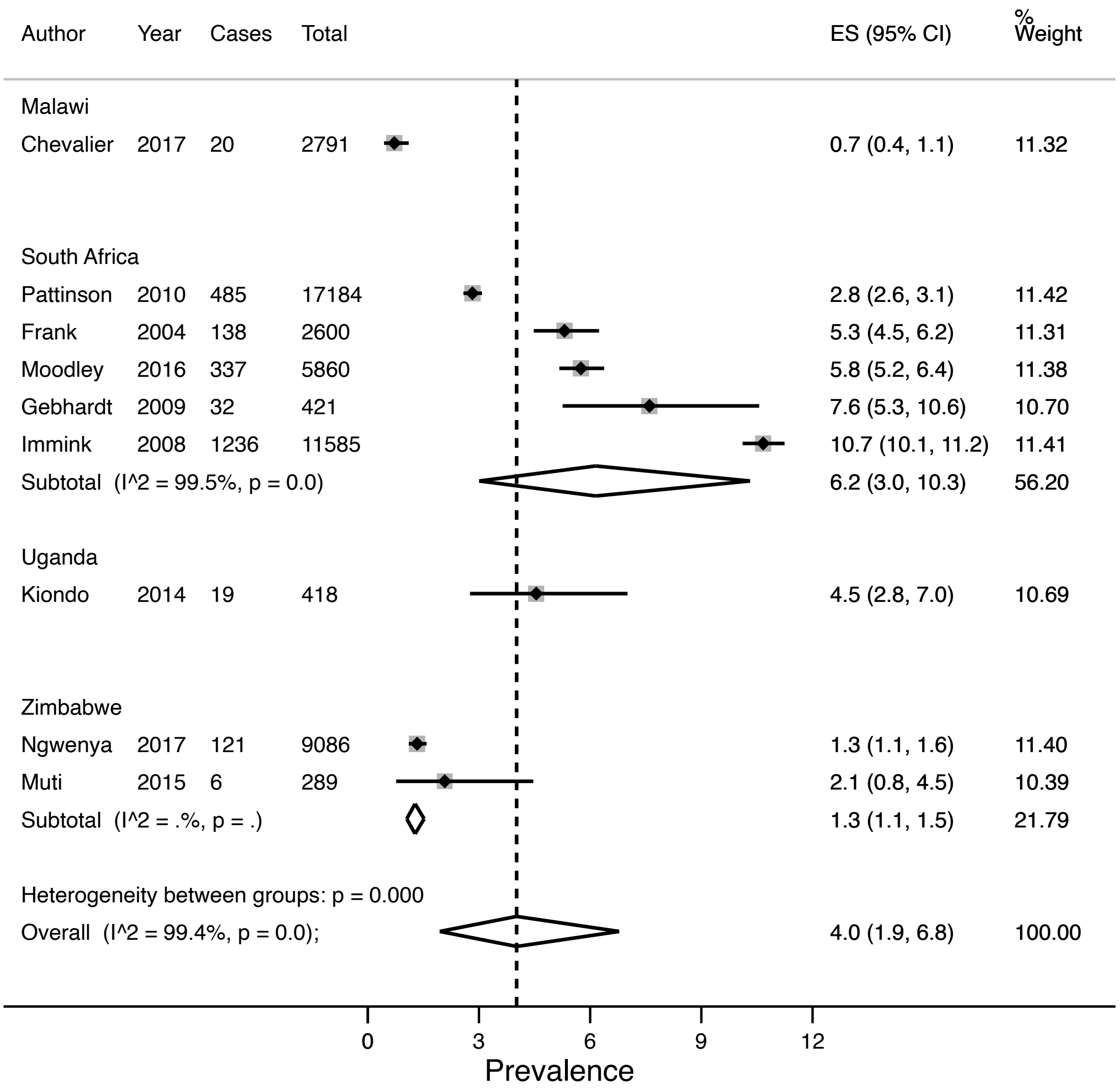

*Moodley 2016 refers to Moodley, 2016b (87)

Figure J.5. Forest Plot Summarizing the Pooled Prevalence of Pre-eclampsia/Eclampsia Among Women Living with HIV

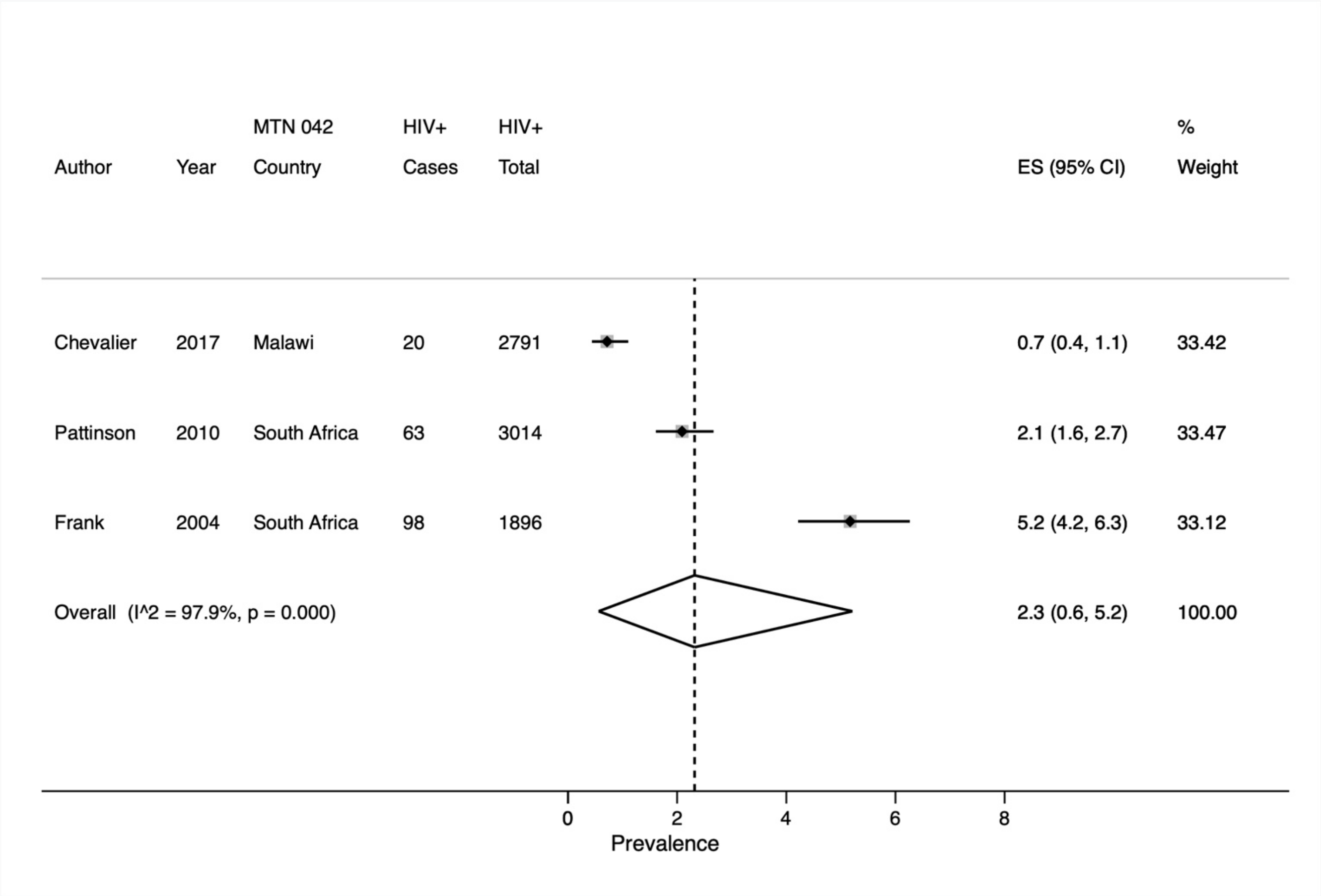

Figure J.6. Forest Plot Summarizing the Pooled Prevalence of Pre-eclampsia/Eclampsia Among HIV Negative Women

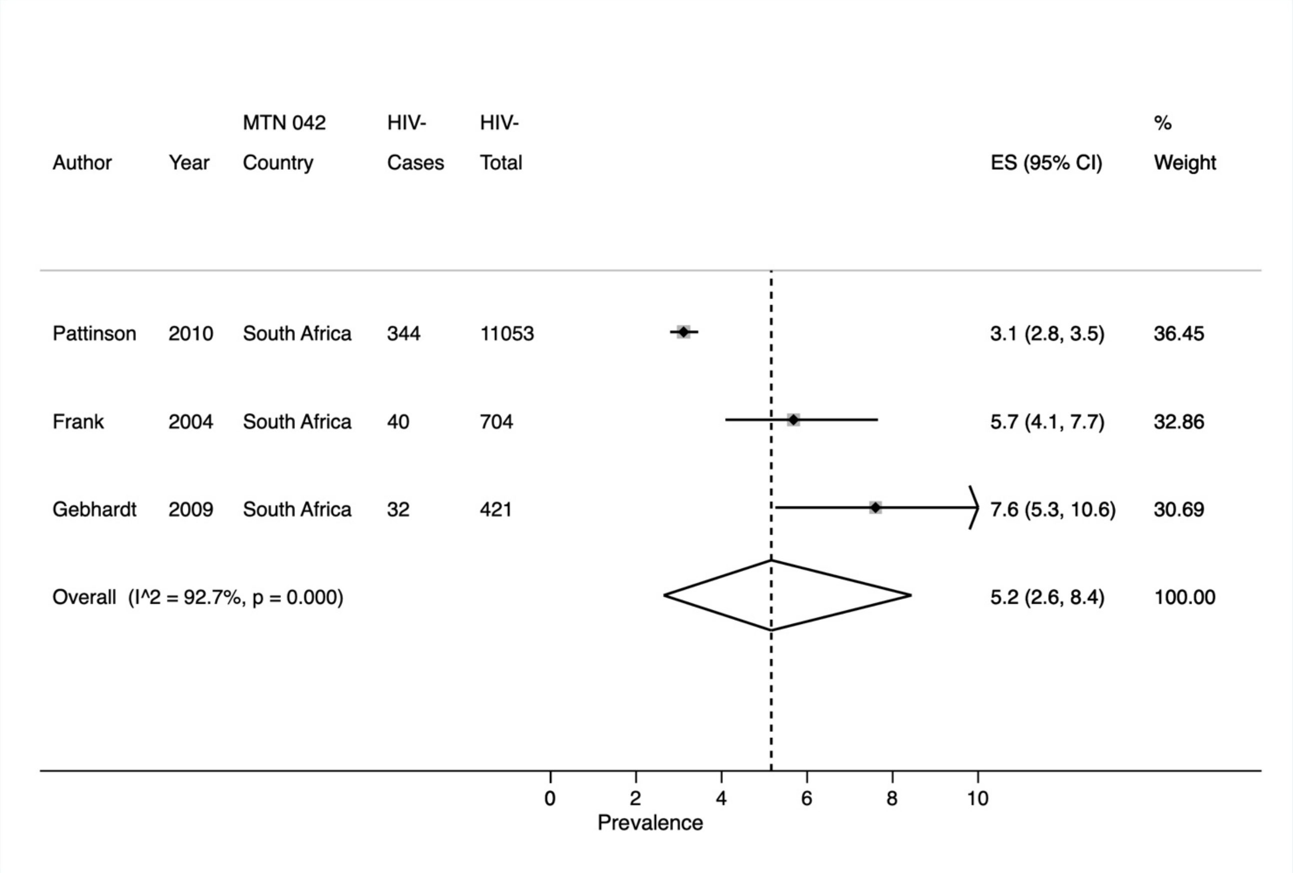

SECTION K: PPROM

Table K.1. Search Result Flow

	N
**Title Review**	50
**Abstract Review**	23
**Manuscripts Reviewed***	9
**Manuscripts Included From Main Search†**	5
**Manuscripts Added From Other Searches**	2
**Total Manuscripts Abstracted**	7
**Total Manuscripts Included For Analysis**	7

* Does not include number of references from systematic reviews reviewed.
† Includes systematic review reference reviews.

Table K.2. Pooled Prevalence of PPROM– Overall, by MTN-042 Country, and by HIV status

	# of estimates*	# of pregnancies/ infants	Mean (Min, Max)	Median (IQR)	Pooled Prevalence (95%CI)	I^2 (p-value)
**Total (all countries)**	7	26,220	3.8% (0.7, 10.0)	2.3% (1.3, 8.1)	2.2% (1.5, 3.2)	93.3% (<0.01)
**Study Countries**	3	6,949	4.5% (0.7, 10.0)	2.9% (0.7, 10.0)	3.1% (0.8, 6.9)	-
**Malawi**	0	-	-	-	-	-
**South Africa**	1	421	0.7% -	-	0.7% (0.1, 2.1)	-
**Uganda**	2	6,528	6.4% (2.9, 10.0)	6.4% (2.9, 10.0)	2.7% (2.3, 3.2)	-
**Zimbabwe**	0	-	-	-	-	-
**Living with HIV**	1	68	10.3% -	-	10.3% (4.2, 20.1)	-
**HIV negative**	2	489	3.3% (0.7, 5.9)	3.3% (0.7, 5.9)	0.9% (0.2, 2.1)	-

* No outliers were identified so no sensitivity analysis excluding outliers was conducted.

Figure K.1. Forest Plot Summarizing the Pooled Prevalence of PPROM

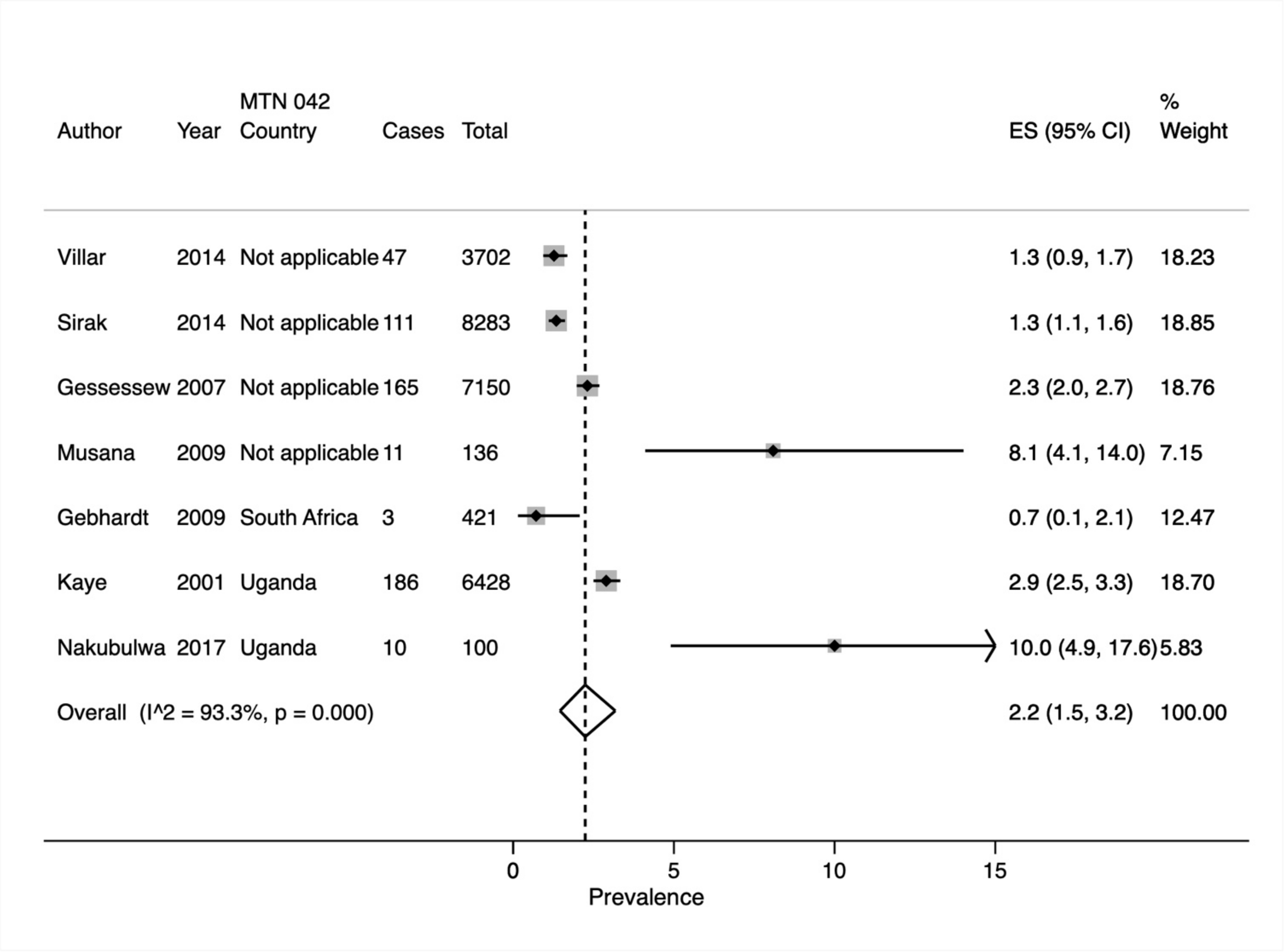

REFERENCES

1. Abdel-Aleem H, Singata M, Abdel-Aleem M, Mshweshwe N, Williams X, Hofmeyr GJ. Uterine massage to reduce postpartum hemorrhage after vaginal delivery. Int J Gynecol Obstet. 2010;111(1):32–6.
2. Ades V, Mwesigwa J, Natureeba P, Clark TD, Plenty A, Charlebois E, et al. Neonatal mortality in HIV-exposed infants born to women receiving combination antiretroviral therapy in rural Uganda. J Trop Pediatr. 2013;59(6):441–6.
3. Ali M, Nelson A, Luquero FJ, Azman AS, Debes AK, M'bang'ombe MM, et al. Safety of a killed oral cholera vaccine (Shanchol) in pregnant women in Malawi: an observational cohort study. Lancet Infect Dis. 2017;17(5):538–44.
4. Allanson ER, Muller M, Pattinson RC. Causes of perinatal mortality and associated maternal complications in a South African province: Challenges in predicting poor outcomes. BMC Pregnancy Childbirth. 2015;15:37.
5. Anderson SM, Naidoo RN, Ramkaran P, Asharam K, Muttoo S, Chuturgoon AA. OGG1 Ser326Cys polymorphism, HIV, obesity and air pollution exposure influences adverse birth outcome susceptibility, within South African Women. Reprod Toxicol. 2018;79:8–15.
6. Arinaitwe E, Ades V, Walakira A, Ninsiima B, Mugagga O, Patil TS, et al. Intermittent Preventive Therapy with Sulfadoxine-Pyrimethamine for Malaria in Pregnancy: A Cross-Sectional Study from Tororo, Uganda. PLoS One. 2013;8(9):e73073.
7. Ashorn P, Alho L, Ashorn U, Cheung YB, Dewey KG, Harjunmaa U, et al. The impact of lipid-based nutrient supplement provision to pregnant women on newborn size in rural Malawi: A randomized controlled trial. Am J Clin Nutr. 2015;101(2):387–97.
8. Asiki G, Baisley K, Newton R, Marions L, Seeley J, Kamali A, et al. Adverse pregnancy outcomes in rural Uganda (1996-2013): trends and associated factors from serial cross sectional surveys. BMC Pregnancy Childbirth. 2015;15(1):1–12.
9. Atukunda EC, Siedner MJ, Obua C, Mugyenyi GR, Twagirumukiza M, Agaba AG. Sublingual Misoprostol versus Intramuscular Oxytocin for Prevention of Postpartum Hemorrhage in Uganda: A Double-Blind Randomized Non-Inferiority Trial. PLoS Med. 2014;11(11):e1001752.
10. Bailey PE, Andualem W, Brun M, Freedman L, Gbangbade S, Kante M, et al. Institutional maternal and perinatal deaths: a review of 40 low and middle income countries. BMC Pregnancy Childbirth. 2017;17:295.
11. Bamigboye AA, Hofmeyr GJ, Merrell D. Rectal misoprostol in the prevention of postpartum hemorrhage. Am J Obstet Gynecol. 1998;179(4):1043–6.
12. Basu JK, Jeketera CM, Basu D. Obesity and its outcomes among pregnant South African women. Int J Gynecol Obstet. 2010;110:101–4.
13. Bebell LM, Ngonzi J, Bazira J, Fajardo Y, Boatin AA, Siedner MJ, et al. Antimicrobial-resistant infections among postpartum women at a Ugandan referral hospital. PLoS One. 2017;12(4):e0175456.
14. Bello B, Kielkowski D, Heederik D, Wilson K. Time-to-pregnancy and pregnancy outcomes in a South African population. BMC Public Health. 2010;10:565.
15. Beltman J, Van Den Akker T, Van Lonkhuijzen L, Schmidt A, Chidakwani R, Van

Roosmalen J. Beyond maternal mortality: obstetric hemorrhage in a Malawian district. Acta Obstet Gynecol Scand. 2011;90:1423–7.

16. Bera E, McCausland K, Nonkwelo R, Mgudlwa B, Chacko S, Majeke B. Birth defects following exposure to efavirenz-based antiretroviral therapy during pregnancy: a study at a regional South African hospital. AIDS. 2010;24(2):283–9.
17. Bloch EM, Crookes RL, Hull J, Fawcus S, Gangaram R, Anthony J, et al. The impact of human immunodeficiency virus infection on obstetric hemorrhage and blood transfusion in South Africa. Transfusion. 2015;55(7):1675–84.
18. Bodkin C, Klopper H, Langley G. A comparison of HIV positive and negative pregnant women at a public sector hospital in South Africa. J Clin Nurs. 2006;15(6):735–41.
19. Brahmbhatt H, Sullivan D, Kigozi G, Askin F, Wabwire-Mangenm F, Serwadda D, et al. Association of HIV and malaria with mother-to-child transmission, birth outcomes, and child mortality. J Acquir Immune Defic Syndr. 2008;47(4):472–6.
20. Braun V, Rempis E, Schnack A, Decker S, Rubaihayo J, Tumwesigye NM, et al. Lack of effect of intermittent preventive treatment for malaria in pregnancy and intense drug resistance in western Uganda. Malar J. 2015;14:372.
21. Brittain K, Myer L, Koen N, Koopowitz S, Donald KA, Barnett W, et al. Risk factors for antenatal depression and associations with infant birth outcomes: Results from a South African birth cohort study. Paediatr Perinat Epidemiol. 2015;29(6):504–14.
22. Byaruhanga RN. Improving healthcare by perinatal mortality audit and feedback. Trop Doct. 2000;30:94–7.
23. Chagomerana MB, Miller WC, Pence BW, Hosseinipour MC, Hoffman IF, Flick RJ, et al. PMTCT Option B+ does not increase preterm birth risk and may prevent extreme prematurity: A retrospective cohort study in Malawi. J Acquir Immune Defic Syndr. 2018;74(4):367–74.
24. Chetty T, Thorne C, Coutsoudis A. Preterm delivery and small-for-gestation outcomes in HIV-infected pregnant women on antiretroviral therapy in rural South Africa: Results from a cohort study, 2010-2015. PLoS One. 2018;13(2):e0192805.
25. Chevalier MS, King CC, Ellington S, Wiener J, Kayira D, Chasela CS, et al. Maternal and neonatal outcomes among women with HIV infection and their infants in Malawi. Int J Gynecol Obstet. 2017;137(3):282–9.
26. Chihana ML, Price A, Floyd S, Mboma S, Mvula H, Branson K, et al. The effect of maternal HIV status on under-five mortality in rural northern Malawi: A prospective cohort study. JAIDS. 2015;68(1):81–90.
27. Colbourn T, Nambiar B, Bondo A, Makwenda C, Tsetekani E, Makonda-Ridley A, et al. Effects of quality improvement in health facilities and community mobilization through women’s groups on maternal, neonatal and perinatal mortality in three districts of Malawi: MaiKhanda, a cluster randomized controlled effectiveness trial. Int Health. 2013;5(3):180–95.
28. Coutsoudis A, Pillay K, Spooner E, Kuhn L, Coovadia HM. Randomized trial testing the effect of vitamin A supplementation on pregnancy outcomes and early mother-to-child HIV-1 transmission in Durban, South Africa. Aids. 1999;13(12):1517–24.
29. Cutland CL, Madhi SA, Zell ER, Kuwanda L, Laque M, Groome M, et al.

Chlorhexidine maternal-vaginal and neonate body wipes in sepsis and vertical transmission of pathogenic bacteria in South Africa: a randomised, controlled trial. Lancet. 2009;374(9705):1909–16.
30. Dow A, Kayira D, Hudgens MG, Van Rie A, King CC, Ellington S, et al. The effect of cotrimoxazole prophylactic treatment on malaria, birth outcomes, and postpartum CD4 count in HIV-infected women. Infect Dis Obstet Gynecol. 2013;2013:340702.
31. Dreise M, Galiwango G, Hodges A. Incidence of cleft lip and palate in Uganda. Cleft Palate-Craniofacial J. 2011;48(2):156–60.
32. Ebrahim S, Daponte A, Guidozzi F. The impact of free antenatal care on perinatal mortality. Int J Gynecol Obstet. 2000;71(3):205–7.
33. Ellison G, de Wet T, Matshidze KP, Cooper P. The reliability and validity of self-reported reproductive history and obstetric morbidity amongst Birth to Ten mothers in Soweto. Curationis. 2000;23(4):76–80.
34. Fall CHD, Sachdev HS, Osmond C, Restrepo-Mendez MC, Victora C, Martorell R, et al. Association between maternal age at childbirth and child and adult outcomes in the offspring: A prospective study in five low-income and middle-income countries (COHORTS collaboration). Lancet Glob Heal. 2015;3(7):e366–77.
35. Fatti G, Shaikh N, Eley B, Grimwood A. Effectiveness of community-based support for pregnant women living with HIV: A cohort study in South Africa. AIDS Care. 2016;28(S1):114–8.
36. Feng G, Simpson JA, Chaluluka E, Molyneux ME, Rogerson SJ. Decreasing burden of malaria in pregnancy in Malawian women and its relationship to use of intermittent preventive therapy or bed nets. PLoS One. 2010;5(8):e12012.
37. Feresu S, Harlow S, Gillespie B, Welch K, Johnson T. Birthweight-adjusted Dubowitz methods: reducing misclassification of assessments of gestational age in a Zimbabwean population. Cent Afr J Med. 2003;49(5–6):47–53.
38. Feresu SA, Harlow SD, Welch K, Gillespie BW. Incidence of and socio-demographic risk factors for stillbirth, preterm birth and low birthweight among Zimbabwean women. Paediatr Perinat Epidemiol. 2004;18(2):154–63.
39. Feresu SA, Harlow SD, Welch K, Gillespie BW. Incidence of stillbirth and perinatal mortality and their associated factors among women delivering at Harare Maternity Hospital, Zimbabwe: A cross-sectional retrospective analysis. BMC Pregnancy Childbirth. 2005;5:9.
40. Feresu SA, Harlow SD, Woelk GB. Risk factors for low birthweight in Zimbabwean women: A secondary data analysis. PLoS One. 2015;10(6):e0129705.
41. Filler SJ, Kazembe P, Thigpen M, Macheso A, Parise ME, Newman RD, et al. Randomized Trial of 2-Dose versus Monthly Sulfadoxine-Pyrimethamine Intermittent Preventive Treatment for Malaria in HIV-Positive and HIV-Negative Pregnant Women in Malawi. J Infect Dis. 2006;194(3):286–93.
42. Frank KA, Buchmann EJ, Schackis RC. Does human immunodeficiency virus infection protect against preeclampsia-eclampsia? Obstet Gynecol. 2004;104(2):238–42.
43. Friis H, Gomo E, Nyazema N, Ndhlovu P, Krarup H, Kæstel P, et al. Effect of multimicronutrient supplementation on gestational length and birth size: A

randomized, placebo-controlled, double-blind effectiveness trial in Zimbabwe. Am J Clin Nutr. 2004;80:178–84.
44. Friis H, Gomo E, Mashange W, Nyazema N, Kostel P, Wieringa F, et al. The acute phase response to parturition: a cross-sectional study in Zimbabwe. Afr J Reprod Health. 2009;13(2):61–8.
45. Gebhardt S, Bruiners N, Hillermann R. A novel exonic variant (221delT) in the LGALS13 gene encoding placental protein 13 (PP13) is associated with preterm labour in a low risk population. J Reprod Immunol. 2009;82(2):166–73.
46. Gessessew A. Maternal Complications in a Zonal Hospital. Ethiop Med J. 2007;45(1):47–54.
47. Gibb DM, Kizito H, Russell EC, Chidziva E, Zalwango E, Nalumenya R, et al. Pregnancy and infant outcomes among HIV-infected women taking long-term art with and without tenofovir in the DART trial. PLoS Med. 2012;9(5):e1001217.
48. Govender I. Auditing stillbirths at Lower Umfolozi War Memorial Regional Hospital: A 12-month review. South African Med J. 2017;107(12):1121–6.
49. Gray RH, Wabwire-Mangen F, Kigozi G, Sewankambo NK, Serwadda D, Moulton LH, et al. Randomized trial of presumptive sexually transmitted disease therapy during pregnancy in Rakai, Uganda. Am J Obstet Gynecol. 2001;185(5):1209–17.
50. Gülmezoglu AM, Villar J, Ngoc NTN, Piaggio G, Carroli G, Adetoro L, et al. WHO multicentre randomised trial of misoprostol in the management of the third stage of labour. Lancet. 2001;358:689–95.
51. Gumede S, Black V, Naidoo N, Chersich MF. Attendance at antenatal clinics in inner-city Johannesburg, South Africa and its associations with birth outcomes: analysis of data from birth registers at three facilities. BMC Public Health. 2017;17(Suppl 3):443.
52. Gutman J, Mwandama D, Wiegand R, Ali D, Mathanga D, Skarbinski J. Effectiveness of Intermittent Preventive Treatment With Sulfadoxine-Pyrimethamine During Pregnancy on Maternal and Birth Outcomes in Machinga District, Malawi. J Infect Dis. 2013;208(6):907–16.
53. Hall D, Gebhardt S, Theron G, Grové D. Pre-eclampsia and gestational hypertension are less common in HIV infected women. Pregnancy Hypertens. 2014;4:91–6.
54. Harrison V, Fawcus S, Jordaan E. Magnesium supplementation and perinatal hypoxia: outcome of a parallel group randomised trial in pregnancy. BJOG. 2007;114:994–1002.
55. Hjertholm KG, Iversen PO, Holmboe-Ottesen G, Mdala I, Munthali A, Maleta K, et al. Maternal dietary intake during pregnancy and its association to birth size in rural Malawi: A cross-sectional study. Matern Child Nutr. 2018;14:e12433.
56. Hofmeyr GJ, Nikodem VC, De Jager M, Gelbart BR. A randomised placebo controlled trial of oral misoprostol in the third stage of labour. BJOG An Int J Obstet Gynaecol. 1998;105:971–5.
57. Hussain A, Moodley D, Naidoo S, Esterhuizen TM. Pregnant women’s access to PMTCT and ART services in South Africa and implications for universal antiretroviral treatment. PLoS One. 2011;6(12):e27907.
58. Immink A, Scherjon S, Wolterbeek R, Steyn DW. Seasonal influence on the admittance of pre-eclampsia patients in Tygerberg hospital. Acta Obstet Gynecol

Scand. 2008;87(1):36–42.
59. Kalanda BF, Verhoeff FH, Chimsuku L, Harper G, Brabin BJ. Adverse birth outcomes in a malarious area. Epidemiol Infect. 2006;134(3):659–66.
60. Kalilani L, Mofolo I, Chaponda M, Rogerson SJ, Meshnick SR. The effect of timing and frequency of Plasmodium falciparum infection during pregnancy on the risk of low birth weight and maternal anemia. Trans R Soc Trop Med Hyg. 2010;104(6):416–22.
61. Kalumbi C, Tadesse E. An audit of deliveries and outcome at Queen Elizabeth Central Hospital, Blantyre, in 1999. Malawi Med J. 2001;13(3):34–5.
62. Kananura RM, Tetui M, Mutebi A, Bua JN, Waiswa P, Kiwanuka SN, et al. The neonatal mortality and its determinants in rural communities of Eastern Uganda. Reprod Health. 2016;13:13.
63. Kananura RM, Wamala R, Ekirapa-Kiracho E, Tetui M, Kiwanuka SN, Waiswa P, et al. A structural equation analysis on the relationship between maternal health services utilization and newborn health outcomes: A cross-sectional study in Eastern Uganda. BMC Pregnancy Childbirth. 2017;17:98.
64. Kapisi J, Kakuru A, Jagannathan P, Muhindo MK, Natureeba P, Awori P, et al. Relationships between infection with Plasmodium falciparum during pregnancy, measures of placental malaria, and adverse birth outcomes. Malar J. 2017;16:400.
65. Kasumba IN, Nalunkuma AJ, Mujuzi G, Kitaka FS, Byaruhanga R, Okong P, et al. Low birthweight associated with maternal anaemia and Plasmodium falciparum infection during pregnancy, in a peri-urban/urban area of low endemicity in Uganda. Ann Trop Med Parasitol. 2000;94(1):7–13.
66. Kaye DK. Risk Factors for Preterm Premature Rupture of Membranes at Mulago Hospital, Kampala. East Afr Med J. 2001;78(2):65–9.
67. Kaye DK, Mirembe FM, Bantebya G, Johansson A, Ekstrom AM. Domestic violence during pregnancy and risk of low birthweight and maternal complications: A prospective cohort study at Mulago Hospital, Uganda. Trop Med Int Heal. 2006;11(10):1576–84.
68. Kesande T, Muwazi LM, Bataringaya A, Rwenyonyi CM. Prevalence, pattern and perceptions of cleft lip and cleft palate among children born in two hospitals in Kisoro District, Uganda. BMC Oral Health. 2014;14:104.
69. Kimani J, Phiri K, Kamiza S, Duparc S, Ayoub A, Rojo R, et al. Efficacy and Safety of Azithromycin-Chloroquine versus Sulfadoxine-Pyrimethamine for Intermittent Preventive Treatment of Plasmodium falciparum Malaria Infection in Pregnant Women in Africa: An Open-label, Randomized Trial. PLoS One. 2016;11(6):e0157045.
70. Kiondo P, Wamuyu-Maina G, Wandabwa J, Bimenya GS, Tumwesigye NM, Okong P. The effects of vitamin C supplementation on pre-eclampsia in Mulago Hospital, Kampala, Uganda: A randomized placebo controlled clinical trial. BMC Pregnancy Childbirth. 2014;14:283.
71. Kujala S, Waiswa P, Kadobera D, Akuze J, Pariyo G, Hanson C. Trends and risk factors of stillbirths and neonatal deaths in Eastern Uganda (1982–2011): a cross-sectional, population-based study. Trop Med Int Heal. 2017;22(1):63–73.
72. Kulmala T, Vaahtera M, Ndekha M, Koivisto AM, Cullinan T, Salin ML, et al. The

importance of preterm births for peri- and neonatal mortality in rural Malawi. Paediatr Perinat Epidemiol. 2000;14(3):219–26.
73. Kumwenda N, Miotti PG, Taha TE, Broadhead R, Biggar RJ, Jackson JB, et al. Antenatal Vitamin A Supplementation Increases Birth Weight and Decreases Anemia among Infants Born to Human Immunodeficiency Virus–Infected Women in Malawi. Clin Infect Dis. 2002;35(5):618–24.
74. Kundodyiwa TW, Majoko F, Rusakaniko S. Misoprostol versus oxytocin in the third stage of labor. Int J Gynecol Obstet. 2001;75(3):235–41.
75. Lavin T, Preen DB, Pattinson R. Timing and cause of perinatal mortality for small-for-gestational-age babies in South Africa: critical periods and challenges with detection. Matern Heal Neonatol Perinatol. 2016;2:11.
76. Lewycka S, Mwansambo C, Rosato M, Kazembe P, Phiri T, Mganga A, et al. Effect of women's groups and volunteer peer counselling on rates of mortality, morbidity, and health behaviours in mothers and children in rural Malawi (MaiMwana): A factorial, cluster-randomised controlled trial. Lancet. 2013;381(9879):1721–35.
77. Liu K, Farahani M, Mashama T, Mawela M, Joseph J, van Schaik N, et al. Pregnancy outcomes and birth defects from an antiretroviral drug safety study of women in South Africa and Zambia. AIDS. 2014;28(15):2259–68.
78. Luntamo M, Kulmala T, Mbewe B, Cheung YB, Maleta K, Ashorn P. Effect of repeated treatment of pregnant women with sulfadoxine- pyrimethamine and azithromycin on preterm delivery in Malawi: A randomized controlled trial. Am J Trop Med Hyg. 2010;83(6):1212–20.
79. Madhi SA, Cutland CL, Jose L, Koen A, Govender N, Wittke F, et al. Safety and immunogenicity of an investigational maternal trivalent group B streptococcus vaccine in healthy women and their infants: a randomised phase 1b/2 trial. Lancet Infect Dis. 2016;16(8):923–34.
80. Majoko F, Nyström L, Munjanja S, Lindmark G. Usefulness of risk scoring at booking for antenatal care in predicting adverse pregnancy outcome in a rural African setting. J Obstet Gynaecol (Lahore). 2002;22(6):604–9.
81. Majoko F, Munjanja SP, Nyström L, Mason E, Lindmark G. Randomised controlled trial of two antenatal care models in rural Zimbabwe. BJOG. 2007;114(7):802–11.
82. Makanani B, Balkus JE, Jiao Y, Noguchi LM, Palanee-Phillips T, Mbilizi Y, et al. Pregnancy and Infant Outcomes Among Women Using the Dapivirine Vaginal Ring in Early Pregnancy. J Acquir Immune Defic Syndr. 2018;79(5):566–72.
83. Malaba TR, Phillips T, Le Roux S, Brittain K, Zerbe A, Petro G, et al. Antiretroviral therapy use during pregnancy and adverse birth outcomes in South African women. Int J Epidemiol. 2017;46(5):1678–89.
84. McDonald CR, Conroy AL, Gamble JL, Papp E, Hawkes M, Olwoch P, et al. Estradiol Levels Are Altered in Human Immunodeficiency Virus-Infected Pregnant Women Randomized to Efavirenz-Versus Lopinavir/Ritonavir-Based Antiretroviral Therapy. Clin Infect Dis. 2018;66(3):428–36.
85. Metaferia AM, Muula AS. Stillbirths and hospital early neonatal deaths at Queen Elizabeth Central Hospital, Blantyre-Malawi. Int Arch Med. 2009;2:25.
86. Moodley T, Moodley D, Sebitloane M, Maharaj N, Sartorius B. Improved

pregnancy outcomes with increasing antiretroviral coverage in South Africa. BMC Pregnancy Childbirth. 2016;16:35.
87. Moodley J, Onyangunga OA, Maharaj NR. Hypertensive disorders in primigravid black South African women: A one-year descriptive analysis. Hypertens Pregnancy. 2016;35(4):529–35.
88. Moodley D, Sartorius B, Madurai S, Chetty V, Maman S. Pregnancy Outcomes in Association with STDs including genital HSV-2 shedding in a South African Cohort Study. Sex Transm Infect. 2017;93(7):460–6.
89. Moyer C, Kolars C, Opping S, Bakari A, Bell A, Busingye P. Predictors of stillbirths and neonatal deaths in rural western Uganda Cheryl. Int J Gynecol Obstet. 2016;134(3):190–3.
90. Msamati BC, Igbigbi PS, Chisi JE. The incidence of cleft lip, cleft palate, hydrocephalus, and spina bifida at Queen Elizabeth Central Hospital, Blantyre, Malawi. Cent Afr J Med. 2000;46(11):292–6.
91. Murray J, Eskenazi B, Bornman R, Gaspar FW, Crause M, Obida M, et al. Exposure to DDT and hypertensive disorders of pregnancy among South African women from an indoor residual spraying region: The VHEMBE study. Environ Res. 2018;162:49–54.
92. Musana J, Ojwang S, Khisa W, Kiarie J. Pregnancy outcomes in mothers with advanced human immunodeficiency virus disease. East Afr Med J. 2009;86(10):480–5.
93. Muti M, Tshimanga M, Notion GT, Bangure D, Chonzi P. Prevalence of pregnancy induced hypertension and pregnancy outcomes among women seeking maternity services in Harare, Zimbabwe. BMC Cardiovasc Disord. 2015;15:111.
94. Mwanyumba F, Inion I, Gaillard P, Mandaliya K, Praet M, Temmerman M. Placental inflammation and perinatal outcome. Eur J Obstet Gynecol Reprod Biol. 2003;108(2):164–70.
95. Msyamboza KP, Savage EJ, Kazembe PN, Gies S, Kalanda G, D'Alessandro U, et al. Community-based distribution of sulfadoxine-pyrimethamine for intermittent preventive treatment of malaria during pregnancy improved coverage but reduced antenatal attendance in southern Malawi. Trop Med Int Heal. 2009;14(2):183–9.
96. Nabiwemba E, Marchant T, Namazzi G, Kadobera D, Waiswa P. Identifying high-risk babies born in the community using foot length measurement at birth in Uganda. Child Care Health Dev. 2013;39(1):20–6.
97. Naidoo S, London L, Burdorf A, Naidoo R, Kromhout H. Spontaneous miscarriages and infant deaths among female farmers in rural South Africa. Scand J Work Environ Heal. 2011;37(3):227–36.
98. Naidu S, Moodley J, Adhikari M, Ramsaroop R, Morar N, Dunmoye OO. Clinico-pathological study of causes of perinatal mortality in a developing country. J Obstet Gynaecol (Lahore). 2001;21(5):443–7.
99. Nakubulwa S, Kaye DK, Bwanga F, Tumwesigye NM, Nakku-Joloba E, Mirembe F. Effect of suppressive acyclovir administered to HSV-2 positive mothers from week 28 to 36 weeks of pregnancy on adverse obstetric outcomes: A double-blind randomised placebo-controlled trial. Reprod Health. 2017;14:31.
100. Nankabirwa V, Tumwine JK, Tylleskär T, Nankunda J, Sommerfelt H. Perinatal mortality in Eastern Uganda: A community based prospective cohort study. PLoS

One. 2011;6(5):e19674.
101. Nankabirwa V, Tumwine JK, Mugaba PM, Tylleskär T, Sommerfelt H. Child survival and BCG vaccination: A community based prospective cohort study in Uganda. BMC Public Health. 2015;15:175.
102. Ndibazza J, Muhangi L, Akishule D, Kiggundu M, Ameke C, Oweka J, et al. Effects of Deworming during Pregnancy on Maternal and Perinatal Outcomes in Entebbe, Uganda: A Randomized Controlled Trial. Clin Infect Dis. 2010;50(4):531–40.
103. Ndibazza J, Lule S, Nampijja M, Mpairwe H, Oduru G, Kiggundu M, et al. A description of congenital anomalies among infants in Entebbe, Uganda. Birth Defects Res Part A. 2011;91(9):857–61.
104. Ndirangu J, Newell ML, Bland RM, Thorne C. Maternal HIV infection associated with small-for-gestational age infants but not preterm births: Evidence from rural South Africa. Hum Reprod. 2012;27(6):1846–56.
105. Ndyomugyenyi R, Magnussen. Chloroquine prophylaxis, iron-folic acid supplementation or case management of malaria attacks in primigravidae in western Uganda: effects on maternal parasitaemia and haemoglobin levels and on birthweight. Trans R Soc Trop Med Hyg. 2000;94:413–8.
106. Ndyomugyenyi R, Magnussen P. Malaria morbidity, mortality and pregnancy outcome in areas with different levels of malaria transmission in Uganda: A hospital record-based study. Trans R Soc Trop Med Hyg. 2001;95(5):463–8.
107. Ndyomugyenyi R, Kabatereine N, Olsen A, Magnussen P. Efficacy of ivermectin and albendazole alone and in combination for treatment of soil-transmitted helminths in pregnancy and adverse events: A randomized open label controlled intervention trial in Masindi District, Western Uganda. Am J Trop Med Hyg. 2008;79(6):856–63.
108. Ndyomugyenyi R, Clarke SE, Hutchison CL, Hansen KS, Magnussen P. Efficacy of malaria prevention during pregnancy in an area of low and unstable transmission: An individually-randomised placebo-controlled trial using intermittent preventive treatment and insecticide-treated nets in the Kabale Highlands, Southwestern Uga. Trans R Soc Trop Med Hyg. 2011;105(11):607–16.
109. Ngwenya S. Postpartum hemorrhage: Incidence, risk factors, and outcomes in a low-resource setting. Int J Womens Health. 2016;8:647–50.
110. Ngwenya S. Severe preeclampsia and eclampsia: Incidence, complications, and perinatal outcomes at a low-resource setting, mpilo central hospital, bulawayo, Zimbabwe. Int J Womens Health. 2017;9:353–7.
111. Njiru J, Biryabarema C, Kagawa M. Fetal outcomes among grand multiparous and multiparous women in Mulago hospital, Uganda. East Afr Med J. 2013;90(3):84–8.
112. Nkhoma ET, Kalilani-Phiri L, Mwapasa V, Rogerson SJ, Meshnick SR. Effect of HIV infection and Plasmodium falciparum parasitemia on pregnancy outcomes in Malawi. Am J Trop Med Hyg. 2012;87(1):29–34.
113. Noble AJ, Ning Y, Woelk GBG, Mahomed K, Williams MAM. Preterm delivery risk in relation to maternal HIV infection, history of malaria and other infections among urban Zimbabwean women. Cent Afr J Med. 2005;51(5/6):53–8.

114. Ntuli ST, Malangu N. An investigation of the stillbirths at a tertiary hospital in Limpopo province of South Africa. Glob J Health Sci. 2012;4(6):141–7.
115. Ogbonna C, Woelk GB, Ning Y, Mudzamiri S, Mahomed K, Williams MA. Maternal mid-arm circumference and other anthropometric measures of adiposity in relation to infant birth size among Zimbabwean women. Acta Obstet Gynecol Scand. 2007;86(1):26–32.
116. Ononge S, Mirembe F, Wandabwa J, Campbell OMR. Incidence and risk factors for postpartum hemorrhage in Uganda. Reprod Health. 2016;13:38.
117. Pattinson RC. Why babies die - a perinatal care survey of South Africa, 2000 - 2002. S Afr J Obstet Gynaecol. 2003;9(3):58–63.
118. Pattinson RC, Hulsbergen MH, Van Hoorick L. The effect of maternal HIV infection on maternal conditions and perinatal deaths in southwest Tshwane. Facts, Views Vis ObGyn. 2010;2(4):227–31.
119. Rempis EM, Schnack A, Decker S, Braun V, Rubaihayo J, Tumwesigye NM, et al. Option B+ for prevention of vertical HIV transmission has no influence on adverse birth outcomes in a cross-sectional cohort in Western Uganda. BMC Pregnancy Childbirth. 2017;17:82.
120. Roh ME, Shiboski S, Natureeba P, Kakuru A, Muhindo M, Ochieng T, et al. Protective Effect of Indoor Residual Spraying of Insecticide on Preterm Birth among Pregnant Women with HIV Infection in Uganda: A Secondary Data Analysis. J Infect Dis. 2017;216(12):1541–9.
121. Röllin HB, Kootbodien T, Channa K, Odland J. Prenatal exposure to cadmium, placental permeability and birth outcomes in coastal populations of South Africa. PLoS One. 2015;10(11):e0142455.
122. Rollins NC, Coovadia HM, Bland RM, Coutsoudis A, Bennish ML, Patel D, et al. Pregnancy outcomes in HIV-infected and uninfected women in rural and urban South Africa. J Acquir Immune Defic Syndr. 2007;44(3):321–8.
123. Sania A, Brittain K, Phillips TK, Zerbe A, Ronan A, Myer L, et al. Effect of alcohol consumption and psychosocial stressors on preterm and small-for-gestational-age births in HIV-infected women in South Africa: A cohort study. BMJ Open. 2017;7(3):e014293.
124. Sayed AR, Bourne D, Pattinson R, Nixon J, Henderson B. Decline in the prevalence of neural tube defects following folic acid fortification and its cost-benefit in South Africa. Birth Defects Res Part A - Clin Mol Teratol. 2008;82(4):211–6.
125. Schrag SJ, Cutland CL, Zell ER, Kuwanda L, Buchmann EJ, Velaphi SC, et al. Risk factors for neonatal sepsis and perinatal death among infants enrolled in the prevention of perinatal sepsis trial, Soweto, South Africa. Pediatr Infect Dis J. 2012;31(8):821–6.
126. Sebitloane HM, Moodley J, Esterhuizen TM. Prophylactic antibiotics for the prevention of postpartum infectious morbidity in women infected with human immunodeficiency virus: a randomized controlled trial. Am J Obstet Gynecol. 2008;198(2):189.e1-189.e6.
127. Sebitloane HM, Moodley J. Maternal and obstetric complications among HIV-infected women treated with highly active antiretroviral treatment at a Regional Hospital in Durban, South Africa. Niger J Clin Pract. 2017;20(11):1360–7.

128. Shabalala E, Sebitloane HM. An increase in rates of obstetric haemorrhage in a setting of high HIV seroprevalence. South African Med J. 2017;107(7):602–5.
129. Shamu S, Munjanja S, Zarowsky C, Shamu P, Temmerman M, Abrahams N. Intimate partner violence, forced first sex and adverse pregnancy outcomes in a sample of Zimbabwean women accessing maternal and child health care. BMC Public Health. 2018;18(1):595.
130. Sibeko S, Baxter C, Yende N, Karim QA, Abdool Karim SS. Contraceptive Choices, Pregnancy Rates, and Outcomes in a Microbicide Trial. Obs Gynecol. 2011;118(4):895–904.
131. Sirak B, Mesfin E. Maternal and Perinatal Outcome of Pregnancies With Preterm Premature Rupture of Membranes (PPROM) At Tikur Anbessa Specialized Teaching Hospital, Addis Ababa, Ethiopia. Ethiop Med J. 2014;52(4):165–72.
132. Steyn K, De Wet T, Saloojee Y, Nel H, Yach D. The influence of maternal cigarette smoking, snuff use and passive smoking on pregnancy outcomes: The Birth to Ten Study. Paediatr Perinat Epidemiol. 2006;20(2):90–9.
133. Sullivan AD, Nyirenda T, Cullinan T, Taylor T, Harlow SD, James SA, et al. Malaria Infection during Pregnancy: Intrauterine Growth Retardation and Preterm Delivery in Malawi. J Infect Dis. 1999;179(6):1580–3.
134. Taha TE, Yende-Zuma N, Aizire J, Chipato T, Wambuzi Ogwang L, Makanani B, et al. The multi-country PROMOTE HIV antiretroviral treatment observational cohort in Sub-Saharan Africa: Objectives, design, and baseline findings. PLoS One. 2018;13(12):e0208805.
135. Talip Q, Theron G, Steyn W, Hall D. Total perinatally related losses at Tygerberg hospital -A comparison between 1986, 1993 and 2006. South African Med J. 2010;100(4):250–3.
136. Taylor SM, Madanitsa M, Thwai KL, Khairallah C, Kalilani-Phiri L, Van Eijk AM, et al. Minimal impact by antenatal subpatent Plasmodium falciparum infections on delivery outcomes in Malawian women: A cohort study. J Infect Dis. 2017;216(3):296–304.
137. Theron A, Loveland J. Birth prevalence of anorectal malformation in the referral area for the University of the Witwatersrand Tertiary Hospitals, South Africa. Eur J Pediatr Surg. 2015;25(2):220–5.
138. Ticconi C, Mapfumo M, Dorrucci M, Naha N, Tarira E, Pietropolli A, et al. Effect of Maternal HIV and Malaria Infection on Pregnancy and Perinatal Outcome in Zimbabwe. J Acquir Immune Defic Syndr. 2003;34(3):289–94.
139. Ticconi C, Arpino C, Longo B, Mapfumo M. Prevalence and risk factors for low birth weight in Northern Zimbabwe. Int J Gynecol Obstet. 2005;88(2):146–7.
140. Turner AN, Tabbah S, Mwapasa V, Rogerson SJ, Meshnick R, Ackerman IV W, et al. Birth outcomes in Malawian women : a cohort study. J Acquir Immune Defic Syndr. 2013;64(4):392–9.
141. Turton M, Africa CWJ. Further evidence for periodontal disease as a risk indicator for adverse pregnancy outcomes. Int Dent J. 2017;67(3):148–56.
142. van den Broek N, Ntonya C, Kayira E, White S, Neilson JP. Preterm birth in rural Malawi: High incidence in ultrasound-dated population. Hum Reprod. 2005;20(11):3235–7.
143. van den Broek NR, White SA, Goodall M, Ntonya C, Kayira E, Kafulafula G, et al.

The APPLe study: A randomized, community-based, placebo-controlled trial of azithromycin for the prevention of preterm birth, with meta-analysis. PLoS Med. 2009;6(12):e1000191.
144. van den Broek NR, White SA, Ntonya C, Ngwale M, Cullinan TR, Molyneux ME, et al. Reproductive health in rural Malawi: A population-based survey. BJOG. 2003;110(10):902–8.
145. van der Merwe K, Hoffman R, Black V, Chersich M, Coovadia A, Rees H. Birth outcomes in South African women receiving highly active antiretroviral therapy: A retrospective observational study. J Int AIDS Soc. 2011;14(1):42.
146. Verhoeff FH, Brabin BJ, Chimsuku L, Kazembe P, Broadhead RL. Malaria in pregnancy and its consequences for the infant in rural Malawi. Ann Trop Med Parasitol. 1999;93(Supp No. 1):S25–33.
147. Villar J, Ismail LC, Victora CG, Ohuma EO, Bertino E, Altman DG, et al. International standards for newborn weight, length, and head circumference by gestational age and sex: The Newborn Cross-Sectional Study of the INTERGROWTH-21st Project. Lancet. 2014;384(9946):857–68.
148. Wabwire-Mangen F, Gray R, Mmiro F, Ndugwa C, Abramowsky C, Wabinga H, et al. Placental membrane inflammation and risks of maternal-to-child transmission of HIV-1 in Uganda. JAIDS. 1999;22(379–385).
149. Walter J, Mwiya M, Scott N, Kasonde P, Sinkala M, Kankasa C, et al. Reduction in Preterm Delivery and Neonatal Mortality after the Introduction of Antenatal Cotrimoxazole Prophylaxis among HIV-Infected Women with Low CD4 Cell Counts. J Infect Dis. 2006;194(11):1510–8.
150. Wanyama R, Kagawa MN, Opio KC, Baingana RK. Effect of maternal Helicobacter Pylori infection on birth weight in an urban community in Uganda. BMC Pregnancy Childbirth. 2016;16(1):158.
151. Worku AG, Yalew AW, Afework MF. The contributions of maternity care to reducing adverse pregnancy outcomes: A cohort study in Dabat District, Northwest Ethiopia. Matern Child Health J. 2014;18(6):1336–44.
152. Young S, Murray K, Mwesigwa J, Natureeba P, Osterbauer B, Achan J, et al. Maternal nutritional status predicts adverse birth outcomes among HIV-infected rural Ugandan women receiving combination antiretroviral therapy. PLoS One. 2012;7(8):e41934.
